# Search for an additional, heavy Higgs boson in the $H\rightarrow ZZ$ decay channel at $\sqrt{s} = 8\;\text{ TeV }$ in $pp$ collision data with the ATLAS detector

**DOI:** 10.1140/epjc/s10052-015-3820-z

**Published:** 2016-01-25

**Authors:** Atlas Collaboration, G. Aad, B. Abbott, J. Abdallah, O. Abdinov, R. Aben, M. Abolins, O. S. AbouZeid, H. Abramowicz, H. Abreu, R. Abreu, Y. Abulaiti, B. S. Acharya, L. Adamczyk, D. L. Adams, J. Adelman, S. Adomeit, T. Adye, A. A. Affolder, T. Agatonovic-Jovin, J. Agricola, J. A. Aguilar-Saavedra, S. P. Ahlen, F. Ahmadov, G. Aielli, H. Akerstedt, T. P. A. Åkesson, A. V. Akimov, G. L. Alberghi, J. Albert, S. Albrand, M. J. Alconada Verzini, M. Aleksa, I. N. Aleksandrov, C. Alexa, G. Alexander, T. Alexopoulos, M. Alhroob, G. Alimonti, L. Alio, J. Alison, S. P. Alkire, B. M. M. Allbrooke, P. P. Allport, A. Aloisio, A. Alonso, F. Alonso, C. Alpigiani, A. Altheimer, B. Alvarez Gonzalez, D. Álvarez Piqueras, M. G. Alviggi, B. T. Amadio, K. Amako, Y. Amaral Coutinho, C. Amelung, D. Amidei, S. P. Amor Dos Santos, A. Amorim, S. Amoroso, N. Amram, G. Amundsen, C. Anastopoulos, L. S. Ancu, N. Andari, T. Andeen, C. F. Anders, G. Anders, J. K. Anders, K. J. Anderson, A. Andreazza, V. Andrei, S. Angelidakis, I. Angelozzi, P. Anger, A. Angerami, F. Anghinolfi, A. V. Anisenkov, N. Anjos, A. Annovi, M. Antonelli, A. Antonov, J. Antos, F. Anulli, M. Aoki, L. Aperio Bella, G. Arabidze, Y. Arai, J. P. Araque, A. T. H. Arce, F. A. Arduh, J-F. Arguin, S. Argyropoulos, M. Arik, A. J. Armbruster, O. Arnaez, V. Arnal, H. Arnold, M. Arratia, O. Arslan, A. Artamonov, G. Artoni, S. Asai, N. Asbah, A. Ashkenazi, B. Åsman, L. Asquith, K. Assamagan, R. Astalos, M. Atkinson, N. B. Atlay, K. Augsten, M. Aurousseau, G. Avolio, B. Axen, M. K. Ayoub, G. Azuelos, M. A. Baak, A. E. Baas, M. J. Baca, C. Bacci, H. Bachacou, K. Bachas, M. Backes, M. Backhaus, P. Bagiacchi, P. Bagnaia, Y. Bai, T. Bain, J. T. Baines, O. K. Baker, E. M. Baldin, P. Balek, T. Balestri, F. Balli, E. Banas, Sw. Banerjee, A. A. E. Bannoura, H. S. Bansil, L. Barak, E. L. Barberio, D. Barberis, M. Barbero, T. Barillari, M. Barisonzi, T. Barklow, N. Barlow, S. L. Barnes, B. M. Barnett, R. M. Barnett, Z. Barnovska, A. Baroncelli, G. Barone, A. J. Barr, F. Barreiro, J. Barreiro Guimarães da Costa, R. Bartoldus, A. E. Barton, P. Bartos, A. Basalaev, A. Bassalat, A. Basye, R. L. Bates, S. J. Batista, J. R. Batley, M. Battaglia, M. Bauce, F. Bauer, H. S. Bawa, J. B. Beacham, M. D. Beattie, T. Beau, P. H. Beauchemin, R. Beccherle, P. Bechtle, H. P. Beck, K. Becker, M. Becker, S. Becker, M. Beckingham, C. Becot, A. J. Beddall, A. Beddall, V. A. Bednyakov, C. P. Bee, L. J. Beemster, T. A. Beermann, M. Begel, J. K. Behr, C. Belanger-Champagne, W. H. Bell, G. Bella, L. Bellagamba, A. Bellerive, M. Bellomo, K. Belotskiy, O. Beltramello, O. Benary, D. Benchekroun, M. Bender, K. Bendtz, N. Benekos, Y. Benhammou, E. Benhar Noccioli, J. A. Benitez Garcia, D. P. Benjamin, J. R. Bensinger, S. Bentvelsen, L. Beresford, M. Beretta, D. Berge, E. Bergeaas Kuutmann, N. Berger, F. Berghaus, J. Beringer, C. Bernard, N. R. Bernard, C. Bernius, F. U. Bernlochner, T. Berry, P. Berta, C. Bertella, G. Bertoli, F. Bertolucci, C. Bertsche, D. Bertsche, M. I. Besana, G. J. Besjes, O. Bessidskaia Bylund, M. Bessner, N. Besson, C. Betancourt, S. Bethke, A. J. Bevan, W. Bhimji, R. M. Bianchi, L. Bianchini, M. Bianco, O. Biebel, D. Biedermann, S. P. Bieniek, M. Biglietti, J. Bilbao De Mendizabal, H. Bilokon, M. Bindi, S. Binet, A. Bingul, C. Bini, S. Biondi, C. W. Black, J. E. Black, K. M. Black, D. Blackburn, R. E. Blair, J.-B. Blanchard, J. E. Blanco, T. Blazek, I. Bloch, C. Blocker, W. Blum, U. Blumenschein, G. J. Bobbink, V. S. Bobrovnikov, S. S. Bocchetta, A. Bocci, C. Bock, M. Boehler, J. A. Bogaerts, D. Bogavac, A. G. Bogdanchikov, C. Bohm, V. Boisvert, T. Bold, V. Boldea, A. S. Boldyrev, M. Bomben, M. Bona, M. Boonekamp, A. Borisov, G. Borissov, S. Borroni, J. Bortfeldt, V. Bortolotto, K. Bos, D. Boscherini, M. Bosman, J. Boudreau, J. Bouffard, E. V. Bouhova-Thacker, D. Boumediene, C. Bourdarios, N. Bousson, A. Boveia, J. Boyd, I. R. Boyko, I. Bozic, J. Bracinik, A. Brandt, G. Brandt, O. Brandt, U. Bratzler, B. Brau, J. E. Brau, H. M. Braun, S. F. Brazzale, W. D. Breaden Madden, K. Brendlinger, A. J. Brennan, L. Brenner, R. Brenner, S. Bressler, K. Bristow, T. M. Bristow, D. Britton, D. Britzger, F. M. Brochu, I. Brock, R. Brock, J. Bronner, G. Brooijmans, T. Brooks, W. K. Brooks, J. Brosamer, E. Brost, J. Brown, P. A. Bruckman de Renstrom, D. Bruncko, R. Bruneliere, A. Bruni, G. Bruni, M. Bruschi, N. Bruscino, L. Bryngemark, T. Buanes, Q. Buat, P. Buchholz, A. G. Buckley, S. I. Buda, I. A. Budagov, F. Buehrer, L. Bugge, M. K. Bugge, O. Bulekov, D. Bullock, H. Burckhart, S. Burdin, C. D. Burgard, B. Burghgrave, S. Burke, I. Burmeister, E. Busato, D. Büscher, V. Büscher, P. Bussey, J. M. Butler, A. I. Butt, C. M. Buttar, J. M. Butterworth, P. Butti, W. Buttinger, A. Buzatu, A. R. Buzykaev, S. Cabrera Urbán, D. Caforio, V. M. Cairo, O. Cakir, N. Calace, P. Calafiura, A. Calandri, G. Calderini, P. Calfayan, L. P. Caloba, D. Calvet, S. Calvet, R. Camacho Toro, S. Camarda, P. Camarri, D. Cameron, R. Caminal Armadans, S. Campana, M. Campanelli, A. Campoverde, V. Canale, A. Canepa, M. Cano Bret, J. Cantero, R. Cantrill, T. Cao, M. D. M. Capeans Garrido, I. Caprini, M. Caprini, M. Capua, R. Caputo, R. Cardarelli, F. Cardillo, T. Carli, G. Carlino, L. Carminati, S. Caron, E. Carquin, G. D. Carrillo-Montoya, J. R. Carter, J. Carvalho, D. Casadei, M. P. Casado, M. Casolino, E. Castaneda-Miranda, A. Castelli, V. Castillo Gimenez, N. F. Castro, P. Catastini, A. Catinaccio, J. R. Catmore, A. Cattai, J. Caudron, V. Cavaliere, D. Cavalli, M. Cavalli-Sforza, V. Cavasinni, F. Ceradini, B. C. Cerio, K. Cerny, A. S. Cerqueira, A. Cerri, L. Cerrito, F. Cerutti, M. Cerv, A. Cervelli, S. A. Cetin, A. Chafaq, D. Chakraborty, I. Chalupkova, P. Chang, J. D. Chapman, D. G. Charlton, C. C. Chau, C. A. Chavez Barajas, S. Cheatham, A. Chegwidden, S. Chekanov, S. V. Chekulaev, G. A. Chelkov, M. A. Chelstowska, C. Chen, H. Chen, K. Chen, L. Chen, S. Chen, X. Chen, Y. Chen, H. C. Cheng, Y. Cheng, A. Cheplakov, E. Cheremushkina, R. Cherkaoui El Moursli, V. Chernyatin, E. Cheu, L. Chevalier, V. Chiarella, G. Chiarelli, G. Chiodini, A. S. Chisholm, R. T. Chislett, A. Chitan, M. V. Chizhov, K. Choi, S. Chouridou, B. K. B. Chow, V. Christodoulou, D. Chromek-Burckhart, J. Chudoba, A. J. Chuinard, J. J. Chwastowski, L. Chytka, G. Ciapetti, A. K. Ciftci, D. Cinca, V. Cindro, I. A. Cioara, A. Ciocio, F. Cirotto, Z. H. Citron, M. Ciubancan, A. Clark, B. L. Clark, P. J. Clark, R. N. Clarke, W. Cleland, C. Clement, Y. Coadou, M. Cobal, A. Coccaro, J. Cochran, L. Coffey, J. G. Cogan, L. Colasurdo, B. Cole, S. Cole, A. P. Colijn, J. Collot, T. Colombo, G. Compostella, P. Conde Muiño, E. Coniavitis, S. H. Connell, I. A. Connelly, V. Consorti, S. Constantinescu, C. Conta, G. Conti, F. Conventi, M. Cooke, B. D. Cooper, A. M. Cooper-Sarkar, T. Cornelissen, M. Corradi, F. Corriveau, A. Corso-Radu, A. Cortes-Gonzalez, G. Cortiana, G. Costa, M. J. Costa, D. Costanzo, D. Côté, G. Cottin, G. Cowan, B. E. Cox, K. Cranmer, G. Cree, S. Crépé-Renaudin, F. Crescioli, W. A. Cribbs, M. Crispin Ortuzar, M. Cristinziani, V. Croft, G. Crosetti, T. Cuhadar Donszelmann, J. Cummings, M. Curatolo, C. Cuthbert, H. Czirr, P. Czodrowski, S. D’Auria, M. D’Onofrio, M. J. Da Cunha Sargedas De Sousa, C. Da Via, W. Dabrowski, A. Dafinca, T. Dai, O. Dale, F. Dallaire, C. Dallapiccola, M. Dam, J. R. Dandoy, N. P. Dang, A. C. Daniells, M. Danninger, M. Dano Hoffmann, V. Dao, G. Darbo, S. Darmora, J. Dassoulas, A. Dattagupta, W. Davey, C. David, T. Davidek, E. Davies, M. Davies, P. Davison, Y. Davygora, E. Dawe, I. Dawson, R. K. Daya-Ishmukhametova, K. De, R. de Asmundis, A. De Benedetti, S. De Castro, S. De Cecco, N. De Groot, P. de Jong, H. De la Torre, F. De Lorenzi, D. De Pedis, A. De Salvo, U. De Sanctis, A. De Santo, J. B. De Vivie De Regie, W. J. Dearnaley, R. Debbe, C. Debenedetti, D. V. Dedovich, I. Deigaard, J. Del Peso, T. Del Prete, D. Delgove, F. Deliot, C. M. Delitzsch, M. Deliyergiyev, A. Dell’Acqua, L. Dell’Asta, M. Dell’Orso, M. Della Pietra, D. della Volpe, M. Delmastro, P. A. Delsart, C. Deluca, D. A. DeMarco, S. Demers, M. Demichev, A. Demilly, S. P. Denisov, D. Derendarz, J. E. Derkaoui, F. Derue, P. Dervan, K. Desch, C. Deterre, P. O. Deviveiros, A. Dewhurst, S. Dhaliwal, A. Di Ciaccio, L. Di Ciaccio, A. Di Domenico, C. Di Donato, A. Di Girolamo, B. Di Girolamo, A. Di Mattia, B. Di Micco, R. Di Nardo, A. Di Simone, R. Di Sipio, D. Di Valentino, C. Diaconu, M. Diamond, F. A. Dias, M. A. Diaz, E. B. Diehl, J. Dietrich, S. Diglio, A. Dimitrievska, J. Dingfelder, P. Dita, S. Dita, F. Dittus, F. Djama, T. Djobava, J. I. Djuvsland, M. A. B. do Vale, D. Dobos, M. Dobre, C. Doglioni, T. Dohmae, J. Dolejsi, Z. Dolezal, B. A. Dolgoshein, M. Donadelli, S. Donati, P. Dondero, J. Donini, J. Dopke, A. Doria, M. T. Dova, A. T. Doyle, E. Drechsler, M. Dris, E. Dubreuil, E. Duchovni, G. Duckeck, O. A. Ducu, D. Duda, A. Dudarev, L. Duflot, L. Duguid, M. Dührssen, M. Dunford, H. Duran Yildiz, M. Düren, A. Durglishvili, D. Duschinger, M. Dyndal, C. Eckardt, K. M. Ecker, R. C. Edgar, W. Edson, N. C. Edwards, W. Ehrenfeld, T. Eifert, G. Eigen, K. Einsweiler, T. Ekelof, M. El Kacimi, M. Ellert, S. Elles, F. Ellinghaus, A. A. Elliot, N. Ellis, J. Elmsheuser, M. Elsing, D. Emeliyanov, Y. Enari, O. C. Endner, M. Endo, J. Erdmann, A. Ereditato, G. Ernis, J. Ernst, M. Ernst, S. Errede, E. Ertel, M. Escalier, H. Esch, C. Escobar, B. Esposito, A. I. Etienvre, E. Etzion, H. Evans, A. Ezhilov, L. Fabbri, G. Facini, R. M. Fakhrutdinov, S. Falciano, R. J. Falla, J. Faltova, Y. Fang, M. Fanti, A. Farbin, A. Farilla, T. Farooque, S. Farrell, S. M. Farrington, P. Farthouat, F. Fassi, P. Fassnacht, D. Fassouliotis, M. Faucci Giannelli, A. Favareto, L. Fayard, P. Federic, O. L. Fedin, W. Fedorko, S. Feigl, L. Feligioni, C. Feng, E. J. Feng, H. Feng, A. B. Fenyuk, L. Feremenga, P. Fernandez Martinez, S. Fernandez Perez, J. Ferrando, A. Ferrari, P. Ferrari, R. Ferrari, D. E. Ferreira de Lima, A. Ferrer, D. Ferrere, C. Ferretti, A. Ferretto Parodi, M. Fiascaris, F. Fiedler, A. Filipčič, M. Filipuzzi, F. Filthaut, M. Fincke-Keeler, K. D. Finelli, M. C. N. Fiolhais, L. Fiorini, A. Firan, A. Fischer, C. Fischer, J. Fischer, W. C. Fisher, E. A. Fitzgerald, N. Flaschel, I. Fleck, P. Fleischmann, S. Fleischmann, G. T. Fletcher, G. Fletcher, R. R. M. Fletcher, T. Flick, A. Floderus, L. R. Flores Castillo, M. J. Flowerdew, A. Formica, A. Forti, D. Fournier, H. Fox, S. Fracchia, P. Francavilla, M. Franchini, D. Francis, L. Franconi, M. Franklin, M. Frate, M. Fraternali, D. Freeborn, S. T. French, F. Friedrich, D. Froidevaux, J. A. Frost, C. Fukunaga, E. Fullana Torregrosa, B. G. Fulsom, T. Fusayasu, J. Fuster, C. Gabaldon, O. Gabizon, A. Gabrielli, A. Gabrielli, G. P. Gach, S. Gadatsch, S. Gadomski, G. Gagliardi, P. Gagnon, C. Galea, B. Galhardo, E. J. Gallas, B. J. Gallop, P. Gallus, G. Galster, K. K. Gan, J. Gao, Y. Gao, Y. S. Gao, F. M. Garay Walls, F. Garberson, C. García, J. E. García Navarro, M. Garcia-Sciveres, R. W. Gardner, N. Garelli, V. Garonne, C. Gatti, A. Gaudiello, G. Gaudio, B. Gaur, L. Gauthier, P. Gauzzi, I. L. Gavrilenko, C. Gay, G. Gaycken, E. N. Gazis, P. Ge, Z. Gecse, C. N. P. Gee, Ch. Geich-Gimbel, M. P. Geisler, C. Gemme, M. H. Genest, S. Gentile, M. George, S. George, D. Gerbaudo, A. Gershon, S. Ghasemi, H. Ghazlane, B. Giacobbe, S. Giagu, V. Giangiobbe, P. Giannetti, B. Gibbard, S. M. Gibson, M. Gilchriese, T. P. S. Gillam, D. Gillberg, G. Gilles, D. M. Gingrich, N. Giokaris, M. P. Giordani, F. M. Giorgi, F. M. Giorgi, P. F. Giraud, P. Giromini, D. Giugni, C. Giuliani, M. Giulini, B. K. Gjelsten, S. Gkaitatzis, I. Gkialas, E. L. Gkougkousis, L. K. Gladilin, C. Glasman, J. Glatzer, P. C. F. Glaysher, A. Glazov, M. Goblirsch-Kolb, J. R. Goddard, J. Godlewski, S. Goldfarb, T. Golling, D. Golubkov, A. Gomes, R. Gonçalo, J. Goncalves Pinto Firmino Da Costa, L. Gonella, S. González de la Hoz, G. Gonzalez Parra, S. Gonzalez-Sevilla, L. Goossens, P. A. Gorbounov, H. A. Gordon, I. Gorelov, B. Gorini, E. Gorini, A. Gorišek, E. Gornicki, A. T. Goshaw, C. Gössling, M. I. Gostkin, D. Goujdami, A. G. Goussiou, N. Govender, E. Gozani, H. M. X. Grabas, L. Graber, I. Grabowska-Bold, P. O. J. Gradin, P. Grafström, K-J. Grahn, J. Gramling, E. Gramstad, S. Grancagnolo, V. Gratchev, H. M. Gray, E. Graziani, Z. D. Greenwood, K. Gregersen, I. M. Gregor, P. Grenier, J. Griffiths, A. A. Grillo, K. Grimm, S. Grinstein, Ph. Gris, J.-F. Grivaz, J. P. Grohs, A. Grohsjean, E. Gross, J. Grosse-Knetter, G. C. Grossi, Z. J. Grout, L. Guan, J. Guenther, F. Guescini, D. Guest, O. Gueta, E. Guido, T. Guillemin, S. Guindon, U. Gul, C. Gumpert, J. Guo, Y. Guo, S. Gupta, G. Gustavino, P. Gutierrez, N. G. Gutierrez Ortiz, C. Gutschow, C. Guyot, C. Gwenlan, C. B. Gwilliam, A. Haas, C. Haber, H. K. Hadavand, N. Haddad, P. Haefner, S. Hageböck, Z. Hajduk, H. Hakobyan, M. Haleem, J. Haley, D. Hall, G. Halladjian, G. D. Hallewell, K. Hamacher, P. Hamal, K. Hamano, A. Hamilton, G. N. Hamity, P. G. Hamnett, L. Han, K. Hanagaki, K. Hanawa, M. Hance, P. Hanke, R. Hanna, J. B. Hansen, J. D. Hansen, M. C. Hansen, P. H. Hansen, K. Hara, A. S. Hard, T. Harenberg, F. Hariri, S. Harkusha, R. D. Harrington, P. F. Harrison, F. Hartjes, M. Hasegawa, Y. Hasegawa, A. Hasib, S. Hassani, S. Haug, R. Hauser, L. Hauswald, M. Havranek, C. M. Hawkes, R. J. Hawkings, A. D. Hawkins, T. Hayashi, D. Hayden, C. P. Hays, J. M. Hays, H. S. Hayward, S. J. Haywood, S. J. Head, T. Heck, V. Hedberg, L. Heelan, S. Heim, T. Heim, B. Heinemann, L. Heinrich, J. Hejbal, L. Helary, S. Hellman, D. Hellmich, C. Helsens, J. Henderson, R. C. W. Henderson, Y. Heng, C. Hengler, A. Henrichs, A. M. Henriques Correia, S. Henrot-Versille, G. H. Herbert, Y. Hernández Jiménez, R. Herrberg-Schubert, G. Herten, R. Hertenberger, L. Hervas, G. G. Hesketh, N. P. Hessey, J. W. Hetherly, R. Hickling, E. Higón-Rodriguez, E. Hill, J. C. Hill, K. H. Hiller, S. J. Hillier, I. Hinchliffe, E. Hines, R. R. Hinman, M. Hirose, D. Hirschbuehl, J. Hobbs, N. Hod, M. C. Hodgkinson, P. Hodgson, A. Hoecker, M. R. Hoeferkamp, F. Hoenig, M. Hohlfeld, D. Hohn, T. R. Holmes, M. Homann, T. M. Hong, L. Hooft van Huysduynen, W. H. Hopkins, Y. Horii, A. J. Horton, J-Y. Hostachy, S. Hou, A. Hoummada, J. Howard, J. Howarth, M. Hrabovsky, I. Hristova, J. Hrivnac, T. Hryn’ova, A. Hrynevich, C. Hsu, P. J. Hsu, S.-C. Hsu, D. Hu, Q. Hu, X. Hu, Y. Huang, Z. Hubacek, F. Hubaut, F. Huegging, T. B. Huffman, E. W. Hughes, G. Hughes, M. Huhtinen, T. A. Hülsing, N. Huseynov, J. Huston, J. Huth, G. Iacobucci, G. Iakovidis, I. Ibragimov, L. Iconomidou-Fayard, E. Ideal, Z. Idrissi, P. Iengo, O. Igonkina, T. Iizawa, Y. Ikegami, K. Ikematsu, M. Ikeno, Y. Ilchenko, D. Iliadis, N. Ilic, T. Ince, G. Introzzi, P. Ioannou, M. Iodice, K. Iordanidou, V. Ippolito, A. Irles Quiles, C. Isaksson, M. Ishino, M. Ishitsuka, R. Ishmukhametov, C. Issever, S. Istin, J. M. Iturbe Ponce, R. Iuppa, J. Ivarsson, W. Iwanski, H. Iwasaki, J. M. Izen, V. Izzo, S. Jabbar, B. Jackson, M. Jackson, P. Jackson, M. R. Jaekel, V. Jain, K. Jakobs, S. Jakobsen, T. Jakoubek, J. Jakubek, D. O. Jamin, D. K. Jana, E. Jansen, R. Jansky, J. Janssen, M. Janus, G. Jarlskog, N. Javadov, T. Javůrek, L. Jeanty, J. Jejelava, G.-Y. Jeng, D. Jennens, P. Jenni, J. Jentzsch, C. Jeske, S. Jézéquel, H. Ji, J. Jia, Y. Jiang, S. Jiggins, J. Jimenez Pena, S. Jin, A. Jinaru, O. Jinnouchi, M. D. Joergensen, P. Johansson, K. A. Johns, K. Jon-And, G. Jones, R. W. L. Jones, T. J. Jones, J. Jongmanns, P. M. Jorge, K. D. Joshi, J. Jovicevic, X. Ju, C. A. Jung, P. Jussel, A. Juste Rozas, M. Kaci, A. Kaczmarska, M. Kado, H. Kagan, M. Kagan, S. J. Kahn, E. Kajomovitz, C. W. Kalderon, S. Kama, A. Kamenshchikov, N. Kanaya, S. Kaneti, V. A. Kantserov, J. Kanzaki, B. Kaplan, L. S. Kaplan, A. Kapliy, D. Kar, K. Karakostas, A. Karamaoun, N. Karastathis, M. J. Kareem, E. Karentzos, M. Karnevskiy, S. N. Karpov, Z. M. Karpova, K. Karthik, V. Kartvelishvili, A. N. Karyukhin, L. Kashif, R. D. Kass, A. Kastanas, Y. Kataoka, C. Kato, A. Katre, J. Katzy, K. Kawagoe, T. Kawamoto, G. Kawamura, S. Kazama, V. F. Kazanin, R. Keeler, R. Kehoe, J. S. Keller, J. J. Kempster, H. Keoshkerian, O. Kepka, B. P. Kerševan, S. Kersten, R. A. Keyes, F. Khalil-zada, H. Khandanyan, A. Khanov, A. G. Kharlamov, T. J. Khoo, V. Khovanskiy, E. Khramov, J. Khubua, S. Kido, H. Y. Kim, S. H. Kim, Y. K. Kim, N. Kimura, O. M. Kind, B. T. King, M. King, S. B. King, J. Kirk, A. E. Kiryunin, T. Kishimoto, D. Kisielewska, F. Kiss, K. Kiuchi, O. Kivernyk, E. Kladiva, M. H. Klein, M. Klein, U. Klein, K. Kleinknecht, P. Klimek, A. Klimentov, R. Klingenberg, J. A. Klinger, T. Klioutchnikova, E.-E. Kluge, P. Kluit, S. Kluth, J. Knapik, E. Kneringer, E. B. F. G. Knoops, A. Knue, A. Kobayashi, D. Kobayashi, T. Kobayashi, M. Kobel, M. Kocian, P. Kodys, T. Koffas, E. Koffeman, L. A. Kogan, S. Kohlmann, Z. Kohout, T. Kohriki, T. Koi, H. Kolanoski, I. Koletsou, A. A. Komar, Y. Komori, T. Kondo, N. Kondrashova, K. Köneke, A. C. König, T. Kono, R. Konoplich, N. Konstantinidis, R. Kopeliansky, S. Koperny, L. Köpke, A. K. Kopp, K. Korcyl, K. Kordas, A. Korn, A. A. Korol, I. Korolkov, E. V. Korolkova, O. Kortner, S. Kortner, T. Kosek, V. V. Kostyukhin, V. M. Kotov, A. Kotwal, A. Kourkoumeli-Charalampidi, C. Kourkoumelis, V. Kouskoura, A. Koutsman, R. Kowalewski, T. Z. Kowalski, W. Kozanecki, A. S. Kozhin, V. A. Kramarenko, G. Kramberger, D. Krasnopevtsev, M. W. Krasny, A. Krasznahorkay, J. K. Kraus, A. Kravchenko, S. Kreiss, M. Kretz, J. Kretzschmar, K. Kreutzfeldt, P. Krieger, K. Krizka, K. Kroeninger, H. Kroha, J. Kroll, J. Kroseberg, J. Krstic, U. Kruchonak, H. Krüger, N. Krumnack, A. Kruse, M. C. Kruse, M. Kruskal, T. Kubota, H. Kucuk, S. Kuday, S. Kuehn, A. Kugel, F. Kuger, A. Kuhl, T. Kuhl, V. Kukhtin, Y. Kulchitsky, S. Kuleshov, M. Kuna, T. Kunigo, A. Kupco, H. Kurashige, Y. A. Kurochkin, V. Kus, E. S. Kuwertz, M. Kuze, J. Kvita, T. Kwan, D. Kyriazopoulos, A. La Rosa, J. L. La Rosa Navarro, L. La Rotonda, C. Lacasta, F. Lacava, J. Lacey, H. Lacker, D. Lacour, V. R. Lacuesta, E. Ladygin, R. Lafaye, B. Laforge, T. Lagouri, S. Lai, L. Lambourne, S. Lammers, C. L. Lampen, W. Lampl, E. Lançon, U. Landgraf, M. P. J. Landon, V. S. Lang, J. C. Lange, A. J. Lankford, F. Lanni, K. Lantzsch, A. Lanza, S. Laplace, C. Lapoire, J. F. Laporte, T. Lari, F. Lasagni Manghi, M. Lassnig, P. Laurelli, W. Lavrijsen, A. T. Law, P. Laycock, T. Lazovich, O. Le Dortz, E. Le Guirriec, E. Le Menedeu, M. LeBlanc, T. LeCompte, F. Ledroit-Guillon, C. A. Lee, S. C. Lee, L. Lee, G. Lefebvre, M. Lefebvre, F. Legger, C. Leggett, A. Lehan, G. Lehmann Miotto, X. Lei, W. A. Leight, A. Leisos, A. G. Leister, M. A. L. Leite, R. Leitner, D. Lellouch, B. Lemmer, K. J. C. Leney, T. Lenz, B. Lenzi, R. Leone, S. Leone, C. Leonidopoulos, S. Leontsinis, C. Leroy, C. G. Lester, M. Levchenko, J. Levêque, D. Levin, L. J. Levinson, M. Levy, A. Lewis, A. M. Leyko, M. Leyton, B. Li, H. Li, H. L. Li, L. Li, L. Li, S. Li, X. Li, Y. Li, Z. Liang, H. Liao, B. Liberti, A. Liblong, P. Lichard, K. Lie, J. Liebal, W. Liebig, C. Limbach, A. Limosani, S. C. Lin, T. H. Lin, F. Linde, B. E. Lindquist, J. T. Linnemann, E. Lipeles, A. Lipniacka, M. Lisovyi, T. M. Liss, D. Lissauer, A. Lister, A. M. Litke, B. Liu, D. Liu, H. Liu, J. Liu, J. B. Liu, K. Liu, L. Liu, M. Liu, M. Liu, Y. Liu, M. Livan, A. Lleres, J. Llorente Merino, S. L. Lloyd, F. Lo Sterzo, E. Lobodzinska, P. Loch, W. S. Lockman, F. K. Loebinger, A. E. Loevschall-Jensen, A. Loginov, T. Lohse, K. Lohwasser, M. Lokajicek, B. A. Long, J. D. Long, R. E. Long, K. A. Looper, L. Lopes, D. Lopez Mateos, B. Lopez Paredes, I. Lopez Paz, J. Lorenz, N. Lorenzo Martinez, M. Losada, P. Loscutoff, P. J. Lösel, X. Lou, A. Lounis, J. Love, P. A. Love, N. Lu, H. J. Lubatti, C. Luci, A. Lucotte, F. Luehring, W. Lukas, L. Luminari, O. Lundberg, B. Lund-Jensen, D. Lynn, R. Lysak, E. Lytken, H. Ma, L. L. Ma, G. Maccarrone, A. Macchiolo, C. M. Macdonald, B. Maček, J. Machado Miguens, D. Macina, D. Madaffari, R. Madar, H. J. Maddocks, W. F. Mader, A. Madsen, J. Maeda, S. Maeland, T. Maeno, A. Maevskiy, E. Magradze, K. Mahboubi, J. Mahlstedt, C. Maiani, C. Maidantchik, A. A. Maier, T. Maier, A. Maio, S. Majewski, Y. Makida, N. Makovec, B. Malaescu, Pa. Malecki, V. P. Maleev, F. Malek, U. Mallik, D. Malon, C. Malone, S. Maltezos, V. M. Malyshev, S. Malyukov, J. Mamuzic, G. Mancini, B. Mandelli, L. Mandelli, I. Mandić, R. Mandrysch, J. Maneira, A. Manfredini, L. Manhaes de Andrade Filho, J. Manjarres Ramos, A. Mann, A. Manousakis-Katsikakis, B. Mansoulie, R. Mantifel, M. Mantoani, L. Mapelli, L. March, G. Marchiori, M. Marcisovsky, C. P. Marino, M. Marjanovic, D. E. Marley, F. Marroquim, S. P. Marsden, Z. Marshall, L. F. Marti, S. Marti-Garcia, B. Martin, T. A. Martin, V. J. Martin, B. Martin dit Latour, M. Martinez, S. Martin-Haugh, V. S. Martoiu, A. C. Martyniuk, M. Marx, F. Marzano, A. Marzin, L. Masetti, T. Mashimo, R. Mashinistov, J. Masik, A. L. Maslennikov, I. Massa, L. Massa, N. Massol, P. Mastrandrea, A. Mastroberardino, T. Masubuchi, P. Mättig, J. Mattmann, J. Maurer, S. J. Maxfield, D. A. Maximov, R. Mazini, S. M. Mazza, L. Mazzaferro, G. Mc Goldrick, S. P. Mc Kee, A. McCarn, R. L. McCarthy, T. G. McCarthy, N. A. McCubbin, K. W. McFarlane, J. A. Mcfayden, G. Mchedlidze, S. J. McMahon, R. A. McPherson, M. Medinnis, S. Meehan, S. Mehlhase, A. Mehta, K. Meier, C. Meineck, B. Meirose, B. R. Mellado Garcia, F. Meloni, A. Mengarelli, S. Menke, E. Meoni, K. M. Mercurio, S. Mergelmeyer, P. Mermod, L. Merola, C. Meroni, F. S. Merritt, A. Messina, J. Metcalfe, A. S. Mete, C. Meyer, C. Meyer, J-P. Meyer, J. Meyer, H. Meyer Zu Theenhausen, R. P. Middleton, S. Miglioranzi, L. Mijović, G. Mikenberg, M. Mikestikova, M. Mikuž, M. Milesi, A. Milic, D. W. Miller, C. Mills, A. Milov, D. A. Milstead, A. A. Minaenko, Y. Minami, I. A. Minashvili, A. I. Mincer, B. Mindur, M. Mineev, Y. Ming, L. M. Mir, T. Mitani, J. Mitrevski, V. A. Mitsou, A. Miucci, P. S. Miyagawa, J. U. Mjörnmark, T. Moa, K. Mochizuki, S. Mohapatra, W. Mohr, S. Molander, R. Moles-Valls, K. Mönig, C. Monini, J. Monk, E. Monnier, J. Montejo Berlingen, F. Monticelli, S. Monzani, R. W. Moore, N. Morange, D. Moreno, M. Moreno Llácer, P. Morettini, D. Mori, M. Morii, M. Morinaga, V. Morisbak, S. Moritz, A. K. Morley, G. Mornacchi, J. D. Morris, S. S. Mortensen, A. Morton, L. Morvaj, M. Mosidze, J. Moss, K. Motohashi, R. Mount, E. Mountricha, S. V. Mouraviev, E. J. W. Moyse, S. Muanza, R. D. Mudd, F. Mueller, J. Mueller, R. S. P. Mueller, T. Mueller, D. Muenstermann, P. Mullen, G. A. Mullier, J. A. Murillo Quijada, W. J. Murray, H. Musheghyan, E. Musto, A. G. Myagkov, M. Myska, B. P. Nachman, O. Nackenhorst, J. Nadal, K. Nagai, R. Nagai, Y. Nagai, K. Nagano, A. Nagarkar, Y. Nagasaka, K. Nagata, M. Nagel, E. Nagy, A. M. Nairz, Y. Nakahama, K. Nakamura, T. Nakamura, I. Nakano, H. Namasivayam, R. F. Naranjo Garcia, R. Narayan, D. I. Narrias Villar, T. Naumann, G. Navarro, R. Nayyar, H. A. Neal, P. Yu. Nechaeva, T. J. Neep, P. D. Nef, A. Negri, M. Negrini, S. Nektarijevic, C. Nellist, A. Nelson, S. Nemecek, P. Nemethy, A. A. Nepomuceno, M. Nessi, M. S. Neubauer, M. Neumann, R. M. Neves, P. Nevski, P. R. Newman, D. H. Nguyen, R. B. Nickerson, R. Nicolaidou, B. Nicquevert, J. Nielsen, N. Nikiforou, A. Nikiforov, V. Nikolaenko, I. Nikolic-Audit, K. Nikolopoulos, J. K. Nilsen, P. Nilsson, Y. Ninomiya, A. Nisati, R. Nisius, T. Nobe, M. Nomachi, I. Nomidis, T. Nooney, S. Norberg, M. Nordberg, O. Novgorodova, S. Nowak, M. Nozaki, L. Nozka, K. Ntekas, G. Nunes Hanninger, T. Nunnemann, E. Nurse, F. Nuti, B. J. O’Brien, F. O’grady, D. C. O’Neil, V. O’Shea, F. G. Oakham, H. Oberlack, T. Obermann, J. Ocariz, A. Ochi, I. Ochoa, J. P. Ochoa-Ricoux, S. Oda, S. Odaka, H. Ogren, A. Oh, S. H. Oh, C. C. Ohm, H. Ohman, H. Oide, W. Okamura, H. Okawa, Y. Okumura, T. Okuyama, A. Olariu, S. A. Olivares Pino, D. Oliveira Damazio, E. Oliver Garcia, A. Olszewski, J. Olszowska, A. Onofre, P. U. E. Onyisi, C. J. Oram, M. J. Oreglia, Y. Oren, D. Orestano, N. Orlando, C. Oropeza Barrera, R. S. Orr, B. Osculati, R. Ospanov, G. Otero y Garzon, H. Otono, M. Ouchrif, F. Ould-Saada, A. Ouraou, K. P. Oussoren, Q. Ouyang, A. Ovcharova, M. Owen, R. E. Owen, V. E. Ozcan, N. Ozturk, K. Pachal, A. Pacheco Pages, C. Padilla Aranda, M. Pagáčová, S. Pagan Griso, E. Paganis, F. Paige, P. Pais, K. Pajchel, G. Palacino, S. Palestini, M. Palka, D. Pallin, A. Palma, Y. B. Pan, E. Panagiotopoulou, C. E. Pandini, J. G. Panduro Vazquez, P. Pani, S. Panitkin, D. Pantea, L. Paolozzi, Th. D. Papadopoulou, K. Papageorgiou, A. Paramonov, D. Paredes Hernandez, M. A. Parker, K. A. Parker, F. Parodi, J. A. Parsons, U. Parzefall, E. Pasqualucci, S. Passaggio, F. Pastore, Fr. Pastore, G. Pásztor, S. Pataraia, N. D. Patel, J. R. Pater, T. Pauly, J. Pearce, B. Pearson, L. E. Pedersen, M. Pedersen, S. Pedraza Lopez, R. Pedro, S. V. Peleganchuk, D. Pelikan, O. Penc, C. Peng, H. Peng, B. Penning, J. Penwell, D. V. Perepelitsa, E. Perez Codina, M. T. Pérez García-Estañ, L. Perini, H. Pernegger, S. Perrella, R. Peschke, V. D. Peshekhonov, K. Peters, R. F. Y. Peters, B. A. Petersen, T. C. Petersen, E. Petit, A. Petridis, C. Petridou, P. Petroff, E. Petrolo, F. Petrucci, N. E. Pettersson, R. Pezoa, P. W. Phillips, G. Piacquadio, E. Pianori, A. Picazio, E. Piccaro, M. Piccinini, M. A. Pickering, R. Piegaia, D. T. Pignotti, J. E. Pilcher, A. D. Pilkington, J. Pina, M. Pinamonti, J. L. Pinfold, A. Pingel, S. Pires, H. Pirumov, M. Pitt, C. Pizio, L. Plazak, M.-A. Pleier, V. Pleskot, E. Plotnikova, P. Plucinski, D. Pluth, R. Poettgen, L. Poggioli, D. Pohl, G. Polesello, A. Poley, A. Policicchio, R. Polifka, A. Polini, C. S. Pollard, V. Polychronakos, K. Pommès, L. Pontecorvo, B. G. Pope, G. A. Popeneciu, D. S. Popovic, A. Poppleton, S. Pospisil, K. Potamianos, I. N. Potrap, C. J. Potter, C. T. Potter, G. Poulard, J. Poveda, V. Pozdnyakov, P. Pralavorio, A. Pranko, S. Prasad, S. Prell, D. Price, L. E. Price, M. Primavera, S. Prince, M. Proissl, K. Prokofiev, F. Prokoshin, E. Protopapadaki, S. Protopopescu, J. Proudfoot, M. Przybycien, E. Ptacek, D. Puddu, E. Pueschel, D. Puldon, M. Purohit, P. Puzo, J. Qian, G. Qin, Y. Qin, A. Quadt, D. R. Quarrie, W. B. Quayle, M. Queitsch-Maitland, D. Quilty, S. Raddum, V. Radeka, V. Radescu, S. K. Radhakrishnan, P. Radloff, P. Rados, F. Ragusa, G. Rahal, S. Rajagopalan, M. Rammensee, C. Rangel-Smith, F. Rauscher, S. Rave, T. Ravenscroft, M. Raymond, A. L. Read, N. P. Readioff, D. M. Rebuzzi, A. Redelbach, G. Redlinger, R. Reece, K. Reeves, L. Rehnisch, J. Reichert, H. Reisin, M. Relich, C. Rembser, H. Ren, A. Renaud, M. Rescigno, S. Resconi, O. L. Rezanova, P. Reznicek, R. Rezvani, R. Richter, S. Richter, E. Richter-Was, O. Ricken, M. Ridel, P. Rieck, C. J. Riegel, J. Rieger, M. Rijssenbeek, A. Rimoldi, L. Rinaldi, B. Ristić, E. Ritsch, I. Riu, F. Rizatdinova, E. Rizvi, S. H. Robertson, A. Robichaud-Veronneau, D. Robinson, J. E. M. Robinson, A. Robson, C. Roda, S. Roe, O. Røhne, S. Rolli, A. Romaniouk, M. Romano, S. M. Romano Saez, E. Romero Adam, N. Rompotis, M. Ronzani, L. Roos, E. Ros, S. Rosati, K. Rosbach, P. Rose, P. L. Rosendahl, O. Rosenthal, V. Rossetti, E. Rossi, L. P. Rossi, J. H. N. Rosten, R. Rosten, M. Rotaru, I. Roth, J. Rothberg, D. Rousseau, C. R. Royon, A. Rozanov, Y. Rozen, X. Ruan, F. Rubbo, I. Rubinskiy, V. I. Rud, C. Rudolph, M. S. Rudolph, F. Rühr, A. Ruiz-Martinez, Z. Rurikova, N. A. Rusakovich, A. Ruschke, H. L. Russell, J. P. Rutherfoord, N. Ruthmann, Y. F. Ryabov, M. Rybar, G. Rybkin, N. C. Ryder, A. F. Saavedra, G. Sabato, S. Sacerdoti, A. Saddique, H. F-W. Sadrozinski, R. Sadykov, F. Safai Tehrani, M. Sahinsoy, M. Saimpert, T. Saito, H. Sakamoto, Y. Sakurai, G. Salamanna, A. Salamon, J. E. Salazar Loyola, M. Saleem, D. Salek, P. H. Sales De Bruin, D. Salihagic, A. Salnikov, J. Salt, D. Salvatore, F. Salvatore, A. Salvucci, A. Salzburger, D. Sammel, D. Sampsonidis, A. Sanchez, J. Sánchez, V. Sanchez Martinez, H. Sandaker, R. L. Sandbach, H. G. Sander, M. P. Sanders, M. Sandhoff, C. Sandoval, R. Sandstroem, D. P. C. Sankey, M. Sannino, A. Sansoni, C. Santoni, R. Santonico, H. Santos, I. Santoyo Castillo, K. Sapp, A. Sapronov, J. G. Saraiva, B. Sarrazin, O. Sasaki, Y. Sasaki, K. Sato, G. Sauvage, E. Sauvan, G. Savage, P. Savard, C. Sawyer, L. Sawyer, J. Saxon, C. Sbarra, A. Sbrizzi, T. Scanlon, D. A. Scannicchio, M. Scarcella, V. Scarfone, J. Schaarschmidt, P. Schacht, D. Schaefer, R. Schaefer, J. Schaeffer, S. Schaepe, S. Schaetzel, U. Schäfer, A. C. Schaffer, D. Schaile, R. D. Schamberger, V. Scharf, V. A. Schegelsky, D. Scheirich, M. Schernau, C. Schiavi, C. Schillo, M. Schioppa, S. Schlenker, K. Schmieden, C. Schmitt, S. Schmitt, S. Schmitt, B. Schneider, Y. J. Schnellbach, U. Schnoor, L. Schoeffel, A. Schoening, B. D. Schoenrock, E. Schopf, A. L. S. Schorlemmer, M. Schott, D. Schouten, J. Schovancova, S. Schramm, M. Schreyer, C. Schroeder, N. Schuh, M. J. Schultens, H.-C. Schultz-Coulon, H. Schulz, M. Schumacher, B. A. Schumm, Ph. Schune, C. Schwanenberger, A. Schwartzman, T. A. Schwarz, Ph. Schwegler, H. Schweiger, Ph. Schwemling, R. Schwienhorst, J. Schwindling, T. Schwindt, F. G. Sciacca, E. Scifo, G. Sciolla, F. Scuri, F. Scutti, J. Searcy, G. Sedov, E. Sedykh, P. Seema, S. C. Seidel, A. Seiden, F. Seifert, J. M. Seixas, G. Sekhniaidze, K. Sekhon, S. J. Sekula, D. M. Seliverstov, N. Semprini-Cesari, C. Serfon, L. Serin, L. Serkin, T. Serre, M. Sessa, R. Seuster, H. Severini, T. Sfiligoj, F. Sforza, A. Sfyrla, E. Shabalina, M. Shamim, L. Y. Shan, R. Shang, J. T. Shank, M. Shapiro, P. B. Shatalov, K. Shaw, S. M. Shaw, A. Shcherbakova, C. Y. Shehu, P. Sherwood, L. Shi, S. Shimizu, C. O. Shimmin, M. Shimojima, M. Shiyakova, A. Shmeleva, D. Shoaleh Saadi, M. J. Shochet, S. Shojaii, S. Shrestha, E. Shulga, M. A. Shupe, S. Shushkevich, P. Sicho, P. E. Sidebo, O. Sidiropoulou, D. Sidorov, A. Sidoti, F. Siegert, Dj. Sijacki, J. Silva, Y. Silver, S. B. Silverstein, V. Simak, O. Simard, Lj. Simic, S. Simion, E. Simioni, B. Simmons, D. Simon, P. Sinervo, N. B. Sinev, M. Sioli, G. Siragusa, A. N. Sisakyan, S. Yu. Sivoklokov, J. Sjölin, T. B. Sjursen, M. B. Skinner, H. P. Skottowe, P. Skubic, M. Slater, T. Slavicek, M. Slawinska, K. Sliwa, V. Smakhtin, B. H. Smart, L. Smestad, S. Yu. Smirnov, Y. Smirnov, L. N. Smirnova, O. Smirnova, M. N. K. Smith, R. W. Smith, M. Smizanska, K. Smolek, A. A. Snesarev, G. Snidero, S. Snyder, R. Sobie, F. Socher, A. Soffer, D. A. Soh, G. Sokhrannyi, C. A. Solans, M. Solar, J. Solc, E. Yu. Soldatov, U. Soldevila, A. A. Solodkov, A. Soloshenko, O. V. Solovyanov, V. Solovyev, P. Sommer, H. Y. Song, N. Soni, A. Sood, A. Sopczak, B. Sopko, V. Sopko, V. Sorin, D. Sosa, M. Sosebee, C. L. Sotiropoulou, R. Soualah, A. M. Soukharev, D. South, B. C. Sowden, S. Spagnolo, M. Spalla, M. Spangenberg, F. Spanò, W. R. Spearman, D. Sperlich, F. Spettel, R. Spighi, G. Spigo, L. A. Spiller, M. Spousta, T. Spreitzer, R. D. St. Denis, S. Staerz, J. Stahlman, R. Stamen, S. Stamm, E. Stanecka, C. Stanescu, M. Stanescu-Bellu, M. M. Stanitzki, S. Stapnes, E. A. Starchenko, J. Stark, P. Staroba, P. Starovoitov, R. Staszewski, P. Stavina, P. Steinberg, B. Stelzer, H. J. Stelzer, O. Stelzer-Chilton, H. Stenzel, G. A. Stewart, J. A. Stillings, M. C. Stockton, M. Stoebe, G. Stoicea, P. Stolte, S. Stonjek, A. R. Stradling, A. Straessner, M. E. Stramaglia, J. Strandberg, S. Strandberg, A. Strandlie, E. Strauss, M. Strauss, P. Strizenec, R. Ströhmer, D. M. Strom, R. Stroynowski, A. Strubig, S. A. Stucci, B. Stugu, N. A. Styles, D. Su, J. Su, R. Subramaniam, A. Succurro, Y. Sugaya, C. Suhr, M. Suk, V. V. Sulin, S. Sultansoy, T. Sumida, S. Sun, X. Sun, J. E. Sundermann, K. Suruliz, G. Susinno, M. R. Sutton, S. Suzuki, M. Svatos, M. Swiatlowski, I. Sykora, T. Sykora, D. Ta, C. Taccini, K. Tackmann, J. Taenzer, A. Taffard, R. Tafirout, N. Taiblum, H. Takai, R. Takashima, H. Takeda, T. Takeshita, Y. Takubo, M. Talby, A. A. Talyshev, J. Y. C. Tam, K. G. Tan, J. Tanaka, R. Tanaka, S. Tanaka, B. B. Tannenwald, N. Tannoury, S. Tapprogge, S. Tarem, F. Tarrade, G. F. Tartarelli, P. Tas, M. Tasevsky, T. Tashiro, E. Tassi, A. Tavares Delgado, Y. Tayalati, F. E. Taylor, G. N. Taylor, W. Taylor, F. A. Teischinger, M. Teixeira Dias Castanheira, P. Teixeira-Dias, K. K. Temming, D. Temple, H. Ten Kate, P. K. Teng, J. J. Teoh, F. Tepel, S. Terada, K. Terashi, J. Terron, S. Terzo, M. Testa, R. J. Teuscher, T. Theveneaux-Pelzer, J. P. Thomas, J. Thomas-Wilsker, E. N. Thompson, P. D. Thompson, R. J. Thompson, A. S. Thompson, L. A. Thomsen, E. Thomson, M. Thomson, R. P. Thun, M. J. Tibbetts, R. E. Ticse Torres, V. O. Tikhomirov, Yu. A. Tikhonov, S. Timoshenko, E. Tiouchichine, P. Tipton, S. Tisserant, K. Todome, T. Todorov, S. Todorova-Nova, J. Tojo, S. Tokár, K. Tokushuku, K. Tollefson, E. Tolley, L. Tomlinson, M. Tomoto, L. Tompkins, K. Toms, E. Torrence, H. Torres, E. Torró Pastor, J. Toth, F. Touchard, D. R. Tovey, T. Trefzger, L. Tremblet, A. Tricoli, I. M. Trigger, S. Trincaz-Duvoid, M. F. Tripiana, W. Trischuk, B. Trocmé, C. Troncon, M. Trottier-McDonald, M. Trovatelli, P. True, L. Truong, M. Trzebinski, A. Trzupek, C. Tsarouchas, J. C-L. Tseng, P. V. Tsiareshka, D. Tsionou, G. Tsipolitis, N. Tsirintanis, S. Tsiskaridze, V. Tsiskaridze, E. G. Tskhadadze, I. I. Tsukerman, V. Tsulaia, S. Tsuno, D. Tsybychev, A. Tudorache, V. Tudorache, A. N. Tuna, S. A. Tupputi, S. Turchikhin, D. Turecek, R. Turra, A. J. Turvey, P. M. Tuts, A. Tykhonov, M. Tylmad, M. Tyndel, I. Ueda, R. Ueno, M. Ughetto, M. Ugland, F. Ukegawa, G. Unal, A. Undrus, G. Unel, F. C. Ungaro, Y. Unno, C. Unverdorben, J. Urban, P. Urquijo, P. Urrejola, G. Usai, A. Usanova, L. Vacavant, V. Vacek, B. Vachon, C. Valderanis, N. Valencic, S. Valentinetti, A. Valero, L. Valery, S. Valkar, E. Valladolid Gallego, S. Vallecorsa, J. A. Valls Ferrer, W. Van Den Wollenberg, P. C. Van Der Deijl, R. van der Geer, H. van der Graaf, N. van Eldik, P. van Gemmeren, J. Van Nieuwkoop, I. van Vulpen, M. C. van Woerden, M. Vanadia, W. Vandelli, R. Vanguri, A. Vaniachine, F. Vannucci, G. Vardanyan, R. Vari, E. W. Varnes, T. Varol, D. Varouchas, A. Vartapetian, K. E. Varvell, F. Vazeille, T. Vazquez Schroeder, J. Veatch, L. M. Veloce, F. Veloso, T. Velz, S. Veneziano, A. Ventura, D. Ventura, M. Venturi, N. Venturi, A. Venturini, V. Vercesi, M. Verducci, W. Verkerke, J. C. Vermeulen, A. Vest, M. C. Vetterli, O. Viazlo, I. Vichou, T. Vickey, O. E. Vickey Boeriu, G. H. A. Viehhauser, S. Viel, R. Vigne, M. Villa, M. Villaplana Perez, E. Vilucchi, M. G. Vincter, V. B. Vinogradov, I. Vivarelli, F. Vives Vaque, S. Vlachos, D. Vladoiu, M. Vlasak, M. Vogel, P. Vokac, G. Volpi, M. Volpi, H. von der Schmitt, H. von Radziewski, E. von Toerne, V. Vorobel, K. Vorobev, M. Vos, R. Voss, J. H. Vossebeld, N. Vranjes, M. Vranjes Milosavljevic, V. Vrba, M. Vreeswijk, R. Vuillermet, I. Vukotic, Z. Vykydal, P. Wagner, W. Wagner, H. Wahlberg, S. Wahrmund, J. Wakabayashi, J. Walder, R. Walker, W. Walkowiak, C. Wang, F. Wang, H. Wang, H. Wang, J. Wang, J. Wang, K. Wang, R. Wang, S. M. Wang, T. Wang, T. Wang, X. Wang, C. Wanotayaroj, A. Warburton, C. P. Ward, D. R. Wardrope, A. Washbrook, C. Wasicki, P. M. Watkins, A. T. Watson, I. J. Watson, M. F. Watson, G. Watts, S. Watts, B. M. Waugh, S. Webb, M. S. Weber, S. W. Weber, J. S. Webster, A. R. Weidberg, B. Weinert, J. Weingarten, C. Weiser, H. Weits, P. S. Wells, T. Wenaus, T. Wengler, S. Wenig, N. Wermes, M. Werner, P. Werner, M. Wessels, J. Wetter, K. Whalen, A. M. Wharton, A. White, M. J. White, R. White, S. White, D. Whiteson, F. J. Wickens, W. Wiedenmann, M. Wielers, P. Wienemann, C. Wiglesworth, L. A. M. Wiik-Fuchs, A. Wildauer, H. G. Wilkens, H. H. Williams, S. Williams, C. Willis, S. Willocq, A. Wilson, J. A. Wilson, I. Wingerter-Seez, F. Winklmeier, B. T. Winter, M. Wittgen, J. Wittkowski, S. J. Wollstadt, M. W. Wolter, H. Wolters, B. K. Wosiek, J. Wotschack, M. J. Woudstra, K. W. Wozniak, M. Wu, M. Wu, S. L. Wu, X. Wu, Y. Wu, T. R. Wyatt, B. M. Wynne, S. Xella, D. Xu, L. Xu, B. Yabsley, S. Yacoob, R. Yakabe, M. Yamada, D. Yamaguchi, Y. Yamaguchi, A. Yamamoto, S. Yamamoto, T. Yamanaka, K. Yamauchi, Y. Yamazaki, Z. Yan, H. Yang, H. Yang, Y. Yang, W-M. Yao, Y. Yasu, E. Yatsenko, K. H. Yau Wong, J. Ye, S. Ye, I. Yeletskikh, A. L. Yen, E. Yildirim, K. Yorita, R. Yoshida, K. Yoshihara, C. Young, C. J. S. Young, S. Youssef, D. R. Yu, J. Yu, J. M. Yu, J. Yu, L. Yuan, S. P. Y. Yuen, A. Yurkewicz, I. Yusuff, B. Zabinski, R. Zaidan, A. M. Zaitsev, J. Zalieckas, A. Zaman, S. Zambito, L. Zanello, D. Zanzi, C. Zeitnitz, M. Zeman, A. Zemla, Q. Zeng, K. Zengel, O. Zenin, T. Ženiš, D. Zerwas, D. Zhang, F. Zhang, H. Zhang, J. Zhang, L. Zhang, R. Zhang, X. Zhang, Z. Zhang, X. Zhao, Y. Zhao, Z. Zhao, A. Zhemchugov, J. Zhong, B. Zhou, C. Zhou, L. Zhou, L. Zhou, N. Zhou, C. G. Zhu, H. Zhu, J. Zhu, Y. Zhu, X. Zhuang, K. Zhukov, A. Zibell, D. Zieminska, N. I. Zimine, C. Zimmermann, S. Zimmermann, Z. Zinonos, M. Zinser, M. Ziolkowski, L. Živković, G. Zobernig, A. Zoccoli, M. zur Nedden, G. Zurzolo, L. Zwalinski

**Affiliations:** 1 Department of Physics, University of Adelaide, Adelaide, Australia; 2 Physics Department, SUNY Albany, Albany, NY USA; 3 Department of Physics, University of Alberta, Edmonton, AB Canada; 4 Department of Physics, Ankara University, Ankara, Turkey; 5 Istanbul Aydin University, Istanbul, Turkey; 6 Division of Physics, TOBB University of Economics and Technology, Ankara, Turkey; 7 LAPP, CNRS/IN2P3 and Université Savoie Mont Blanc, Annecy-le-Vieux, France; 8 High Energy Physics Division, Argonne National Laboratory, Argonne, IL USA; 9 Department of Physics, University of Arizona, Tucson, AZ USA; 10 Department of Physics, The University of Texas at Arlington, Arlington, TX USA; 11 Physics Department, University of Athens, Athens, Greece; 12 Physics Department, National Technical University of Athens, Zografou, Greece; 13 Institute of Physics, Azerbaijan Academy of Sciences, Baku, Azerbaijan; 14 Institut de Física d’Altes Energies and Departament de Física de la Universitat Autònoma de Barcelona, Barcelona, Spain; 15 Institute of Physics, University of Belgrade, Belgrade, Serbia; 16 Department for Physics and Technology, University of Bergen, Bergen, Norway; 17 Physics Division, Lawrence Berkeley National Laboratory and University of California, Berkeley, CA USA; 18 Department of Physics, Humboldt University, Berlin, Germany; 19 Albert Einstein Center for Fundamental Physics and Laboratory for High Energy Physics, University of Bern, Bern, Switzerland; 20 School of Physics and Astronomy, University of Birmingham, Birmingham, UK; 21 Department of Physics, Bogazici University, Istanbul, Turkey; 22 Department of Physics Engineering, Gaziantep University, Gaziantep, Turkey; 23 Department of Physics, Dogus University, Istanbul, Turkey; 24 INFN Sezione di Bologna, Bologna, Italy; 25 Dipartimento di Fisica e Astronomia, Università di Bologna, Bologna, Italy; 26 Physikalisches Institut, University of Bonn, Bonn, Germany; 27 Department of Physics, Boston University, Boston, MA USA; 28 Department of Physics, Brandeis University, Waltham, MA USA; 29 Universidade Federal do Rio De Janeiro COPPE/EE/IF, Rio de Janeiro, Brazil; 30 Electrical Circuits Department, Federal University of Juiz de Fora (UFJF), Juiz de Fora, Brazil; 31 Federal University of Sao Joao del Rei (UFSJ), Sao Joao del Rei, Brazil; 32 Instituto de Fisica, Universidade de Sao Paulo, São Paulo, Brazil; 33 Physics Department, Brookhaven National Laboratory, Upton, NY USA; 34 National Institute of Physics and Nuclear Engineering, Bucharest, Romania; 35 Physics Department, National Institute for Research and Development of Isotopic and Molecular Technologies, Cluj Napoca, Romania; 36 University Politehnica Bucharest, Bucharest, Romania; 37 West University in Timisoara, Timisoara, Romania; 38 Departamento de Física, Universidad de Buenos Aires, Buenos Aires, Argentina; 39 Cavendish Laboratory, University of Cambridge, Cambridge, UK; 40 Department of Physics, Carleton University, Ottawa, ON Canada; 41 CERN, Geneva, Switzerland; 42 Enrico Fermi Institute, University of Chicago, Chicago, IL USA; 43 Departamento de Física, Pontificia Universidad Católica de Chile, Santiago, Chile; 44 Departamento de Física, Universidad Técnica Federico Santa María, Valparaiso, Chile; 45 Institute of High Energy Physics, Chinese Academy of Sciences, Beijing, China; 46 Department of Modern Physics, University of Science and Technology of China, Anhui, China; 47 Department of Physics, Nanjing University, Jiangsu, China; 48 School of Physics, Shandong University, Shandong, China; 49 Department of Physics and Astronomy, Shanghai Key Laboratory for Particle Physics and Cosmology, Shanghai Jiao Tong University, Shanghai, China; 50 Physics Department, Tsinghua University, Beijing, 100084 China; 51 Laboratoire de Physique Corpusculaire, Clermont Université and Université Blaise Pascal and CNRS/IN2P3, Clermont-Ferrand, France; 52 Nevis Laboratory, Columbia University, Irvington, NY USA; 53 Niels Bohr Institute, University of Copenhagen, Kobenhavn, Denmark; 54 INFN Gruppo Collegato di Cosenza, Laboratori Nazionali di Frascati, Frascati, Italy; 55 Dipartimento di Fisica, Università della Calabria, Rende, Italy; 56 AGH University of Science and Technology, Faculty of Physics and Applied Computer Science, Kraków, Poland; 57 Marian Smoluchowski Institute of Physics, Jagiellonian University, Kraków, Poland; 58 Institute of Nuclear Physics, Polish Academy of Sciences, Kraków, Poland; 59 Physics Department, Southern Methodist University, Dallas, TX USA; 60 Physics Department, University of Texas at Dallas, Richardson, TX USA; 61 DESY, Hamburg and Zeuthen, Germany; 62 Institut für Experimentelle Physik IV, Technische Universität Dortmund, Dortmund, Germany; 63 Institut für Kern- und Teilchenphysik, Technische Universität Dresden, Dresden, Germany; 64 Department of Physics, Duke University, Durham, NC USA; 65 SUPA-School of Physics and Astronomy, University of Edinburgh, Edinburgh, UK; 66 INFN Laboratori Nazionali di Frascati, Frascati, Italy; 67 Fakultät für Mathematik und Physik, Albert-Ludwigs-Universität, Freiburg, Germany; 68 Section de Physique, Université de Genève, Geneva, Switzerland; 69 INFN Sezione di Genova, Genova, Italy; 70 Dipartimento di Fisica, Università di Genova, Genova, Italy; 71 E. Andronikashvili Institute of Physics, Iv. Javakhishvili Tbilisi State University, Tbilisi, Georgia; 72 High Energy Physics Institute, Tbilisi State University, Tbilisi, Georgia; 73 II Physikalisches Institut, Justus-Liebig-Universität Giessen, Giessen, Germany; 74 SUPA-School of Physics and Astronomy, University of Glasgow, Glasgow, UK; 75 II Physikalisches Institut, Georg-August-Universität, Göttingen, Germany; 76 Laboratoire de Physique Subatomique et de Cosmologie, Université Grenoble-Alpes, CNRS/IN2P3, Grenoble, France; 77 Department of Physics, Hampton University, Hampton, VA USA; 78 Laboratory for Particle Physics and Cosmology, Harvard University, Cambridge, MA USA; 79 Kirchhoff-Institut für Physik, Ruprecht-Karls-Universität Heidelberg, Heidelberg, Germany; 80 Physikalisches Institut, Ruprecht-Karls-Universität Heidelberg, Heidelberg, Germany; 81 ZITI Institut für technische Informatik, Ruprecht-Karls-Universität Heidelberg, Mannheim, Germany; 82 Faculty of Applied Information Science, Hiroshima Institute of Technology, Hiroshima, Japan; 83 Department of Physics, The Chinese University of Hong Kong, Shatin, N.T. Hong Kong, China; 84 Department of Physics, The University of Hong Kong, Hong Kong, China; 85 Department of Physics, The Hong Kong University of Science and Technology, Clear Water Bay, Kowloon, Hong Kong, China; 86 Department of Physics, Indiana University, Bloomington, IN USA; 87 Institut für Astro- und Teilchenphysik, Leopold-Franzens-Universität, Innsbruck, Austria; 88 University of Iowa, Iowa City, IA USA; 89 Department of Physics and Astronomy, Iowa State University, Ames, IA USA; 90 Joint Institute for Nuclear Research, JINR Dubna, Dubna, Russia; 91 KEK, High Energy Accelerator Research Organization, Tsukuba, Japan; 92 Graduate School of Science, Kobe University, Kobe, Japan; 93 Faculty of Science, Kyoto University, Kyoto, Japan; 94 Kyoto University of Education, Kyoto, Japan; 95 Department of Physics, Kyushu University, Fukuoka, Japan; 96 Instituto de Física La Plata, Universidad Nacional de La Plata and CONICET, La Plata, Argentina; 97 Physics Department, Lancaster University, Lancaster, UK; 98 INFN Sezione di Lecce, Lecce, Italy; 99 Dipartimento di Matematica e Fisica, Università del Salento, Lecce, Italy; 100 Oliver Lodge Laboratory, University of Liverpool, Liverpool, UK; 101 Department of Physics, Jožef Stefan Institute and University of Ljubljana, Ljubljana, Slovenia; 102 School of Physics and Astronomy, Queen Mary University of London, London, UK; 103 Department of Physics, Royal Holloway University of London, Surrey, UK; 104 Department of Physics and Astronomy, University College London, London, UK; 105 Louisiana Tech University, Ruston, LA USA; 106 Laboratoire de Physique Nucléaire et de Hautes Energies, UPMC and Université Paris-Diderot and CNRS/IN2P3, Paris, France; 107 Fysiska institutionen, Lunds universitet, Lund, Sweden; 108 Departamento de Fisica Teorica C-15, Universidad Autonoma de Madrid, Madrid, Spain; 109 Institut für Physik, Universität Mainz, Mainz, Germany; 110 School of Physics and Astronomy, University of Manchester, Manchester, UK; 111 CPPM, Aix-Marseille Université and CNRS/IN2P3, Marseille, France; 112 Department of Physics, University of Massachusetts, Amherst, MA USA; 113 Department of Physics, McGill University, Montreal, QC Canada; 114 School of Physics, University of Melbourne, Melbourne, VIC Australia; 115 Department of Physics, The University of Michigan, Ann Arbor, MI USA; 116 Department of Physics and Astronomy, Michigan State University, East Lansing, MI USA; 117 INFN Sezione di Milano, Milano, Italy; 118 Dipartimento di Fisica, Università di Milano, Milano, Italy; 119 B.I. Stepanov Institute of Physics, National Academy of Sciences of Belarus, Minsk, Republic of Belarus; 120 National Scientific and Educational Centre for Particle and High Energy Physics, Minsk, Republic of Belarus; 121 Department of Physics, Massachusetts Institute of Technology, Cambridge, MA USA; 122 Group of Particle Physics, University of Montreal, Montreal, QC Canada; 123 P.N. Lebedev Institute of Physics, Academy of Sciences, Moscow, Russia; 124 Institute for Theoretical and Experimental Physics (ITEP), Moscow, Russia; 125 National Research Nuclear University MEPhI, Moscow, Russia; 126 D.V. Skobeltsyn Institute of Nuclear Physics, M.V. Lomonosov Moscow State University, Moscow, Russia; 127 Fakultät für Physik, Ludwig-Maximilians-Universität München, München, Germany; 128 Max-Planck-Institut für Physik (Werner-Heisenberg-Institut), München, Germany; 129 Nagasaki Institute of Applied Science, Nagasaki, Japan; 130 Graduate School of Science and Kobayashi-Maskawa Institute, Nagoya University, Nagoya, Japan; 131 INFN Sezione di Napoli, Naples, Italy; 132 Dipartimento di Fisica, Università di Napoli, Napoli, Italy; 133 Department of Physics and Astronomy, University of New Mexico, Albuquerque, NM USA; 134 Institute for Mathematics, Astrophysics and Particle Physics, Radboud University Nijmegen/Nikhef, Nijmegen, The Netherlands; 135 Nikhef National Institute for Subatomic Physics and University of Amsterdam, Amsterdam, The Netherlands; 136 Department of Physics, Northern Illinois University, DeKalb, IL USA; 137 Budker Institute of Nuclear Physics, SB RAS, Novosibirsk, Russia; 138 Department of Physics, New York University, New York, NY USA; 139 Ohio State University, Columbus, OH USA; 140 Faculty of Science, Okayama University, Okayama, Japan; 141 Homer L. Dodge Department of Physics and Astronomy, University of Oklahoma, Norman, OK USA; 142 Department of Physics, Oklahoma State University, Stillwater, OK USA; 143 Palacký University, RCPTM, Olomouc, Czech Republic; 144 Center for High Energy Physics, University of Oregon, Eugene, OR USA; 145 LAL, Université Paris-Sud and CNRS/IN2P3, Orsay, France; 146 Graduate School of Science, Osaka University, Osaka, Japan; 147 Department of Physics, University of Oslo, Oslo, Norway; 148 Department of Physics, Oxford University, Oxford, UK; 149 INFN Sezione di Pavia, Pavia, Italy; 150 Dipartimento di Fisica, Università di Pavia, Pavia, Italy; 151 Department of Physics, University of Pennsylvania, Philadelphia, PA USA; 152 National Research Centre ‘Kurchatov Institute’ B.P.Konstantinov Petersburg Nuclear Physics Institute, St. Petersburg, Russia; 153 INFN Sezione di Pisa, Pisa, Italy; 154 Dipartimento di Fisica E. Fermi, Università di Pisa, Pisa, Italy; 155 Department of Physics and Astronomy, University of Pittsburgh, Pittsburgh, PA USA; 156 Laboratório de Instrumentação e Física Experimental de Partículas-LIP, Lisboa, Portugal; 157 Faculdade de Ciências, Universidade de Lisboa, Lisboa, Portugal; 158 Department of Physics, University of Coimbra, Coimbra, Portugal; 159 Centro de Física Nuclear da Universidade de Lisboa, Lisboa, Portugal; 160 Departamento de Fisica, Universidade do Minho, Braga, Portugal; 161 Departamento de Fisica Teorica y del Cosmos and CAFPE, Universidad de Granada, Granada, Spain; 162 Dep Fisica and CEFITEC of Faculdade de Ciencias e Tecnologia, Universidade Nova de Lisboa, Caparica, Portugal; 163 Institute of Physics, Academy of Sciences of the Czech Republic, Praha, Czech Republic; 164 Czech Technical University in Praha, Praha, Czech Republic; 165 Faculty of Mathematics and Physics, Charles University in Prague, Praha, Czech Republic; 166 State Research Center Institute for High Energy Physics, Protvino, Russia; 167 Particle Physics Department, Rutherford Appleton Laboratory, Didcot, UK; 168 INFN Sezione di Roma, Roma, Italy; 169 Dipartimento di Fisica, Sapienza Università di Roma, Roma, Italy; 170 INFN Sezione di Roma Tor Vergata, Roma, Italy; 171 Dipartimento di Fisica, Università di Roma Tor Vergata, Roma, Italy; 172 INFN Sezione di Roma Tre, Roma, Italy; 173 Dipartimento di Matematica e Fisica, Università Roma Tre, Rome, Italy; 174 Faculté des Sciences Ain Chock, Réseau Universitaire de Physique des Hautes Energies-Université Hassan II, Casablanca, Morocco; 175 Centre National de l’Energie des Sciences Techniques Nucleaires, Rabat, Morocco; 176 Faculté des Sciences Semlalia, Université Cadi Ayyad, LPHEA-Marrakech, Marrakech, Morocco; 177 Faculté des Sciences, Université Mohamed Premier and LPTPM, Oujda, Morocco; 178 Faculté des Sciences, Université Mohammed V-Agdal, Rabat, Morocco; 179 DSM/IRFU (Institut de Recherches sur les Lois Fondamentales de l’Univers), CEA Saclay (Commissariat à l’Energie Atomique et aux Energies Alternatives), Gif-sur-Yvette, France; 180 Santa Cruz Institute for Particle Physics, University of California Santa Cruz, Santa Cruz, CA USA; 181 Department of Physics, University of Washington, Seattle, WA USA; 182 Department of Physics and Astronomy, University of Sheffield, Sheffield, UK; 183 Department of Physics, Shinshu University, Nagano, Japan; 184 Fachbereich Physik, Universität Siegen, Siegen, Germany; 185 Department of Physics, Simon Fraser University, Burnaby, BC Canada; 186 SLAC National Accelerator Laboratory, Stanford, CA USA; 187 Faculty of Mathematics, Physics & Informatics, Comenius University, Bratislava, Slovak Republic; 188 Department of Subnuclear Physics, Institute of Experimental Physics of the Slovak Academy of Sciences, Kosice, Slovak Republic; 189 Department of Physics, University of Cape Town, Cape Town, South Africa; 190 Department of Physics, University of Johannesburg, Johannesburg, South Africa; 191 School of Physics, University of the Witwatersrand, Johannesburg, South Africa; 192 Department of Physics, Stockholm University, Stockholm, Sweden; 193 The Oskar Klein Centre, Stockholm, Sweden; 194 Physics Department, Royal Institute of Technology, Stockholm, Sweden; 195 Departments of Physics & Astronomy and Chemistry, Stony Brook University, Stony Brook, NY USA; 196 Department of Physics and Astronomy, University of Sussex, Brighton, UK; 197 School of Physics, University of Sydney, Sydney, Australia; 198 Institute of Physics, Academia Sinica, Taipei, Taiwan; 199 Department of Physics, Technion: Israel Institute of Technology, Haifa, Israel; 200 Raymond and Beverly Sackler School of Physics and Astronomy, Tel Aviv University, Tel Aviv, Israel; 201 Department of Physics, Aristotle University of Thessaloniki, Thessaloníki, Greece; 202 International Center for Elementary Particle Physics and Department of Physics, The University of Tokyo, Tokyo, Japan; 203 Graduate School of Science and Technology, Tokyo Metropolitan University, Tokyo, Japan; 204 Department of Physics, Tokyo Institute of Technology, Tokyo, Japan; 205 Department of Physics, University of Toronto, Toronto, ON Canada; 206 TRIUMF, Vancouver, BC Canada; 207 Department of Physics and Astronomy, York University, Toronto, ON Canada; 208 Faculty of Pure and Applied Sciences, University of Tsukuba, Tsukuba, Japan; 209 Department of Physics and Astronomy, Tufts University, Medford, MA USA; 210 Centro de Investigaciones, Universidad Antonio Narino, Bogotá, Colombia; 211 Department of Physics and Astronomy, University of California Irvine, Irvine, CA USA; 212 INFN Gruppo Collegato di Udine, Sezione di Trieste, Udine, Italy; 213 ICTP, Trieste, Italy; 214 Dipartimento di Chimica, Fisica e Ambiente, Università di Udine, Udine, Italy; 215 Department of Physics, University of Illinois, Urbana, IL USA; 216 Department of Physics and Astronomy, University of Uppsala, Uppsala, Sweden; 217 Instituto de Física Corpuscular (IFIC) and Departamento de Física Atómica, Molecular y Nuclear and Departamento de Ingeniería Electrónica and Instituto de Microelectrónica de Barcelona (IMB-CNM), University of Valencia and CSIC, Valencia, Spain; 218 Department of Physics, University of British Columbia, Vancouver, BC Canada; 219 Department of Physics and Astronomy, University of Victoria, Victoria, BC Canada; 220 Department of Physics, University of Warwick, Coventry, UK; 221 Waseda University, Tokyo, Japan; 222 Department of Particle Physics, The Weizmann Institute of Science, Rehovot, Israel; 223 Department of Physics, University of Wisconsin, Madison, WI USA; 224 Fakultät für Physik und Astronomie, Julius-Maximilians-Universität, Würzburg, Germany; 225 Fachbereich C Physik, Bergische Universität Wuppertal, Wuppertal, Germany; 226 Department of Physics, Yale University, New Haven, CT USA; 227 Yerevan Physics Institute, Yerevan, Armenia; 228 Centre de Calcul de l’Institut National de Physique Nucléaire et de Physique des Particules (IN2P3), Villeurbanne, France; 229 CERN, Geneva, Switzerland

## Abstract

A search is presented for a high-mass Higgs boson in the $H\rightarrow ZZ\rightarrow \ell ^+\ell ^-\ell ^+\ell ^-$, $H\rightarrow ZZ\rightarrow \ell ^+\ell ^-\nu \bar{\nu }$, $H\rightarrow ZZ\rightarrow \ell ^+\ell ^- q \bar{q}$, and $H\rightarrow ZZ\rightarrow \nu \bar{\nu } q \bar{q}$ decay modes using the ATLAS detector at the CERN Large Hadron Collider. The search uses proton–proton collision data at a centre-of-mass energy of 8 TeV corresponding to an integrated luminosity of 20.3 fb$^{-1}$. The results of the search are interpreted in the scenario of a heavy Higgs boson with a width that is small compared with the experimental mass resolution. The Higgs boson mass range considered extends up to $1~\mathrm{TeV}$ for all four decay modes and down to as low as 140 $\mathrm{GeV}$, depending on the decay mode. No significant excess of events over the Standard Model prediction is found. A simultaneous fit to the four decay modes yields upper limits on the production cross-section of a heavy Higgs boson times the branching ratio to $Z$ boson pairs. 95 % confidence level upper limits range from 0.53 pb at $m_{H} =195$ GeV to 0.008 pb at $m_{H} =950$ GeV for the gluon-fusion production mode and from 0.31 pb at $m_{H} =195$ GeV to 0.009 pb at $m_{H} =950$ GeV for the vector-boson-fusion production mode. The results are also interpreted in the context of Type-I and Type-II two-Higgs-doublet models.

## Introduction

In 2012, a Higgs boson $h$ with a mass of 125 GeV was discovered by the ATLAS and CMS collaborations at the LHC [1, 2]. One of the most important remaining questions is whether the newly discovered particle is part of an extended scalar sector as postulated by various extensions to the Standard Model (SM) such as the two-Higgs-doublet model (2HDM) [3] and the electroweak-singlet (EWS) model [4]. These predict additional Higgs bosons, motivating searches at masses other than 125 GeV.

This paper reports four separate searches with the ATLAS detector for a heavy neutral scalar $H$ decaying into two SM $Z$ bosons, encompassing the decay modes $ZZ\rightarrow \ell ^+\ell ^-\ell ^+\ell ^-$, $ZZ\rightarrow \ell ^+\ell ^-\nu \bar{\nu }$, $ZZ\rightarrow \ell ^+\ell ^- q \bar{q}$, and $ZZ\rightarrow \nu \bar{\nu } q \bar{q}$, where $\ell$ stands for either an electron or a muon. These modes are referred to, respectively, as $\ell \ell \ell \ell$, $\ell \ell \nu \nu$, $\ell \ell qq$, and $\nu \nu qq$.

It is assumed that additional Higgs bosons would be produced predominantly via the gluon fusion (ggF) and vector-boson fusion (VBF) processes but that the ratio of the two production mechanisms is unknown in the absence of a specific model. For this reason, results are interpreted separately for ggF and VBF production modes. For Higgs boson masses below 200 GeV, associated production (*VH*, where $$V$$ stands for either a $$W$$ or a $$Z$$ boson) is important as well. In this mass range, only the $$\ell \ell \ell \ell $$ decay mode is considered. Due to its excellent mass resolution and high signal-to-background ratio, the $$\ell \ell \ell \ell $$ decay mode is well-suited for a search for a narrow resonance in the range $$140<m_{H} <500$$ GeV; thus, this search covers the $$m_{H} $$ range down to $$140$$GeV. The $$\ell \ell \ell \ell $$ search includes channels sensitive to *VH* production as well as to the VBF and ggF production modes. The $$\ell \ell qq $$ and $$\ell \ell \nu \nu $$ searches, covering $$m_{H} $$ ranges down to 200 and 240 GeV respectively, consider ggF and VBF channels only. The $$\nu \nu qq $$ search covers the $$m_{H} $$ range down to 400 GeV and does not distinguish between ggF and VBF production. Due to their higher branching ratios, the $$\ell \ell qq $$, $$\ell \ell \nu \nu $$, and $$\nu \nu qq $$ decay modes dominate at higher masses, and contribute to the overall sensitivity of the combined result. The $$m_{H} $$ range for all four searches extends up to 1000 GeV.

The ggF production mode for the $\ell \ell \ell \ell$ search is further divided into four channels based on lepton flavour, while the $\ell \ell \nu \nu$ search includes four channels, corresponding to two lepton flavours for each of the ggF and VBF production modes. For the $\ell \ell qq$ and $\nu \nu qq$ searches, the ggF production modes are divided into two subchannels each based on the number of $b$-tagged jets in the event. For Higgs boson masses above 700 GeV, jets from $Z$ boson decay are boosted and tend to be reconstructed as a single jet; the ggF $\ell \ell qq$ search includes an additional channel sensitive to such final states.

For each channel, a discriminating variable sensitive to $$m_{H} $$ is identified and used in a likelihood fit. The $$\ell \ell \ell \ell $$ and $$\ell \ell qq $$ searches use the invariant mass of the four-fermion system as the final discriminant, while the $$\ell \ell \nu \nu $$ and $$\nu \nu qq $$ searches use a transverse mass distribution. Distributions of these discriminants for each channel are combined in a simultaneous likelihood fit which estimates the rate of heavy Higgs boson production and simultaneously the nuisance parameters corresponding to systematic uncertainties. Additional distributions from background-dominated control regions also enter the fit in order to constrain nuisance parameters. Unless otherwise stated, all figures show shapes and normalizations determined from this fit. All results are interpreted in the scenario of a new Higgs boson with a narrow width, as well as in Type-I and Type-II 2HDMs.

The ATLAS collaboration has published results of searches for a Standard Model Higgs boson decaying in the $\ell \ell \ell \ell$, $\ell \ell qq$, and $\ell \ell \nu \nu$ modes with 4.7–4.8$~{\mathrm{fb}^{-1}}$ of data collected at $\sqrt{s}=7~\mathrm{TeV}$ [5–7]. A heavy Higgs boson with the width and branching fractions predicted by the SM was excluded at the 95 % confidence level in the ranges $182<m_{H} <233~\mathrm{GeV}$, $256<m_{H} <265~\mathrm{GeV}$, and $268<m_{H} <415~\mathrm{GeV}$ by the $\ell \ell \ell \ell$ mode; in the ranges $300 < m_{H} < 322~\mathrm{GeV}$ and $353 < m_{H} < 410~\mathrm{GeV}$ by the $\ell \ell qq$ mode; and in the range $319 < m_{H} < 558~\mathrm{GeV}$ by the $\ell \ell \nu \nu$ mode. The searches in this paper use a data set of $20.3~{\mathrm{fb}^{-1}}$ of $pp$ collision data collected at a centre-of-mass energy of $\sqrt{s}=8~\mathrm{TeV}$. Besides using a larger data set at a higher centre-of-mass energy, these searches improve on the earlier results by adding selections sensitive to VBF production for the $\ell \ell \ell \ell$, $\ell \ell qq$, and $\ell \ell \nu \nu$ decay modes and by further optimizing the event selection and other aspects of the analysis. In addition, the $\nu \nu qq$ decay mode has been added; finally, results of searches in all four decay modes are used in a combined search. The CMS Collaboration has also recently published a search for a heavy Higgs boson with SM width in $H\rightarrow ZZ$ decays [8]. Since the searches reported here use a narrow width for each Higgs boson mass hypothesis instead of the larger width corresponding to a SM Higgs boson, a direct comparison against earlier ATLAS results and the latest CMS results is not possible.

This paper is organized as follows. After a brief description of the ATLAS detector in Sect. 2, the simulation of the background and signal processes used in this analysis is outlined in Sect. 3. Section 4 summarizes the reconstruction of the final-state objects used by these searches. The event selection and background estimation for the four searches are presented in Sects. 5 to 8, and Sect. 9 discusses the systematic uncertainties common to all searches. Section 10 details the statistical combination of all the searches into a single limit, which is given in Sect. 11. Finally, Sect. 12 gives the conclusions.

## ATLAS detector

ATLAS is a multi-purpose detector [9] which provides nearly full solid-angle coverage around the interaction point.^1^ It consists of a tracking system (inner detector or ID) surrounded by a thin superconducting solenoid providing a 2 T magnetic field, electromagnetic and hadronic calorimeters, and a muon spectrometer (MS). The ID consists of pixel and silicon microstrip detectors covering the pseudorapidity region $|\eta | < 2.5$, surrounded by a transition radiation tracker (TRT), which improves electron identification in the region $|\eta | < 2.0$. The sampling calorimeters cover the region $|\eta | < 4.9$. The forward region ($3.2 < |\eta | < 4.9$) is instrumented with a liquid-argon (LAr) calorimeter for electromagnetic and hadronic measurements. In the central region, a high-granularity lead/LAr electromagnetic calorimeter covers $|\eta | < 3.2$. Hadron calorimetry is based on either steel absorbers with scintillator tiles ($|\eta | < 1.7$) or copper absorbers in LAr ($1.5 < |\eta | < 3.2$). The MS consists of three large superconducting toroids arranged with an eight-fold azimuthal coil symmetry around the calorimeters, and a system of three layers of precision gas chambers providing tracking coverage in the range $|\eta | < 2.7$, while dedicated chambers allow triggering on muons in the region $|\eta | < 2.4$. The ATLAS trigger system [10] consists of three levels; the first (L1) is a hardware-based system, while the second and third levels are software-based systems.

## Data and Monte Carlo samples

### Data sample

The data used in these searches were collected by ATLAS at a centre-of-mass energy of 8 TeV during 2012 and correspond to an integrated luminosity of $20.3~{\mathrm{fb}^{-1}}$.

Collision events are recorded only if they are selected by the online trigger system. For the $\nu \nu qq$ search this selection requires that the magnitude $E_{\text {T}}^{\text {miss}}$ of the missing transverse momentum vector (see Sect. 4) is above $80~\mathrm{GeV}$. Searches with leptonic final states use a combination of single-lepton and dilepton triggers in order to maximize acceptance. The main single-lepton triggers have a minimum $p_{\text {T}}$ (muons) or $E_{\text {T}}$ (electrons) threshold of $24~\mathrm{GeV}$ and require that the leptons are isolated. They are complemented with triggers with higher thresholds ($60~\mathrm{GeV}$ for electrons and $36~\mathrm{GeV}$ for muons) and no isolation requirement in order to increase acceptance at high $p_{\text {T}}$ and $E_{\text {T}}$. The dilepton triggers require two same-flavour leptons with a threshold of $12~\mathrm{GeV}$ for electrons and $13~\mathrm{GeV}$ for muons. The acceptance in the $\ell \ell \ell \ell$ search is increased further with an additional asymmetric dimuon trigger selecting one muon with $p_{\text {T}} >18~\mathrm{GeV}$ and another one with $p_{\text {T}} >8~\mathrm{GeV}$ and an electron–muon trigger with thresholds of $E_{\text {T}} ^{e}>12~\mathrm{GeV}$ and $p_{\text {T}} ^{\mu }>8~\mathrm{GeV}$.

### Signal samples and modelling

The acceptance and resolution for the signal of a narrow-width heavy Higgs boson decaying to a $Z$ boson pair are modelled using Monte Carlo (MC) simulation. Signal samples are generated using Powheg r1508 [11, 12], which calculates separately the gluon and vector-boson-fusion Higgs boson production processes up to next-to-leading order (NLO) in $\alpha _{\text {S}}$. The generated signal events are hadronized with Pythia  8.165 using the AU2 set of tunable parameters for the underlying event [13, 14]; Pythia also decays the $Z$ bosons into all modes considered in this search. The contribution from $Z$ boson decay to $\tau$ leptons is also included. The NLO CT10 [15] parton distribution function (PDF) is used. The associated production of Higgs bosons with a $W$ or $Z$ boson ($WH$ and $ZH$) is significant for $m_{H} <200~\mathrm{GeV}$. It is therefore included as a signal process for the $\ell \ell \ell \ell$ search for $m_{H} <400~\mathrm{GeV}$ and simulated using Pythia  8 with the LO CTEQ6L1 PDF set [16] and the AU2 parameter set. These samples are summarized in Table 1.

Besides model-independent results, a search in the context of a CP-conserving 2HDM [3] is also presented. This model has five physical Higgs bosons after electroweak symmetry breaking: two CP-even, $h$ and $H$; one CP-odd, $A$; and two charged, $H^{\pm }$. The model considered here has seven free parameters: the Higgs boson masses ($m_h$, $m_H$, $m_A$, $m_{H^{\pm }}$), the ratio of the vacuum expectation values of the two doublets ($\tan \beta$), the mixing angle between the CP-even Higgs bosons ($\alpha$), and the potential parameter $m_{12}^2$ that mixes the two Higgs doublets. The two Higgs doublets $\Phi _1$ and $\Phi _2$ can couple to leptons and up- and down-type quarks in several ways. In the Type-I model, $\Phi _2$ couples to all quarks and leptons, whereas for Type-II, $\Phi _1$ couples to down-type quarks and leptons and $\Phi _2$ couples to up-type quarks. The ‘lepton-specific’ model is similar to Type-I except for the fact that the leptons couple to $\Phi _1$, instead of $\Phi _2$; the ‘flipped’ model is similar to Type-II except that the leptons couple to $\Phi _2$, instead of $\Phi _1$. In all these models, the coupling of the $H$ boson to vector bosons is proportional to $\cos (\beta -\alpha )$. In the limit $\cos (\beta -\alpha ) \rightarrow 0$ the light CP-even Higgs boson, $h$, is indistinguishable from a SM Higgs boson with the same mass. In the context of $H\rightarrow ZZ$ decays there is no direct coupling of the Higgs boson to leptons, and so only the Type-I and -II interpretations are presented.

The production cross-sections for both the ggF and VBF processes are calculated using SusHi 1.3.0 [17–22], while the branching ratios are calculated with 2HDMC 1.6.4 [23]. For the branching ratio calculations it is assumed that $m_A = m_H = m_{H^{\pm }}$, $m_h = 125~\mathrm{GeV}$, and $m_{12}^2 = m_A^2 \tan \beta /(1+\tan \beta ^2)$. In the 2HDM parameter space considered in this analysis, the cross-section times branching ratio for $H\rightarrow ZZ$ with $m_{H} =200~\mathrm{GeV}$ varies from 2.4 fb to 10 pb for Type-I and from 0.5 fb to 9.4 pb for Type-II.

The width of the heavy Higgs boson varies over the parameter space of the 2HDM model, and may be significant compared with the experimental resolution. Since this analysis assumes a narrow-width signal, the 2HDM interpretation is limited to regions of parameter space where the width is less than 0.5 % of $$m_{H} $$ (significantly smaller than the detector resolution). In addition, the off-shell contribution from the light Higgs boson and its interference with the non-resonant $$ZZ$$ background vary over the 2HDM parameter space as the light Higgs boson couplings are modified from their SM values. Therefore the interpretation is further limited to regions of the parameter space where the light Higgs boson couplings are enhanced by less than a factor of three from their SM values; in these regions the variation is found to have a negligible effect.

### Background samples

Monte Carlo simulations are also used to model the shapes of distributions from many of the sources of SM background to these searches. Table 1 summarizes the simulated event samples along with the PDF sets and underlying-event tunes used. Additional samples are also used to compute systematic uncertainties as detailed in Sect. 9.

Sherpa  1.4.1 [24] includes the effects of heavy-quark masses in its modelling of the production of $W$ and $Z$ bosons along with additional jets ($V+\mathrm {jets}$). For this reason it is used to model these backgrounds in the hadronic $\ell \ell qq$ and $\nu \nu qq$ searches, which are subdivided based on whether the $Z$ boson decays into $b$-quarks or light-flavour quarks. The Alpgen 2.14 $W+\mathrm {jets}$ and $Z/\gamma ^{*}+\mathrm {jets}$ samples are generated with up to five hard partons and with the partons matched to final-state particle jets [25, 26]. They are used to describe these backgrounds in the other decay modes and also in the VBF channel of the $\ell \ell qq$ search^2^ since the additional partons in the matrix element give a better description of the VBF topology. The Sherpa (Alpgen) $Z/\gamma ^{*}+\mathrm {jets}$ samples have a dilepton invariant mass requirement of $m_{\ell \ell } > 40~\mathrm{GeV}$ ($60~\mathrm{GeV}$) at the generator level.

The background from the associated production of the $125~\mathrm{GeV}$ $h$ boson along with a $Z$ boson is non-negligible in the $\ell \ell qq$ and $\nu \nu qq$ searches and is taken into account. Contributions to $Zh$ from both $q\bar{q}$ annihilation and gluon fusion are included. The $q\bar{q} \rightarrow Zh$ samples take into account NLO electroweak corrections, including differential corrections as a function of $Z$ boson $p_{\text {T}}$ [27, 28]. The Higgs boson branching ratio is calculated using hdecay [29]. Further details can be found in Ref. [30].

Continuum $$ZZ^{(*)} $$ events form the dominant background for the $$\ell \ell \ell \ell $$ and $$\ell \ell \nu \nu $$ decay modes; this is modelled with a dedicated $$q\bar{q} \rightarrow ZZ^{(*)} $$ sample. This sample is corrected to match the calculation described in Ref. [31], which is next-to-next-to-leading order (NNLO) in $$\alpha _{\text {S}} $$, with a $$K$$-factor that is differential in $$m_{ZZ} $$. Higher-order electroweak effects are included following the calculation reported in Refs. [32, 33] by applying a $$K$$-factor based on the kinematics of the diboson system and the initial-state quarks, using a procedure similar to that described in Ref. [34]. The off-shell SM ggF Higgs boson process, the $$gg \rightarrow ZZ$$ continuum, and their interference are considered as backgrounds. These samples are generated at leading order (LO) in $$\alpha _{\text {S}} $$ using MCFM 6.1 [35] ($$\ell \ell \ell \ell $$) or gg2vv 3.1.3 [36, 37] ($$\ell \ell \nu \nu $$) but corrected to NNLO as a function of $$m_{ZZ} $$ [38] using the same procedure as described in Ref. [6]. For the $$\ell \ell qq$$ and $$\nu \nu qq$$ searches, the continuum $$ZZ^{(*)} $$ background is smaller so the $$q\bar{q} \rightarrow ZZ^{(*)} $$ sample is used alone. It is scaled to include the contribution from $$gg \rightarrow ZZ^{(*)} $$ using the $$gg \rightarrow ZZ^{(*)} $$ cross-section calculated by MCFM 6.1 [35].

For samples in which the hard process is generated with Alpgen or MC@NLO 4.03 [39], Herwig 6.520 [40] is used to simulate parton showering and fragmentation, with Jimmy  [41] used for the underlying-event simulation. Pythia 6.426 [42] is used for samples generated with MadGraph [43] and AcerMC  [44], while Pythia 8.165 [45] is used for the gg2vv 3.1.3 [36, 37], MCFM 6.1 [46], and Powheg samples. Sherpa implements its own parton showering and fragmentation model.

**Table 1:** Details of the generation of simulated signal and background event samples. For each physics process, the table gives the final states generated, the $H \rightarrow ZZ$ final states(s) for which they are used, the generator, the PDF set, and the underlying-event tune. For the background samples, the order in $\alpha _{\text {S}}$ used to normalize the event yield is also given; for the signal, the normalization is the parameter of interest in the fit. More details can be found in the text

Physics process	$H \rightarrow ZZ$ search final state	Generator	Cross-section normalization	PDF set	Tune
$W/Z$ boson + jets					
$Z/\gamma ^{*}\rightarrow \ell ^+ \ell ^-/\nu \bar{\nu }$	$\ell \ell \ell \ell /\ell \ell \nu \nu$	Alpgen 2.14 [25]	NNLO [47]	CTEQ6L1 [16]	AUET2 [14, 48]
	$\ell \ell qq^{\mathrm{a}}/\nu \nu qq$	Sherpa 1.4.1 [24]	NNLO [49, 50]	NLO CT10	Sherpa default
$W \rightarrow \ell \nu$	$\ell \ell \nu \nu$	Alpgen 2.14	NNLO [47]	CTEQ6L1	AUET2
	$\nu \nu qq$	Sherpa 1.4.1	NNLO [49, 50]	NLO CT10	Sherpa default
Top quark					
$t\bar{t}$	$\ell \ell \ell \ell /\ell \ell qq/\nu \nu qq$	Powheg-Box r2129 [51–53]	NNLO+NNLL	NLO CT10	Perugia2011C [54]
	$\ell \ell \nu \nu$	MC@NLO 4.03 [39]	[55, 56]		AUET2
$s$-channel and $Wt$	$\ell \ell \ell \ell /\ell \ell qq/\nu \nu qq$	Powheg-Box r1556	NNLO+NNLL	NLO CT10	Perugia2011C
	$\ell \ell \nu \nu$	MC@NLO 4.03	[57, 58]		AUET2
$t$-channel	All	AcerMC 3.8 [44]	NNLO+NNLL [59]	CTEQ6L1	AUET2
Dibosons					
$q\bar{q} \rightarrow ZZ(*)$	$\ell \ell qq/\nu \nu qq$	Powheg-Box r1508 [60]	NLO [35, 61]	NLO CT10	AUET2
	$\ell \ell \ell \ell /\ell \ell \nu \nu$	Powheg-Box r1508 [60]	NNLO QCD [31]	NLO CT10	AUET2
			NLO EW [32, 33]		
EW $q\bar{q}~(\rightarrow h) \rightarrow$ $ZZ(*) + 2j$	$\ell \ell \ell \ell$	MadGraph 5 1.3.28 [43]		CTEQ6L1	AUET2
$gg~(\rightarrow h^*) \rightarrow ZZ$	$\ell \ell \ell \ell$	MCFM 6.1 [46]	NNLO [38]	NLO CT10	AU2
	$\ell \ell \nu \nu$	GG2VV 3.1.3 [36, 37]	(for $h \rightarrow ZZ$)	NLO CT10	AU2
$q\bar{q} \rightarrow WZ$	$\ell \ell \nu \nu /\ell \ell qq/\nu \nu qq$	Powheg-Box r1508	NLO [35, 61]	NLO CT10	AUET2
	$\ell \ell \ell \ell$	Sherpa 1.4.1			Sherpa default
$q\bar{q} \rightarrow WW$	All	Powheg-Box r1508	NLO [35, 61]	NLO CT10	AUET2
$m_{h} =125~\mathrm{GeV}$ SM Higgs boson (background)$^{\mathrm{b}}$					
$q\bar{q} \rightarrow Zh \rightarrow$ $\ell ^+\ell ^- b\bar{b} / \nu \bar{\nu }b\bar{b}$	$\ell \ell qq/\nu \nu qq$	Pythia 8.165	NNLO [62–64]	CTEQ6L	AU2
$gg \rightarrow Zh \rightarrow$ $\ell ^+\ell ^- b\bar{b} / \nu \bar{\nu }b\bar{b}$	$\ell \ell qq/\nu \nu qq$	Powheg-Box r1508	NLO [65]	CT10	AU2
Signal					
$gg\rightarrow H \rightarrow ZZ(*)$	All	Powheg-Box r1508	–	NLO CT10	AU2
$q\bar{q}\rightarrow H+2j$; $H \rightarrow ZZ(*)$	all	Powheg-Box r1508	–	NLO CT10	AU2
$q\bar{q}\rightarrow (W/Z)H$; $H \rightarrow ZZ(*)$	$\ell \ell \ell \ell$	Pythia 8.163	–	CTEQ6L1	AU2

$^{\mathrm{a}}$ The $H\rightarrow ZZ\rightarrow \ell ^+\ell ^- q \bar{q}$ VBF search uses Alpgen instead

$^{\mathrm{b}}$ For the $H\rightarrow ZZ\rightarrow \ell ^+\ell ^-\ell ^+\ell ^-$ and $H\rightarrow ZZ\rightarrow \ell ^+\ell ^-\nu \bar{\nu }$ searches, the SM $h\rightarrow ZZ$ boson contribution, along with its interference with the continuum $ZZ$ background, is included in the diboson samples

In the $\ell \ell qq$ and $\nu \nu qq$ searches, which have jets in the final state, the principal background is $V+\mathrm {jets}$, where $V$ stands for either a $W$ or a $Z$ boson. In simulations of these backgrounds, jets are labelled according to which generated hadrons with $p_{\text {T}} >5~\mathrm{GeV}$ are found within a cone of size $\Delta R = 0.4$ around the reconstructed jet axis. If a $b$-hadron is found, the jet is labelled as a $b$-jet; if not and a charmed hadron is found, the jet is labelled as a $c$-jet; if neither is found, the jet is labelled as a light (i.e., $u$-, $d$-, or $s$-quark, or gluon) jet, denoted by ‘$j$’. For $V+\mathrm {jets}$ events that pass the selections for these searches, two of the additional jets are reconstructed as the hadronically-decaying $Z$ boson candidate. Simulated $V+\mathrm {jets}$ events are then categorized based on the labels of these jets. If one jet is labelled as a $b$-jet, the event belongs to the $V+b$ category; if not, and one of the jets is labelled as a $c$-jet, the event belongs to the $V+c$ category; otherwise, the event belongs to the $V+j$ category. Further subdivisions are defined according to the flavour of the other jet from the pair, using the same precedence order: $V+bb$, $V+bc$, $V+bj$, $V+cc$, $V+cj$, and $V+jj$; the combination of $V+bb$, $V+bc$, and $V+cc$ is denoted by $V$ $+$ hf.

### Detector simulation

The simulation of the detector is performed with either a full ATLAS detector simulation [66] based on Geant  4 9.6 [67] or a fast simulation^3^ based on a parameterization of the performance of the ATLAS electromagnetic and hadronic calorimeters [68] and on Geant  4 elsewhere. All simulated samples are generated with a variable number of minimum-bias interactions (simulated using Pythia  8 with the MSTW2008LO PDF [69] and the A2 tune [48]), overlaid on the hard-scattering event to account for additional $pp$ interactions in either the same or a neighbouring bunch crossing (pile-up).

Corrections are applied to the simulated samples to account for differences between data and simulation for the lepton trigger and reconstruction efficiencies, and for the efficiency and misidentification rate of the algorithm used to identify jets containing $b$-hadrons ($b$-tagging).

## Object reconstruction and common event selection

The exact requirements used to identify physics objects vary between the different searches. This section outlines features that are common to all of the searches; search-specific requirements are given in the sections below.

Event vertices are formed from tracks with $p_{\text {T}} >400~\mathrm{MeV}$. Each event must have an identified primary vertex, which is chosen from among the vertices with at least three tracks as the one with the largest $\sum p_{\text {T}} ^2$ of associated tracks.

Muon candidates (‘muons’) [70] generally consist of a track in the ID matched with one in the MS. However, in the forward region ($2.5<|\eta |<2.7$), MS tracks may be used with no matching ID tracks; further, around $|\eta |=0$, where there is a gap in MS coverage, ID tracks with no matching MS track may be used if they match an energy deposit in the calorimeter consistent with a muon. In addition to quality requirements, muon tracks are required to pass close to the reconstructed primary event vertex. The longitudinal impact parameter, $z_0$, is required to be less than $10\mathrm{mm}$, while the transverse impact parameter, $d_0$, is required to be less than $1\mathrm{mm}$ to reject non-collision backgrounds. This requirement is not applied in the case of muons with no ID track.

Electron candidates (‘electrons’) [71–73] consist of an energy cluster in the EM calorimeter with $|\eta |<2.47$ matched to a track reconstructed in the inner detector. The energy of the electron is measured from the energy of the calorimeter cluster, while the direction is taken from the matching track. Electron candidates are selected using variables sensitive to the shape of the EM cluster, the quality of the track, and the goodness of the match between the cluster and the track. Depending on the search, either a selection is made on each variable sequentially or all the variables are combined into a likelihood discriminant.

Electron and muon energies are calibrated from measurements of $Z\rightarrow ee/\mu \mu$ decays [70, 72]. Electrons and muons must be isolated from other tracks, using $p_{\text {T}} ^{\ell ,\mathrm {isol}} / p_{\text {T}} ^{\ell }<0.1$, where $p_{\text {T}} ^{\ell ,\mathrm {isol}}$ is the scalar sum of the transverse momenta of tracks within a $\Delta R = 0.2$ cone around the electron or muon (excluding the electron or muon track itself), and $p_{\text {T}} ^{\ell }$ is the transverse momentum of the electron or muon candidate. The isolation requirement is not applied in the case of muons with no ID track. For searches with electrons or muons in the final state, the reconstructed lepton candidates must match the trigger lepton candidates that resulted in the events being recorded by the online selection.

Jets are reconstructed [74] using the anti-$k_t$ algorithm [75] with a radius parameter $R=0.4$ operating on massless calorimeter energy clusters constructed using a nearest-neighbour algorithm. Jet energies and directions are calibrated using energy- and $\eta$-dependent correction factors derived using MC simulations, with an additional calibration applied to data samples derived from in situ measurements [76]. A correction is also made for effects of energy from pile-up. For jets with $p_{\text {T}} <50~\mathrm{GeV}$ within the acceptance of the ID ($|\eta |<2.4$), the fraction of the summed scalar $p_{\text {T}}$ of the tracks associated with the jet (within a $\Delta R=0.4$ cone around the jet axis) contributed by those tracks originating from the primary vertex must be at least 50 %. This ratio is called the jet vertex fraction (JVF), and this requirement reduces the number of jet candidates originating from pile-up vertices [77, 78].

In the $\ell \ell qq$ search at large Higgs boson masses, the decay products of the boosted $Z$ boson may be reconstructed as a single anti-$k_t$ jet with a radius of $R=0.4$. Such configurations are identified using the jet invariant mass, obtained by summing the momenta of the jet constituents. After the energy calibration, the jet masses are calibrated, based on Monte Carlo simulations, as a function of jet $p_{\text {T}}$, $\eta$, and mass.

The missing transverse momentum, with magnitude $E_{\text {T}}^{\text {miss}}$, is the negative vectorial sum of the transverse momenta from calibrated objects, such as identified electrons, muons, photons, hadronic decays of tau leptons, and jets [79]. Clusters of calorimeter cells not matched to any object are also included.

Jets containing $b$-hadrons ($b$-jets) can be discriminated from other jets (‘tagged’) based on the relatively long lifetime of $b$-hadrons. Several methods are used to tag jets originating from the fragmentation of a $b$-quark, including looking for tracks with a large impact parameter with respect to the primary event vertex, looking for a secondary decay vertex, and reconstructing a $b$-hadron $\rightarrow$ $c$ hadron decay chain. For the $\ell \ell qq$ and $\nu \nu qq$ searches, this information is combined into a single neural-network discriminant (‘MV1c’). This is a continuous variable that is larger for jets that are more like $b$-jets. A selection is then applied that gives an efficiency of about 70 %, on average, for identifying true $b$-jets, while the efficiencies for accepting $c$-jets or light-quark jets are 1/5 and 1/140 respectively [30, 80–83]. The $\ell \ell \nu \nu$ search uses an alternative version of this discriminant, ‘MV1’ [80], to reject background due to top-quark production; compared with MV1c it has a smaller $c$-jet rejection. Tag efficiencies and mistag rates are calibrated using data. For the purpose of forming the invariant mass of the $b$-jets, $m_{bb}$, the energies of $b$-tagged jets are corrected to account for muons within the jets and an additional $p_{\text {T}}$-dependent correction is applied to account for biases in the response due to resolution effects.

In channels which require two $b$-tagged jets in the final state, the efficiency for simulated events of the dominant $Z+\mathrm {jets}$ background to pass the $b$-tagging selection is low. To effectively increase the sizes of simulated samples, jets are ‘truth tagged’: each event is weighted by the flavour-dependent probability of the jets to actually pass the $b$-tagging selection.

## $H\rightarrow ZZ\rightarrow \ell ^+\ell ^-\ell ^+\ell ^-$ event selection and background estimation

### Event selection

The event selection and background estimation for the $H\rightarrow ZZ\rightarrow \ell ^+\ell ^-\ell ^+\ell ^-$ ($\ell \ell \ell \ell$) search is very similar to the analysis described in Ref. [84]. More details may be found there; a summary is given here.

Higgs boson candidates in the $$\ell \ell \ell \ell $$ search must have two same-flavour, opposite-charge lepton pairs. Muons must satisfy $$p_{\text {T}} >6~\mathrm{GeV}$$ and $$|\eta |<2.7$$, while electrons are identified using the likelihood discriminant corresponding to the ‘loose LH’ selection from Ref. [73] and must satisfy $$p_{\text {T}} >7~\mathrm{GeV}$$. The impact parameter requirements that are made for muons are also applied to electrons, and electrons (muons) must also satisfy a requirement on the transverse impact parameter significance, $$|d_0|/\sigma _{d_0} < 6.5$$ (3.5). For this search, the track-based isolation requirement is relaxed to $$p_{\text {T}} ^{\ell ,\mathrm {isol}}/p_{\text {T}} ^{\ell } < 0.15$$ for both the electrons and muons. In addition, lepton candidates must also be isolated in $$E_{\text {T}} ^{\ell ,\mathrm {isol}}$$, the sum of the transverse energies in calorimeter cells within a $$\Delta R = 0.2$$ cone around the candidate (excluding the deposit from the candidate itself). The requirement is $$E_{\text {T}} ^{\ell ,\mathrm {isol}} / p_{\text {T}} ^{\ell }<0.2$$ for electrons, $${<}0.3$$ for muons with a matching ID track, and $${<}0.15$$ for other muons. The three highest-$$p_{\text {T}} $$ leptons in the event must satisfy, in order, $$p_{\text {T}} > 20$$, $$15$$, and $$10~\mathrm{GeV}$$. To ensure well-measured leptons, and reduce backgrounds containing electrons from bremsstrahlung, same-flavour leptons must be separated from each other by $$\Delta R> 0.1$$, and different-flavour leptons by $$\Delta R>0.2$$. Jets that are $$\Delta R<0.2$$ from electrons are removed. Final states in this search are classified depending on the flavours of the leptons present: $$4\mu $$, $$2e2\mu $$, $$2\mu 2e$$, and $$4e$$. The selection of lepton pairs is made separately for each of these flavour combinations; the pair with invariant mass closest to the $$Z$$ boson mass is called the leading pair and its invariant mass, $$m_{12} $$, must be in the range $$50$$–$$106~\mathrm{GeV}$$. For the $$2e2\mu $$ channel, the electrons form the leading pair, while for the $$2\mu 2e$$ channel the muons are leading. The second, subleading, pair of each combination is the pair from the remaining leptons with invariant mass $$m_{34} $$ closest to that of the $$Z$$ boson in the range $$m_{\mathrm {min}} < m_{34} < 115~\mathrm{GeV}$$. Here $$m_{\mathrm {min}} $$ is $$12~\mathrm{GeV}$$ for $$m_{\ell \ell \ell \ell } < 140~\mathrm{GeV}$$, rises linearly to $$50~\mathrm{GeV}$$ at $$m_{\ell \ell \ell \ell } =190~\mathrm{GeV}$$, and remains at $$50~\mathrm{GeV}$$ for $$m_{\ell \ell \ell \ell } > 190~\mathrm{GeV}$$. Finally, if more than one flavour combination passes the selection, which could happen for events with more than four leptons, the flavour combination with the highest expected signal acceptance is kept; i.e., in the order: $$4\mu $$, $$2e2\mu $$, $$2\mu 2e$$, and $$4e$$. For $$4\mu $$ and $$4e$$ events, if an opposite-charge same-flavour dilepton pair is found with $$m_{\ell \ell } $$ below $$5~\mathrm{GeV}$$, the event is vetoed in order to reject backgrounds from $$J/\psi $$ decays.

To improve the mass resolution, the four-momentum of any reconstructed photon consistent with having been radiated from one of the leptons in the leading pair is added to the final state. Also, the four-momenta of the leptons in the leading pair are adjusted by means of a kinematic fit assuming a $$Z\rightarrow \ell \ell $$ decay; this improves the $$m_{\ell \ell \ell \ell } $$ resolution by up to 15 %, depending on $$m_{H}$$. This is not applied to the subleading pair in order to retain sensitivity at lower $$m_{H} $$ where one of the $$Z$$ boson decays may be off-shell. For $$4\mu $$ events, the resulting mass resolution varies from 1.5 % at $$m_{H} =200~\mathrm{GeV}$$ to 3.5 % at $$m_{H} =1~\mathrm{TeV}$$, while for $$4e$$ events it ranges from 2 % at $$m_{H} =200~\mathrm{GeV}$$ to below 1 % at $$1~\mathrm{TeV}$$.

**Fig. 1:**
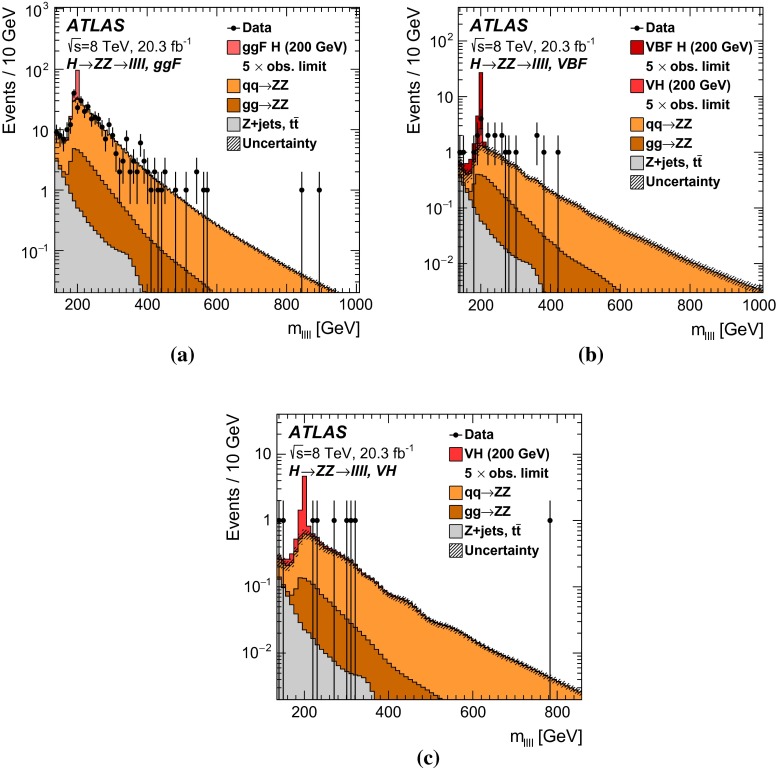
The distributions used in the likelihood fit of the four-lepton invariant mass $m_{\ell \ell \ell \ell }$ for the $H\rightarrow ZZ\rightarrow \ell ^+\ell ^-\ell ^+\ell ^-$ search in the **a** ggF, **b** VBF, and **c** $VH$ channels. The ‘$Z+\mathrm {jets}$, $t\bar{t}$’ entry includes all backgrounds other than $ZZ$, as measured from data. No events are observed beyond the upper limit of the plots. The simulated $m_{H} =200~\mathrm{GeV}$ signal is normalized to a cross-section corresponding to five times the observed limit given in Sect. 11. Both the VBF and $VH$ signal modes are shown in **b** as there is significant contamination of $VH$ events in the VBF category

Signal events can be produced via ggF or VBF, or associated production (*VH*, where $$V$$ stands for either a $$W$$ or a $$Z$$ boson). In order to measure the rates for these processes separately, events passing the event selection described above are classified into channels, either ggF, VBF, or *VH*. Events containing at least two jets with $$p_{\text {T}} > 25~\mathrm{GeV}$$ and $$|\eta |<2.5$$ or $$p_{\text {T}} > 30~\mathrm{GeV}$$ and $$2.5<|\eta |<4.5$$ and with the leading two such jets having $$m_{jj} >130~\mathrm{GeV}$$ are classified as VBF events. Otherwise, if a jet pair satisfying the same $$p_{\text {T}} $$ and $$\eta $$ requirements is present but with $$40<m_{jj} <130~\mathrm{GeV}$$, the event is classified as *VH*, providing it also passes a selection on a multivariate discriminant used to separate the *VH* and ggF signal. The multivariate discriminant makes use of $$m_{jj} $$, $$\Delta \eta _{jj} $$, the $$p_{\text {T}} $$ of the two jets, and the $$\eta $$ of the leading jet. In order to account for leptonic decays of the $$V$$ ($$W$$ or $$Z$$) boson, events failing this selection may still be classified as *VH* if an additional lepton with $$p_{\text {T}} >8~\mathrm{GeV}$$ is present. All remaining events are classified as ggF. Due to the differing background compositions and signal resolutions, events in the ggF channel are further classified into subchannels according to their final state: $$4e$$, $$2e2\mu $$, $$2\mu 2e$$, or $$4\mu $$. The selection for VBF is looser than that used in the other searches; however, the effect on the final results is small. The $$m_{\ell \ell \ell \ell } $$ distributions for the three channels are shown in Fig. 1.

### Background estimation

The dominant background in this channel is continuum $ZZ^{(*)}$ production. Its contribution to the yield is determined from simulation using the samples described in Sect. 3.3. Other background components are small and consist mainly of $t\bar{t}$ and $Z+\mathrm {jets}$ events. These are difficult to estimate from MC simulations due to the small rate at which such events pass the event selection, and also because they depend on details of jet fragmentation, which are difficult to model reliably in simulations. Therefore, both the rate and composition of these backgrounds are estimated from data. Since the composition of these backgrounds depends on the flavour of the subleading dilepton pair, different approaches are taken for the $\ell \ell \mu \mu$ and the $\ell \ell ee$ final states.

The $\ell \ell \mu \mu$ non-$ZZ$ background comprises mostly $t\bar{t}$ and $Z+b\bar{b}$ events, where in the latter the muons arise mostly from heavy-flavour semileptonic decays, and to a lesser extent from $\pi$/$K$  in-flight decays. The contribution from single-top production is negligible. The normalization of each component is estimated by a simultaneous fit to the $m_{12}$ distribution in four control regions, defined by inverting the impact parameter significance or isolation requirements on the subleading muon, or by selecting a subleading $e\mu$ or same-charge pair. A small contribution from $WZ$ decays is estimated using simulation. The electron background contributing to the $\ell \ell ee$ final states comes mainly from jets misidentified as electrons, arising in three ways: light-flavour hadrons misidentified as electrons, photon conversions reconstructed as electrons, and non-isolated electrons from heavy-flavour hadronic decays. This background is estimated in a control region in which the three highest-$p_{\text {T}}$ leptons must satisfy the full selection, with the third lepton being an electron. For the lowest-$p_{\text {T}}$ lepton, which must also be an electron, the impact parameter and isolation requirements are removed and the likelihood requirement is relaxed. In addition, it must have the same charge as the other subleading electron in order to minimize the contribution from the $ZZ^{(*)}$ background. The yields of the background components of the lowest-$p_{\text {T}}$ lepton are extracted with a fit to the number of hits in the innermost pixel layer and the ratio of the number of high-threshold to low-threshold TRT hits (which provides discrimination between electrons and pions). For both backgrounds, the fitted yields in the control regions are extrapolated to the signal region using efficiencies obtained from simulation.

For the non-$ZZ$ components of the background, the $m_{\ell \ell \ell \ell }$ shape is evaluated for the $\ell \ell \mu \mu$ final states using simulated events, and from data for the $\ell \ell ee$ final states by extrapolating the shape from the $\ell \ell ee$ control region described above. The fraction of this background in each channel (ggF, VBF, *VH*) is evaluated using simulation. The non-$ZZ$ background contribution for $m_{\ell \ell \ell \ell } >140~\mathrm{GeV}$ is found to be approximately 4 % of the total background.

Major sources of uncertainty in the estimate of the non-$ZZ$ backgrounds include differences in the results when alternative methods are used to estimate the background [84], uncertainties in the transfer factors used to extrapolate from the control region to the signal region, and the limited statistical precision in the control regions. For the $\ell \ell \mu \mu$ ($\ell \ell ee$) background, the uncertainty is 21 % (27 %) in the ggF channel, 100 % (117 %) in the VBF channel, and 62 % (79 %) in the *VH* channel. The larger uncertainty in the VBF channel arises due to large statistical uncertainties on the fraction of $Z+\mathrm {jets}$ events falling in this channel. Uncertainties in the expected $m_{\ell \ell \ell \ell }$ shape are estimated from differences in the shapes obtained using different methods for estimating the background.

## $H\rightarrow ZZ\rightarrow \ell ^+\ell ^-\nu \bar{\nu }$ event selection and background estimation

### Event selection

The event selection for the $H\rightarrow ZZ\rightarrow \ell ^+\ell ^-\nu \bar{\nu }$ ($\ell \ell \nu \nu$) search starts with the reconstruction of either a $Z\rightarrow e^+e^-$ or $Z\rightarrow \mu ^+\mu ^-$ lepton pair; the leptons must be of opposite charge and must have invariant mass $76<m_{\ell \ell } <106~\mathrm{GeV}$. The charged lepton selection is tighter than that described in Sect. 4. Muons must have matching tracks in the ID and MS and lie in the region $|\eta |<2.5$. Electrons are identified using a series of sequential requirements on the discriminating variables, corresponding to the ‘medium’ selection from Ref. [73]. Candidate leptons for the $Z\rightarrow \ell ^+\ell ^-$ decay must have $p_{\text {T}} >20~\mathrm{GeV}$, and leptons within a cone of $\Delta R=0.4$ around jets are removed. Jets that lie $\Delta R<0.2$ of electrons are also removed. Events containing a third lepton or muon with $p_{\text {T}} >7~\mathrm{GeV}$ are rejected; for the purpose of this requirement, the ‘loose’ electron selection from Ref. [73] is used. To select events with neutrinos in the final state, the magnitude of the missing transverse momentum must satisfy $E_{\text {T}}^{\text {miss}} > 70~\mathrm{GeV}$.

As in the $\ell \ell \ell \ell$ search, samples enriched in either ggF or VBF production are selected. An event is classified as VBF if it has at least two jets with $p_{\text {T}} >30~\mathrm{GeV}$ and $|\eta |<4.5$ with $m_{jj} >550~\mathrm{GeV}$ and $\Delta \eta _{jj} >4.4$. Events failing to satisfy the VBF criteria and having no more than one jet with $p_{\text {T}} >30~\mathrm{GeV}$ and $|\eta |<2.5$ are classified as ggF. Events not satisfying either set of criteria are rejected.

To suppress the Drell–Yan background, the azimuthal angle between the combined dilepton system and the missing transverse momentum vector $\Delta \phi (p_{\text {T}}^{\ell \ell },E_{\text {T}}^{\text {miss}})$ must be greater than 2.8 (2.7) for the ggF (VBF) channel (optimized for signal significance in each channel), and the fractional $p_{\text {T}}$ difference, defined as $|p_{\text {T}}^{\mathrm {miss,jet}}- p_{\text {T}}^{\ell \ell } |/p_{\text {T}}^{\ell \ell }$, must be less than 20 %, where $p_{\text {T}}^{\mathrm {miss,jet}} =\bigl | {{\vec {}}{E_{\text {T}}^{\text {miss}} }} + \sum _{\mathrm {jet}} {{\vec {}}{p_{\text {T}} }} ^{\mathrm {jet}}\bigr |$. $Z$ bosons originating from the decay of a high-mass state are boosted; thus, the azimuthal angle between the two leptons $\Delta \phi _{\ell \ell }$ must be less than 1.4. Events containing a $b$-tagged jet with $p_{\text {T}} >20~\mathrm{GeV}$ and $|\eta |<2.5$ are rejected in order to reduce the background from top-quark production. All jets in the event must have an azimuthal angle greater than 0.3 relative to the missing transverse momentum.

The discriminating variable used is the transverse mass $m_{\mathrm {T}}^{ZZ}$ reconstructed from the momentum of the dilepton system and the missing transverse momentum, defined by:

1 $$\begin{aligned} (m_{\mathrm {T}}^{ZZ})^2&\equiv \left( \sqrt{m_{Z} ^2+\left| p_{\text {T}}^{\ell \ell } \right| ^2} + \sqrt{m_{Z} ^2+\left| E_{\text {T}}^{\text {miss}} \right| ^2} \right) ^2\nonumber \\&-\,\left|  {{\vec {}}{p_{\text {T}}^{\ell \ell } }} +  {{\vec {}}{E_{\text {T}}^{\text {miss}} }} \right| ^2. \end{aligned}$$

The resulting resolution in $m_{\mathrm {T}}^{ZZ}$ ranges from 7 % at $m_{H} =240~\mathrm{GeV}$ to 15 % at $m_{H} =1~\mathrm{TeV}$.

**Fig. 2:**
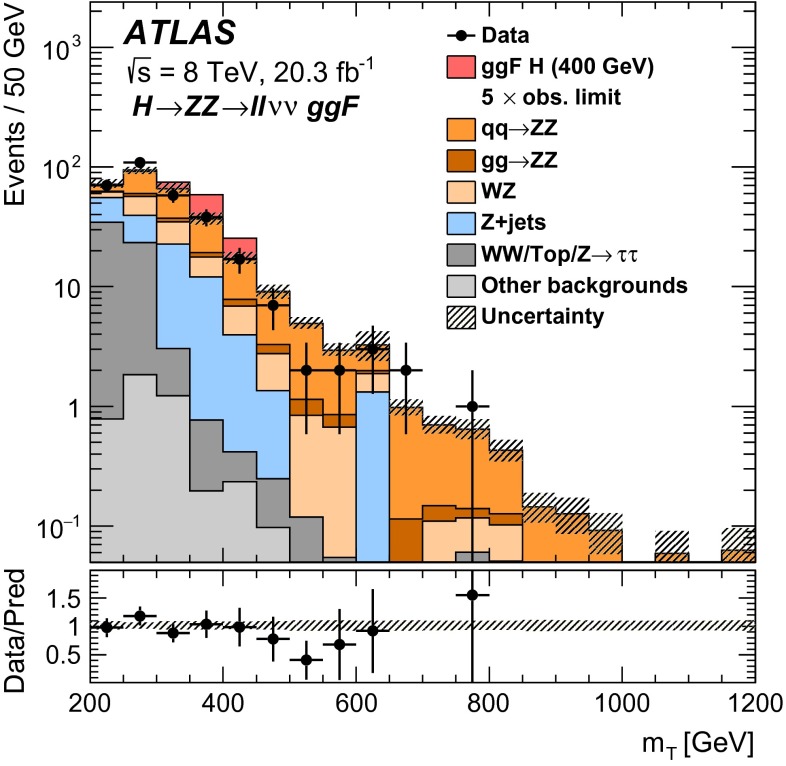
The distribution used in the likelihood fit of the transverse mass $m_{\mathrm {T}}^{ZZ}$ reconstructed from the momentum of the dilepton system and the missing transverse momentum for the $H\rightarrow ZZ\rightarrow \ell ^+\ell ^-\nu \bar{\nu }$ search in the ggF channel. The simulated signal is normalized to a cross-section corresponding to five times the observed limit given in Sect. 11. The contribution labelled as ‘Top’ includes both the $t\bar{t}$ and single-top processes. *The bottom pane* shows the ratio of the observed data to the predicted background

Figure 2 shows the $m_{\mathrm {T}}^{ZZ}$ distribution in the ggF channel. The event yields in the VBF channel are very small (see Table 2).

### Background estimation

The dominant background is $ZZ$ production, followed by $WZ$ production. Other important backgrounds to this search include the $WW$, $t\bar{t}$, $Wt$, and $Z\rightarrow \tau ^+\tau ^-$ processes, and also the $Z+\mathrm {jets}$ process with poorly reconstructed $E_{\text {T}}^{\text {miss}}$, but these processes tend to yield final states with low $m_{\mathrm {T}}$. Backgrounds from $W+\mathrm {jets}$, $t\bar{t}$, single top quark ($s$- and $t$-channel), and multijet processes with at least one jet misidentified as an electron or muon are very small.

The Powheg simulation is used to estimate the $ZZ$ background in the same way as for the $\ell \ell \ell \ell$ search. The $WZ$ background is also estimated with Powheg and validated with data using a sample of events that pass the signal selection and that contain an extra electron or muon in addition to the $Z\rightarrow \ell ^+\ell ^-$ candidate.

The $WW$, $t\bar{t}$, $Wt$, and $Z\rightarrow \tau ^+\tau ^-$ processes give rise to both same-flavour as well as different-flavour lepton final states. The total background from these processes in the same-flavour final state can be estimated from control samples that contain an electron–muon pair rather than a same-flavour lepton pair by

2 $$\begin{aligned} \begin{array}{ll} N^\mathrm{bkg}_{ee} =\frac{1}{2} \times N^\mathrm{data,sub}_{e\mu }\times {f}, \\ N^\mathrm{bkg}_{\mu \mu } = \frac{1}{2} \times N^\mathrm{data,sub}_{e\mu }\times \frac{1}{f}, \end{array} \end{aligned}$$

where $N^\mathrm{bkg}_{ee}$ and $N^\mathrm{bkg}_{\mu \mu }$ are the number of electron and muon pair events in the signal region and $N^\mathrm{data,sub}_{e\mu }$ is the number of events in the $e\mu$ control sample with $WZ$, $ZZ$, and other small backgrounds ($W+\mathrm {jets}$, $t\bar{t} W/Z$, and triboson) subtracted using simulation. The factor of two arises because the branching ratio to final states containing electrons and muons is twice that of either $ee$ or $\mu \mu$. The factor $f$ takes into account the different efficiencies for electrons and muons and is measured from data as $f^2 = N_{ee}^{\mathrm {data}} / N_{\mu \mu }^{\mathrm {data}}$, the ratio of the number of electron pair to muon pair events in the data after the $Z$ boson mass requirement ($76 < m_{\ell \ell } < 106~\mathrm{GeV}$). The measured value of $f$ is 0.94 with a systematic uncertainty of 0.04 and a negligible statistical uncertainty. There is also a systematic uncertainty from the background subtraction in the control sample; this is less than 1 %. For the VBF channel, no events remain in the $e\mu$ control sample after applying the full selection. In this case, the background estimate is calculated after only the requirements on $E_{\text {T}}^{\text {miss}}$ and the number of jets; the efficiencies of the remaining selections for this background are estimated using simulation.

The $Z+\mathrm {jets}$ background is estimated from data by comparing the signal region (A) with regions in which one (B, C) or both (D) of the $\Delta \phi _{\ell \ell }$ and $\Delta \phi (p_{\text {T}}^{\ell \ell },E_{\text {T}}^{\text {miss}})$ requirements are reversed. An estimate of the number of background events in the signal region is then $N_{\mathrm {A}}^{\mathrm {est}} = N_{\mathrm {C}}^{\mathrm {obs}}\times (N_{\mathrm {B}}^{\mathrm {obs}} / N_{\mathrm {D}}^{\mathrm {obs}})$, where $N_X^{\mathrm {obs}}$ is the number of events observed in region $X$ after subtracting non-$Z$ boson backgrounds. The shape is estimated by taking $N_C^{\mathrm {obs}}$ (the region with the $\Delta \phi _{\ell \ell }$ requirement reversed) bin-by-bin and applying a correction derived from MC simulations to account for shape differences between regions A and C. Systematic uncertainties arise from differences in the shape of the $E_{\text {T}}^{\text {miss}}$ and $m_{\mathrm {T}}^{ZZ}$ distributions among the four regions, the small correlation between the two variables, and the subtraction of non-$Z$ boson backgrounds.

The $W+\mathrm {jets}$ and multijet backgrounds are estimated from data using the fake-factor method [85]. This uses a control sample derived from data using a loosened requirement on $E_{\text {T}}^{\text {miss}}$ and several kinematic selections. The background in the signal region is then derived using an efficiency factor from simulation to correct for the acceptance. Both of these backgrounds are found to be negligible.

Table 2 shows the expected yields of the backgrounds and signal, and observed counts of data events. The expected yields of the backgrounds in the table are after applying the combined likelihood fit to the data, as explained in Sect. 10.

**Table 2:** Expected background yields and observed counts of data events after all selections for the ggF and VBF channels of the $H\rightarrow ZZ\rightarrow \ell ^+\ell ^-\nu \bar{\nu }$ search. The first and second uncertainties correspond to the statistical and systematic uncertainties, respectively

Process	ggF channel	VBF channel
$q\bar{q}\rightarrow ZZ$	$110 \pm 1\pm 10$	$0.13\pm 0.04\pm 0.02$
$gg\rightarrow ZZ$	$11\pm 0.1\pm 5$	$0.12\pm 0.01\pm 0.05$
$WZ$	$47\pm 1\pm 5$	$0.10\pm 0.05\pm 0.1$
$WW$/$t\bar{t}$/$Wt$/$Z\rightarrow \tau ^+ \tau ^-$	$58\pm 6\pm 5$	$0.41\pm 0.01\pm 0.08$
$Z(\rightarrow e^+e^-,\mu ^+\mu ^-)+$jets	$74\pm 7\pm 20$	$0.8\pm 0.3\pm 0.3$
Other backgrounds	$4.5\pm 0.7\pm 0.5$	–
Total background	$310\pm 9\pm 40$	$1.6\pm 0.3\pm 0.5$
Observed	309	4
ggF signal ($m_{H} =400~\mathrm{GeV}$)	$45\pm 1\pm 3$	–
VBF signal ($m_{H} =400~\mathrm{GeV}$)	$1 \pm {<}0.1\pm 2$	$10\pm 0.5\pm 1$

## $H\rightarrow ZZ\rightarrow \ell ^+\ell ^- q \bar{q}$ event selection and background estimation

### Event selection

As in the previous search, the event selection starts with the reconstruction of a $Z\rightarrow \ell \ell$ decay. For the purpose of this search, leptons are classified as either ‘loose’, with $p_{\text {T}} >7~\mathrm{GeV}$, or ‘tight’, with $p_{\text {T}} >25~\mathrm{GeV}$. Loose muons extend to $|\eta |<2.7$, while tight muons are restricted to $|\eta |<2.5$ and must have tracks in both the ID and the MS. The transverse impact parameter requirement for muons is tightened for this search to $|d_0|<0.1\mathrm{mm}$. Electrons are identified using a likelihood discriminant very similar to that used for the $\ell \ell \ell \ell$ search, except that it was tuned for a higher signal efficiency. This selection is denoted ‘very loose LH’ [73]. To avoid double counting, the following procedure is applied to loose leptons and jets. First, any jets that lie $\Delta R < 0.4$ of an electron are removed. Next, if a jet is within a cone of $\Delta R = 0.4$ of a muon, the jet is discarded if it has less than two matched tracks or if the JVF recalculated without muons (see Sect. 4) is less than 0.5, since in this case it is likely to originate from a muon having showered in the calorimeter; otherwise the muon is discarded. (Such muons are nevertheless included in the computation of the $E_{\text {T}}^{\text {miss}}$ and in the jet energy corrections described in Sect. 4.) Finally, if an electron is within a cone of $\Delta R = 0.2$ of a muon, the muon is kept unless it has no track in the MS, in which case the electron is kept.

Events must contain a same-flavour lepton pair with invariant mass satisfying $$83 < $$ $$m_{\ell \ell }$$ $$ < 99~\mathrm{GeV}$$. At least one of the leptons must be tight, while the other may be either tight or loose. Events containing any additional loose leptons are rejected. The two muons in a pair are required to have opposite charge, but this requirement is not imposed for electrons because larger energy losses from showering in material in the inner tracking detector lead to higher charge misidentification probabilities.

Jets used in this search to reconstruct the $Z \rightarrow q\bar{q}$ decay, referred to as ‘signal’ jets, must have $|\eta |<2.5$ and $p_{\text {T}} >20~\mathrm{GeV}$; the leading signal jet must also have $p_{\text {T}} >45~\mathrm{GeV}$. The search for forward jets in the VBF production mode uses an alternative, ‘loose’, jet definition, which includes both signal jets and any additional jets satisfying $2.5<|\eta |<4.5$ and $p_{\text {T}} >30~\mathrm{GeV}$. Since no high-$p_{\text {T}}$ neutrinos are expected in this search, the significance of the missing transverse momentum, $E_{\text {T}}^{\text {miss}}/ \sqrt{H_{\text {T}}}$ (all quantities in GeV), where $H_{\text {T}}$ is the scalar sum of the transverse momenta of the leptons and loose jets, must be less than 3.5. This requirement is loosened to 6.0 for the case of the resolved channel (see Sect. 7.1.1) with two $b$-tagged jets due to the presence of neutrinos from heavy-flavour decay. The $E_{\text {T}}^{\text {miss}}$ significance requirement rejects mainly top-quark background.

Following the selection of the $Z\rightarrow \ell \ell$ decay, the search is divided into several channels: resolved ggF, merged-jet ggF, and VBF, as discussed below.

#### Resolved ggF channel

Over most of the mass range considered in this search ($m_{H} \lesssim 700~\mathrm{GeV}$), the $Z \rightarrow q\bar{q}$ decay results in two well-separated jets that can be individually resolved. Events in this channel should thus contain at least two signal jets. Since $b$-jets occur much more often in the signal (${\sim }21~\%$ of the time) than in the dominant $Z+\mathrm {jets}$ background (${\sim }2~\%$ of the time), the sensitivity of this search is optimized by dividing it into ‘tagged’ and ‘untagged’ subchannels, containing events with exactly two and fewer than two $b$-tagged jets, respectively. Events with more than two $b$-tagged jets are rejected.

In the tagged subchannel, the two $$b$$-tagged jets form the candidate $$Z \rightarrow q\bar{q} $$ decay. In the untagged subchannel, if there are no $$b$$-tagged jets, the two jets with largest transverse momenta are used. Otherwise, the $$b$$-tagged jet is paired with the non-$$b$$-tagged jet with the largest transverse momentum. The invariant mass of the chosen jet pair $$m_{jj} $$ must be in the range $$70$$–$$105~\mathrm{GeV}$$ in order to be consistent with $$Z \rightarrow q\bar{q} $$ decay. To maintain orthogonality, any events containing a VBF-jet pair as defined by the VBF channel (see Sect. 7.1.3) are excluded from the resolved selection.

The discriminating variable in this search is the invariant mass of the $$\ell \ell jj $$ system, $$m_{\ell \ell jj} $$; a signal should appear as a peak in this distribution. To improve the mass resolution, the energies of the jets forming the dijet pair are scaled event-by-event by a single multiplicative factor to set the dijet invariant mass $$m_{jj} $$ to the mass of the $$Z$$ boson ($$m_Z$$). This improves the resolution by a factor of 2.4 at $$m_{H} =200~\mathrm{GeV}$$. The resulting $$m_{\ell \ell jj} $$ resolution is 2–3 %, approximately independent of $$m_{H} $$, for both the untagged and tagged channels.

**Fig. 3:**
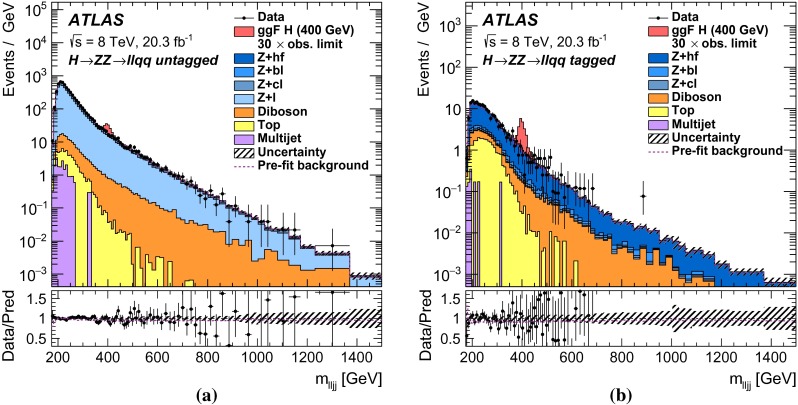
The distributions used in the likelihood fit of the invariant mass of dilepton $+$ dijet system $m_{\ell \ell jj}$ for the $H\rightarrow ZZ\rightarrow \ell ^+\ell ^- q \bar{q}$ search in the **a** untagged and **b** tagged resolved ggF subchannels. *The dashed line* shows the total background used as input to the fit. The simulated signal is normalized to a cross-section corresponding to 30 times the observed limit given in Sect. 11. The contribution labelled as ‘Top’ includes both the $t\bar{t}$ and single-top processes. *The bottom panes* show the ratio of the observed data to the predicted background

Following the selection of the candidate $$\ell \ell qq $$ decay, further requirements are applied in order to optimize the sensitivity of the search. For the untagged subchannel, the first requirement is on the transverse momentum of the leading jet, $$p_{\text {T}}^{j} $$, which tends to be higher for the signal than for the background. The optimal value for this requirement increases with increasing $$m_{H} $$. In order to avoid having distinct selections for different $$m_{H} $$ regions, $$p_{\text {T}}^{j} $$ is normalized by the reconstructed final-state mass $$m_{\ell \ell jj} $$; the actual selection is $$p_{\text {T}}^{j} > 0.1\times m_{\ell \ell jj} $$. Studies have shown that the optimal requirement on $$p_{\text {T}}^{j}/m_{\ell \ell jj} $$ is nearly independent of the assumed value of $$m_{H} $$. Second, the total transverse momentum of the dilepton pair also increases with increasing $$m_{H} $$. Following a similar strategy, the selection is $$p_{\text {T}}^{\ell \ell } > \min [-54 ~\mathrm{GeV}+ 0.46\times m_{\ell \ell jj}, 275~\mathrm{GeV}]$$. Finally, the azimuthal angle between the two leptons decreases with increasing $$m_{H} $$; it must satisfy $$\Delta \phi _{\ell \ell } < (270~\mathrm{GeV}/m_{\ell \ell jj})^{3.5} + 1$$. For the tagged channel, only one additional requirement is applied: $$p_{\text {T}}^{\ell \ell } > \min [-79~\mathrm{GeV}+ 0.44\times m_{\ell \ell jj}, 275~\mathrm{GeV}]$$; the different selection for $$p_{\text {T}}^{\ell \ell } $$ increases the sensitivity of the tagged channel at low $$m_{H} $$. Figure 3a and b show the $$m_{\ell \ell jj} $$ distributions of the two subchannels after the final selection.

#### Merged-jet ggF channel

For very large Higgs boson masses, $m_{H} \gtrsim 700~\mathrm{GeV}$, the $Z$ bosons become highly boosted and the jets from $Z \rightarrow q\bar{q}$ decay start to overlap, causing the resolved channel to lose efficiency. The merged-jet channel recovers some of this loss by looking for a $Z \rightarrow q\bar{q}$ decay that is reconstructed as a single jet.

Events are considered for the merged-jet channel if they have exactly one signal jet, or if the selected jet pair has an invariant mass outside the range $50$–$150~\mathrm{GeV}$ (encompassing both the signal region and the control regions used for studying the background). Thus, the merged-jet channel is explicitly orthogonal to the resolved channel.

To be considered for the merged-jet channel, the dilepton pair must have $p_{\text {T}}^{\ell \ell } >280~\mathrm{GeV}$. The leading jet must also satisfy $p_{\text {T}} > 200~\mathrm{GeV}$ and $m / p_{\text {T}} > 0.05$, where $m$ is the jet mass, in order to restrict the jet to the kinematic range in which the mass calibration has been studied. Finally, the invariant mass of the leading jet must be within the range $70$–$105~\mathrm{GeV}$. The merged-jet channel is not split into subchannels based on the number of $b$-tagged jets; as the sample size is small, this would not improve the expected significance.

**Fig. 4:**
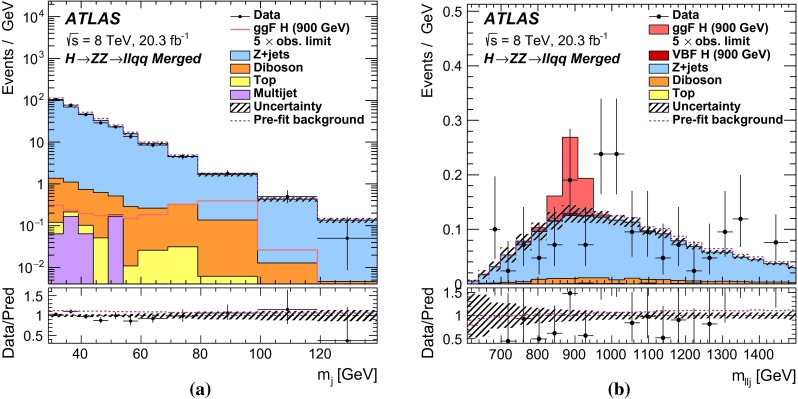
Distributions for the merged-jet channel of the $$H\rightarrow ZZ\rightarrow \ell ^+\ell ^- q \bar{q} $$ search after the mass calibration. **a** The invariant mass of the leading jet, $$m_j $$, after the kinematic selection for the $$\ell \ell qq $$ merged-jet channel. **b** The distribution used in the likelihood fit of the invariant mass of the two leptons and the leading jet $$m_{\ell \ell j} $$ in the signal region. It is obtained requiring $$70 < m_j < 105~\mathrm{GeV}$$. *The dashed line* shows the total background used as input to the fit. The simulated signal is normalized to a cross-section corresponding to five times the observed limit given in Sect. 11. The contribution labelled as ‘Top’ includes both the $$t\bar{t} $$ and single-top processes. *The bottom panes* show the ratio of the observed data to the predicted background. The signal contribution is shown added on *top* of the background in **b** but not in **a**

Including this channel increases the overall efficiency for the $\ell \ell qq$ signal at $m_{H} =900~\mathrm{GeV}$ by about a factor of two. Figure 4a shows the distribution of the invariant mass of the leading jet after all selections except for that on the jet invariant mass; it can be seen that the simulated signal has a peak at the mass of the $Z$ boson, with a tail at lower masses due to events where the decay products of the $Z$ boson are not fully contained in the jet cone. The discriminating variable for this channel is the invariant mass of the two leptons plus the leading jet, $m_{\ell \ell j}$, which has a resolution of 2.5 % for a signal with $m_{H} =900~\mathrm{GeV}$ and is shown in Fig. 4b.

#### VBF channel

**Fig. 5:**
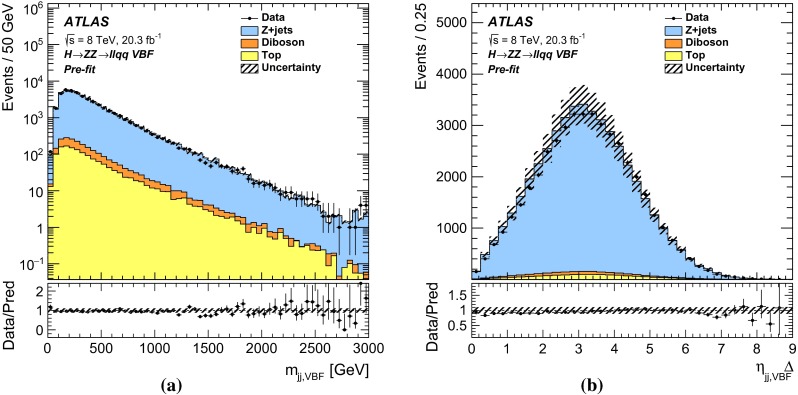
Distribution of **a** invariant mass and **b** pseudorapidity gap for the VBF-jet pair in the VBF channel of the $H\rightarrow ZZ\rightarrow \ell ^+\ell ^- q \bar{q}$ search before applying the requirements on these variables (and prior to the combined fit described in Sect. 10). The contribution labelled as ‘Top’ includes both the $t\bar{t}$ and single-top processes. *The bottom panes* show the ratio of the observed data to the predicted background

Events produced via the VBF process contain two forward jets in addition to the reconstructed leptons and signal jets from $ZZ\rightarrow \ell ^+\ell ^- q \bar{q}$ decay. These forward jets are called ‘VBF jets’. The search in the VBF channel starts by identifying a candidate VBF-jet pair. Events must have at least four loose jets, two of them being non-$b$-tagged and pointing in opposite directions in $z$ (that is, $\eta _1\cdot \eta _2 < 0$). If more than one such pair is found, the one with the largest invariant mass, $m_{jj,\mathrm{VBF}}$, is selected. The pair must further satisfy $m_{jj,\mathrm{VBF}} > 500~\mathrm{GeV}$ and have a pseudorapidity gap of $|\Delta \eta _{jj,\mathrm{VBF}}| > 4$. The distributions of these two variables are shown in Fig. 5.

**Fig. 6:**
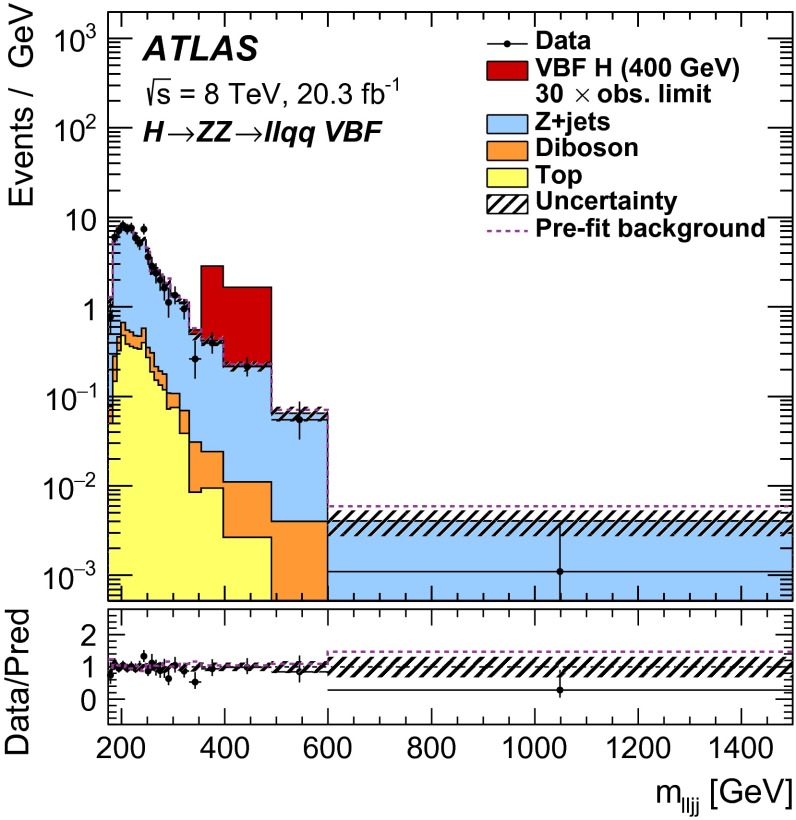
The distribution of $m_{\ell \ell jj}$ used in the likelihood fit for the $H\rightarrow ZZ\rightarrow \ell ^+\ell ^- q \bar{q}$ search in the VBF channel. *The dashed line* shows the total background used as input to the fit. The simulated signal is normalized to a cross-section corresponding to 30 times the observed limit given in Sect. 11. The contribution labelled as ‘Top’ includes both the $t\bar{t}$ and single-top processes. *The bottom pane* shows the ratio of the observed data to the predicted background

Once a VBF-jet pair has been identified, the $ZZ\rightarrow \ell ^+\ell ^- q \bar{q}$ decay is reconstructed in exactly the same way as in the resolved channel, except that the jets used for the VBF-jet pair are excluded and no $b$-tagging categories are created due to the small sample size. The final $m_{\ell \ell jj}$ discriminant is shown in Fig. 6. Again, the resolution is improved by constraining the dijet mass to $m_Z$ as described in Sect. 7.1.1, resulting in a similar overall resolution of 2–3 %.

### Background estimation

The main background in the $\ell \ell qq$ search is $Z+\mathrm {jets}$ production, with significant contributions from both top-quark and diboson production in the resolved ggF channel, as well as a small contribution from multijet production in all channels. For the multijet background, the shape and normalization is taken purely from data, as described below. For the other background processes, the input is taken from simulation, with data-driven corrections for $Z+\mathrm {jets}$ and $t\bar{t}$ production. The normalizations of the $Z+\mathrm {jets}$ and top-quark backgrounds are left free to float and are determined in the final likelihood fit as described below and in Sect. 10.

The $$Z+\mathrm {jets} $$ MC sample is constrained using control regions that have the same selection as the signal regions except that $$m_{jj} $$ ($$m_j $$ in the case of the merged-jet channel) lies in a region just outside of that selected by the signal $$Z$$ boson requirement. For the resolved channels, the requirement for the control region is $$50<m_{jj} <70~\mathrm{GeV}$$ or $$105<m_{jj} <150~\mathrm{GeV}$$; for the merged-jet channel, it is $$30<m_j <70~\mathrm{GeV}$$. In the resolved ggF channel, which is split into untagged and tagged subchannels as described in Sect. 7.1.1, the $$Z+\mathrm {jets} $$ control region is further subdivided into 0-tag, 1-tag, and 2-tag subchannels based on the number of $$b$$-tagged jets. The sum of the 0-tag and 1-tag subchannels is referred to as the untagged control region, while the 2-tag subchannel is referred to as the tagged control region.

The normalization of the $Z+\mathrm {jets}$ background is determined by the final profile-likelihood fit as described in Sect. 10. In the resolved ggF channel, the simulated $Z+\mathrm {jets}$ sample is split into several different components according to the true flavour of the jets as described in Sect. 3.3: $Z+jj$, $Z+cj$, $Z+bj$, and $Z+$hf. The individual normalizations for each of these four components are free to float in the fit and are constrained by providing as input to the fit the distribution of the “$b$-tagging category” in the untagged and tagged $Z+\mathrm {jets}$ control regions. The $b$-tagging category is defined by the combination of the MV1c $b$-tagging discriminants of the two signal jets as described in Appendix A. In the VBF and merged-jet ggF channels, which are not divided into $b$-tag subchannels, the background is dominated by $Z+$light-jets. Thus, only the inclusive $Z+\mathrm {jets}$ normalization is varied in the fit for these channels. Since these two channels probe very different regions of phase space, each has a separate normalization factor in the fit; these are constrained by providing to the fit the distributions of $m_{\ell \ell jj}$ or $m_{\ell \ell j}$ for the corresponding $Z+\mathrm {jets}$ control regions.

**Fig. 7:**
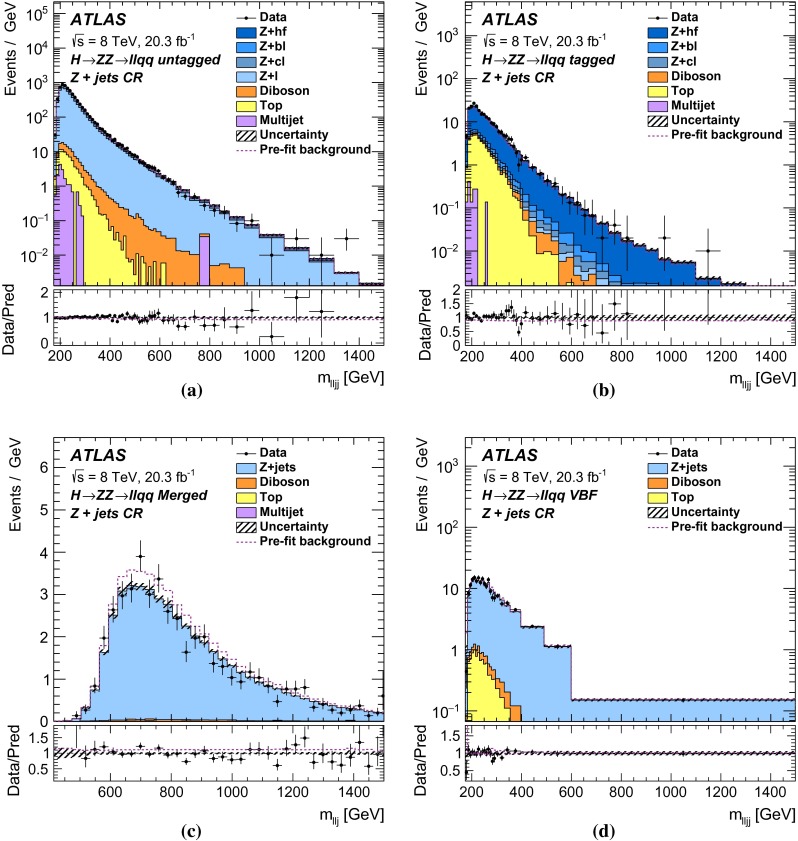
The distributions of $m_{\ell \ell jj}$ or $m_{\ell \ell j}$ in the $Z+\mathrm {jets}$ control region of the $H\rightarrow ZZ\rightarrow \ell ^+\ell ^- q \bar{q}$ search in the **a** untagged ggF, **b** tagged ggF, **c** merged-jet ggF, and **d** VBF channels. *The dashed line* shows the total background used as input to the fit. The contribution labelled as ‘Top’ includes both the $t\bar{t}$ and single-top processes. *The bottom panes* show the ratio of the observed data to the predicted background

Differences are observed between data and MC simulation for the distributions of the azimuthal angle between the two signal jets, $\Delta \phi _{jj}$, and the transverse momentum of the leptonically-decaying $Z$ boson, $p_{\text {T}}^{\ell \ell }$, for the resolved region, and for the $m_{\ell \ell jj}$ distribution in the VBF channel. To correct for these differences, corrections are applied to the Sherpa $Z+\mathrm {jets}$ simulation (prior to the likelihood fit) as described in Appendix B. The distributions of $m_{\ell \ell jj}$ or $m_{\ell \ell j}$ in the various $Z+\mathrm {jets}$ control regions are shown in Fig. 7; it can be seen that after the corrections (and after normalizing to the results of the likelihood fit), the simulation provides a good description of the data.

The simulation models the $$m_{jj} $$ distribution well in the resolved ggF and VBF channels. An uncertainty is assigned by weighting each event of the $$Z+\mathrm {jets} $$ MC simulation by a linear function of $$m_{jj} $$ in order to cover the residual difference between data and MC events in the control regions.

**Fig. 8:**
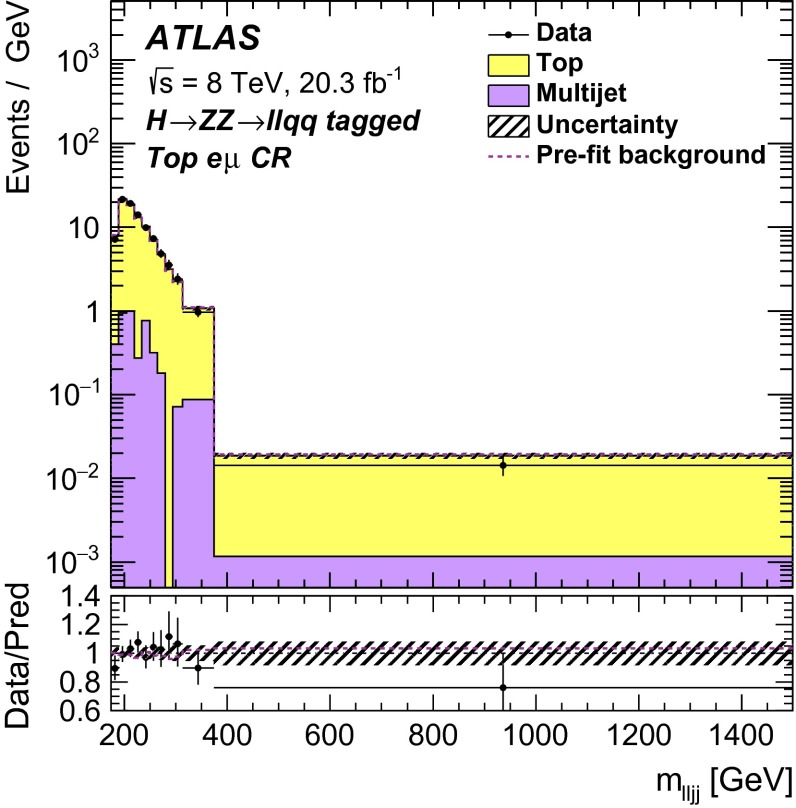
The distribution of $m_{\ell \ell jj}$ in the $e\mu$ top-quark control region of the $H\rightarrow ZZ\rightarrow \ell ^+\ell ^- q \bar{q}$ search in the tagged ggF channel. *The dashed line* shows the total background used as input to the fit. The contribution labelled as ‘Top’ includes both the $t\bar{t}$ and single-top processes. *The bottom pane* shows the ratio of the observed data to the predicted background

Top-quark production is a significant background in the tagged subchannel of the resolved ggF channel. This background is predominantly (${>}97~\%$) $t\bar{t}$ production with only a small contribution from single-top processes, mainly $Wt$ production. Corrections to the simulation to account for discrepancies in the $p_{\text {T}}^{t\bar{t}}$ distributions are described in Appendix B. The description of the top-quark background is cross-checked and normalized using a control region with a selection identical to that of the tagged ggF channel except that instead of two same-flavour leptons, events must contain an electron and a muon with opposite charge. The $m_{\ell \ell jj}$ distribution in this control region is used as an input to the final profile-likelihood fit, in which the normalization of the top-quark background is left free to float (see Sect. 10). There are few events in the control region for the VBF and merged-jet ggF channels, so the normalization is assumed to be the same across all channels, in which the top-quark contribution to the background is very small. Figure 8 shows that the data in the control region are well-described by the simulation after the normalization.

Further uncertainties in the top-quark background arising from the parton showering and hadronization models are estimated by varying the amount of parton showering in AcerMC and also by comparing with Powheg+Herwig. Uncertainties in the $$t\bar{t} $$ production matrix element are estimated by comparing the leading-order MC generator Alpgen with the NLO generator aMC@NLO. Comparisons are also made with alternate PDF sets. A similar procedure is used for single-top production. In addition, for the dominant $$Wt$$ single-top channel, uncertainties in the shapes of the $$m_{jj} $$ and leading-jet $$p_{\text {T}} $$ distributions are evaluated by comparing results from Herwig to those from AcerMC.

The small multijet background in the $H \rightarrow ZZ \rightarrow eeqq$ decay mode is estimated from data by selecting a sample of events with the electron isolation requirement inverted, which is then normalized by fitting the $m_{ee}$ distribution in each channel. In the $H \rightarrow ZZ \rightarrow \mu \mu qq$ decay mode, the multijet background is found to be negligible. The residual multijet background in the top-quark control region is taken from the opposite-charge $e\mu$ data events, which also accounts for the small $W+\mathrm {jets}$ background in that region. An uncertainty of 50 % is assigned to these two normalizations, which are taken to be uncorrelated.

The diboson background, composed mainly of $$ZZ$$ and $$WZ \rightarrow \ell \ell jj$$ production, and the SM $$Zh \rightarrow \ell \ell bb$$ background are taken directly from Monte Carlo simulation, as described in Sect. 3.3. The uncertainty in the diboson background is estimated by varying the factorization and renormalization scales in an MCFM calculation [35]. The method described in Refs. [86, 87] is used to avoid underestimating the uncertainty due to cancellations. Differences due to the choice of alternate PDF sets and variations in the value of $$\alpha _{\text {S}} $$ are included in the normalization uncertainty. Additional shape uncertainties in the $$m_{jj} $$ distribution are obtained by comparing results from Herwig, an LO simulation, with those from Powheg+Pythia, an NLO simulation.

The rate of the SM $Vh (V=W/Z, h\rightarrow bb)$ process, relative to the SM expectation, has been measured by ATLAS as $\mu = \sigma /\sigma _{SM} = 0.52 \pm 0.32~\mathrm{(stat.)} \pm 0.24~\mathrm{(syst.)}$ [30]. Since this is compatible with the SM expectation, the small $Zh (h\rightarrow bb)$ background in this channel is normalized to the SM cross-section and a 50 % uncertainty is assigned to cover the difference between the prediction and the measured mean value.

## $H\rightarrow ZZ\rightarrow \nu \bar{\nu } q \bar{q}$ event selection and background estimation

### Event selection

Events selected for this search must contain no electrons or muons as defined by the ‘loose’ lepton selection of the $\ell \ell qq$ search. To select events with neutrinos in the final state, the magnitude of the missing transverse momentum vector must satisfy $E_{\text {T}}^{\text {miss}} >160~\mathrm{GeV}$; the trigger is 100 % efficient in this range. Events must have at least two jets with $p_{\text {T}} >20~\mathrm{GeV}$ and $|\eta |<2.5$; the leading jet must further satisfy $p_{\text {T}} >45~\mathrm{GeV}$. To select a candidate $Z \rightarrow q\bar{q}$ decay, the invariant mass of the leading two jets must satisfy $70<m_{jj} <105~\mathrm{GeV}$.

The multijet background, due mainly to the mismeasurement of jet energies, is suppressed using a track-based missing transverse momentum, ${{\vec {}}{p_{\text {T}} ^{\mathrm {miss}} }}$, defined as the negative vectorial sum of the transverse momenta of all good-quality inner detector tracks. The requirements are $p_{\text {T}} ^{\mathrm {miss}}$ ${>}30~\mathrm{GeV}$, the azimuthal angle between the directions of ${{\vec {}}{E_{\text {T}}^{\text {miss}} }}$ and ${{\vec {}}{p_{\text {T}} ^{\mathrm {miss}} }}$ satisfy $\Delta \phi   {{\vec {}}{E_{\text {T}}^{\text {miss}} }},  {{\vec {}}{p_{\text {T}} ^{\mathrm {miss}} }})<\pi /2$, and the azimuthal angle between the directions of ${{\vec {}}{E_{\text {T}}^{\text {miss}} }}$ and the nearest jet satisfy $\Delta \phi ( {{\vec {}}{E_{\text {T}}^{\text {miss}} }}, j)>0.6$.

As in the resolved ggF channel of the $\ell \ell qq$ search, this search is divided into ‘tagged’ (exactly two $b$-tagged jets) and ‘untagged’ (fewer than two $b$-tagged jets) subchannels. Events with more than two $b$-tags are rejected.

The sensitivity of this search is improved by adding a requirement on the jet transverse momenta. As in the $$\ell \ell qq $$ search, the optimal threshold depends on $$m_{H} $$. However, due to the neutrinos in the final state, this decay mode does not provide a good event-by-event measurement of the mass of the diboson system, $$m_{ZZ} $$. So, rather than having a single requirement on the jet transverse energy which is a function of the measured $$m_{ZZ} $$, instead there is a set of requirements, based on the generated $$m_{H} $$, with the background estimated separately for each of these separate jet requirements. The specific requirement is found by rounding the generated $$m_{H} $$ to the nearest $$100~\mathrm{GeV}$$; this is called $$m_{H} ^\mathrm {bin} $$. Then the subleading jet must satisfy $$p_{\text {T}}^{j2} > 0.1\times m_{H} ^\mathrm {bin} $$ in events with no $$b$$-tagged jets, and $$p_{\text {T}}^{j2} > 0.1\times m_{H} ^\mathrm {bin}- 10~\mathrm{GeV}$$ in events with at least one $$b$$-tagged jet.

**Fig. 9:**
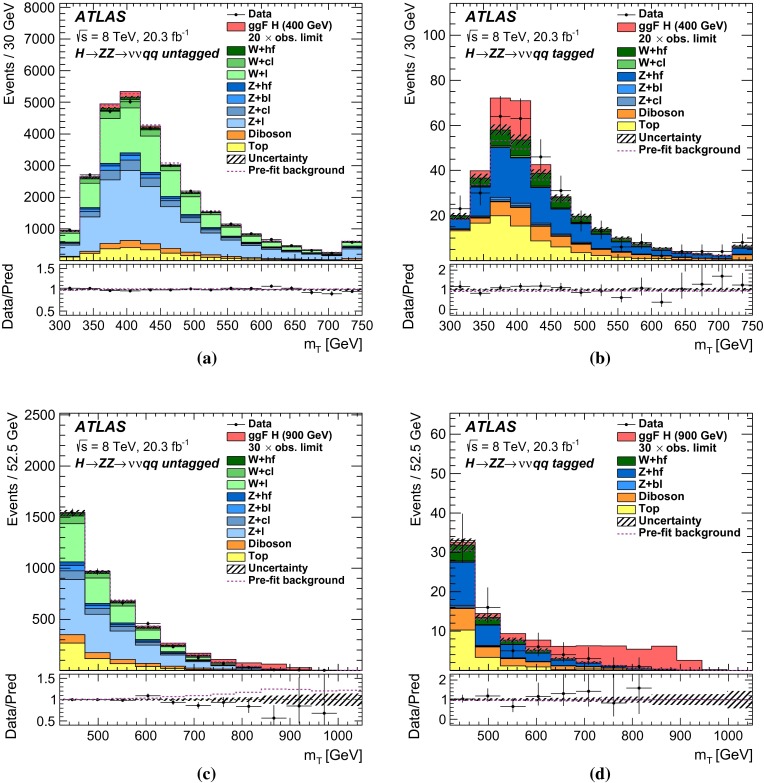
The distributions of $m_{\mathrm {T}}$, the transverse mass of the $Z(\nu \nu )Z(jj)$ system, used in the likelihood fit for the $H\rightarrow ZZ\rightarrow \nu \bar{\nu } q \bar{q}$ search in the **a**, **c** untagged and **b**, **d** tagged channels, for Higgs boson mass hypotheses of **a**, **b** $m_{H} =400~\mathrm{GeV}$ and **c**, **d** $m_{H} =900~\mathrm{GeV}$. *The dashed line* shows the total background used as input to the fit. For the $m_{H} =400~\mathrm{GeV}$ hypothesis (**a**, **b**) the simulated signal is normalized to a cross-section corresponding to 20 times the observed limit given in Sect. 11, while for the $m_{H} =900~\mathrm{GeV}$ hypothesis (**c**, **d**) it is normalized to 30 times the observed limit. The contribution labelled as ‘Top’ includes both the $t\bar{t}$ and single-top processes. *The bottom panes* show the ratio of the observed data to the predicted background

The discriminating variable for this search is the transverse mass of the $$\nu \nu qq $$ system, shown in Fig. 9, defined as in Eq. (1) with $$p_{\text {T}}^{jj} $$ replacing $$p_{\text {T}}^{\ell \ell } $$. To improve the transverse mass resolution, the energies of the leading two jets are scaled event-by-event by a multiplicative factor to set the dijet invariant mass $$m_{jj} $$ to the $$Z$$ boson mass, in the same manner as in the $$\ell \ell qq$$ search. This improves the transverse mass resolution by approximately 20 % at $$m_{H} =400$$ GeV and by approximately 10 % at $$m_{H} =1$$ TeV. The resulting resolution in $$m_{\mathrm {T}} $$ ranges from about 9 % at $$m_{H} =400~\mathrm{GeV}$$ to 14 % at $$m_{H} =1~\mathrm{TeV}$$.

### Background estimation

The dominant backgrounds for this search are $Z+\mathrm {jets}$, $W+\mathrm {jets}$, and $t\bar{t}$ production. The normalization of the $Z+\mathrm {jets}$ background is determined using the $Z+\mathrm {jets}$ control region from the $\ell \ell qq$ channel in the final profile-likelihood fit as described in Sect. 10. To check how well this background is modelled after the $\nu \nu qq$ selection, an alternative $Z+\mathrm {jets}$ control region is defined in the same way as the signal sample for $m_{H} ^\mathrm {bin} = 400$ GeV except that events must contain exactly two loose muons. The $E_{\text {T}}^{\text {miss}}$ is calculated without including the muons and must satisfy the same requirement as for the signal: $E_{\mathrm {T}}^{\mathrm {miss~no~\mu }} >160~\mathrm{GeV}$. The $Z+\mathrm {jets}$ MC simulation is corrected as a function of $\Delta \phi _{jj}$ and $p_{\text {T}}^{\ell \ell }$ in the same manner as in the resolved ggF channel of the $\ell \ell qq$ search, as described in Sect. 7.2 and Appendix B.

The $W+\mathrm {jets}$ background estimate similarly uses a control sample with the same selection as the signal sample for $m_{H} ^\mathrm {bin} = 400$ GeV except that there must be exactly one loose muon and the $E_{\text {T}}^{\text {miss}}$ requirement is again on $E_{\mathrm {T}}^{\mathrm {miss~no~\mu }}$. The simulated $W+\mathrm {jets}$ sample is also split into several different flavour components, as in the case of $Z+\mathrm {jets}$. The normalization of the $W+jj$ and $W+cj$ components are free to float in the final profile-likelihood fit, and are constrained by providing as input to the fit the distribution of the MV1c $b$-tagging category, described in Appendix A, in the 0-$b$-tag and 1-$b$-tag control regions. Unlike the $Z+\mathrm {jets}$ case, the 2-$b$-tag control region is not used in the final profile-likelihood fit to constrain the $W+bj$ and $W+$hf background components since it is highly dominated by $t\bar{t}$ production. Their normalizations are instead taken from the NNLO cross section predictions with an uncertainty of 50 %. The uncertainty is determined by comparing the nominal fit value from the profile-likelihood fit with the value when including the 2-$b$-tag control region, where $W+bj$ and $W+$hf are free to float; this uncertainty also covers the normalization determined in Ref. [30]. Following Ref. [30], the agreement between simulation and data for this background is improved by applying a correction to $\Delta \phi _{jj}$ for $W+jj$ and $W+cj$, with half the correction assigned as a systematic uncertainty; in the case of $W+bj$ and $W+$hf, no correction is applied, but a dedicated systematic uncertainty is assigned.

**Fig. 10:**
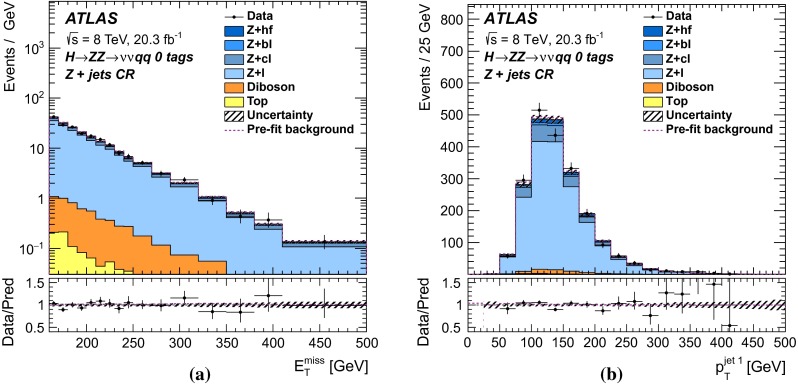
The distributions of **a** missing transverse momentum $E_{\text {T}}^{\text {miss}}$ and **b** leading-jet $p_{\text {T}}$ from the untagged $(Z\rightarrow \mu \mu ) + \mathrm {jets}$ control sample of the $H\rightarrow ZZ\rightarrow \nu \bar{\nu } q \bar{q}$ search. *The dashed line* shows the total background used as input to the fit. The contribution labelled as ‘Top’ includes both the $t\bar{t}$ and single-top processes. *The bottom panes* show the ratio of the observed data to the predicted background

**Fig. 11:**
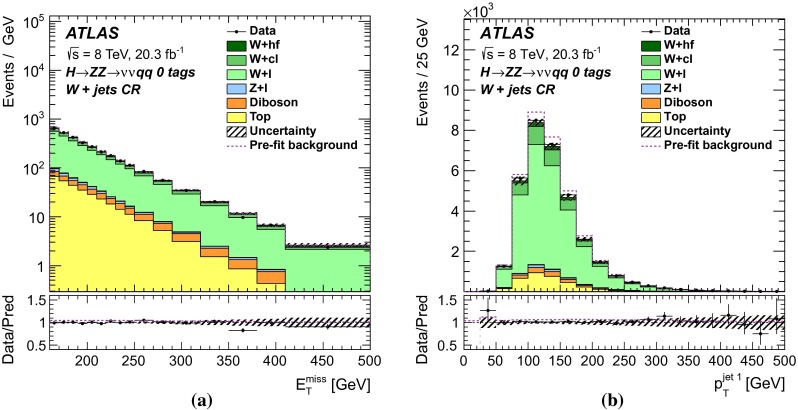
The distributions of **a** $E_{\text {T}}^{\text {miss}}$ and **b** leading-jet $p_{\text {T}}$ from the untagged $(W\rightarrow \mu \nu ) + \mathrm {jets}$ control sample of the $H\rightarrow ZZ\rightarrow \nu \bar{\nu } q \bar{q}$ search. *The dashed line* shows the total background used as input to the fit. The contribution labelled as ‘Top’ includes both the $t\bar{t}$ and single-top processes. *The bottom panes* show the ratio of the observed data to the predicted background

Even after these corrections, the simulation does not accurately describe the data in the $Z+\mathrm {jets}$ and $W+\mathrm {jets}$ control sample with no $b$-tagged jets (which is dominated by $Z/W+jj$) for important kinematic distributions such as $E_{\text {T}}^{\text {miss}}$ and jet transverse momenta. Moreover, because the resolution of the transverse mass of the $ZZ\rightarrow \nu \bar{\nu } q \bar{q}$ system is worse than that of $m_{\ell \ell jj}$, the $\nu \nu qq$ search is more sensitive to $E_{\text {T}}^{\text {miss}}$ (i.e. $Z/W$ boson $p_{\text {T}}$) than the $\ell \ell qq$ search. Therefore, a further correction is applied, as a linear function of $E_{\text {T}}^{\text {miss}}$, derived from measuring the ratio of the $E_{\text {T}}^{\text {miss}}$ distributions from simulation and data in the control sample with no $b$-tagged jets after non-$Z/W+jj$ backgrounds have been subtracted. An uncertainty of 50 % is assigned to this correction. Following this correction, there is good agreement between simulation and data, as shown in Figs. 10 and 11. For higher $m_{H} ^\mathrm {bin}$ signal samples, which have tighter selections on kinematic variables than the control sample, the $E_{\text {T}}^{\text {miss}}$ correction is somewhat underestimated, leading to some remaining difference between data and pre-fit simulation at high $m_T$, as can be seen in Fig. 9c. However, the profile-likelihood-ratio fit (Sect. 10) is able to correct this residual mismodelling, leading to reasonable agreement between the data and simulation.

The $t\bar{t}$ background is treated in the same manner as in the $\ell \ell qq$ search; in particular, $p_{\text {T}}^{t\bar{t}}$ is corrected in the same way and the normalization is determined by $t\bar{t}$ control region from $\ell \ell qq$ channel in the final profile-likelihood fit.

Backgrounds from diboson and single-top production are estimated directly from MC simulations, both for shapes and normalization. The multijet background is estimated using a method similar to that used for the $Z+\mathrm {jets}$ background in the $\ell \ell \nu \nu$ search (Sect. 6.2), except that the variables used are $\Delta \phi ( {{\vec {}}{E_{\text {T}}^{\text {miss}} }}, {{\vec {}}{p_{\text {T}} ^{\mathrm {miss}} }})$ and $\Delta \phi ( {{\vec {}}{E_{\text {T}}^{\text {miss}} }}, j)$ [30]. It is found to be negligible.

## Systematic uncertainties

The systematic uncertainties can be divided into three categories: experimental uncertainties, related to the detector or to the reconstruction algorithms, uncertainties in the modelling of the signal, and uncertainties in the estimation of the backgrounds. The first two are largely common to all the searches and are treated as fully correlated. The uncertainties in the estimates of most backgrounds vary from search to search, and are summarized in the background estimation sections above. The estimation of the uncertainty of the $$ZZ^{(*)} $$ background is outlined in Sect. 9.3.

### Experimental uncertainties

The following detector-related systematic uncertainties are common to all the searches unless otherwise stated.

The uncertainty in the integrated luminosity is determined to be 2.8 % in a calibration following the methodology detailed in Ref. [88] using beam-separation scans performed in November 2012. This uncertainty is applied to the normalization of the signal and also to backgrounds for which the normalization is derived from MC calculations, and is correlated between all of the searches. There is also an uncertainty of 4 % in the average number of interactions per bunch crossing, which leads to an uncertainty on distributions sensitive to pile-up.

There are small systematic uncertainties of $O(1~\%)$ in the reconstruction and identification efficiencies for electrons and muons [70–73]. For the $\nu \nu qq$ search, the uncertainty is instead in the efficiency of the lepton veto, and is also $O(1~\%)$. Uncertainties in the lepton energy scale and resolution are also taken into account. These uncertainties are treated as uncorrelated between all of the searches due to differences in lepton selections optimized for each search.

The uncertainty in the jet energy scale has several sources, including uncertainties in the in situ calibration analysis, corrections for pile-up, and the flavour composition of the sample [76, 89]. These uncertainties are decomposed into independent components. For central jets, the total relative uncertainty on the jet energy scale ranges from about 3 % for jets with a $p_{\text {T}}$ of $20~\mathrm{GeV}$ to about 1 % for a $p_{\text {T}}$ of $1~\mathrm{TeV}$. The calibration of the $b$-jet transverse energy has an additional uncertainty of 1–2 %. There is also an uncertainty in the jet energy resolution [90], which ranges from 10–20 % for jets with a $p_{\text {T}}$ of $20~\mathrm{GeV}$ to less than 5 % for jets with $p_{\text {T}} >200~\mathrm{GeV}$. The uncertainty associated with the pile-up rejection requirement (Sect. 4) is evaluated by varying the nominal value of 50 % between 47 and 53 % [78]. The jet energy scale uncertainties are correlated between the $\ell \ell qq$ and $\nu \nu qq$ searches, and separately between the $\ell \ell \ell \ell$ and $\ell \ell \nu \nu$ searches. They are not correlated between the two pairs of searches because although the $\ell \ell qq$ and $\nu \nu qq$ control regions have the power to constrain the jet energy scale uncertainties, these constraints do not necessarily apply to the $\ell \ell \ell \ell$ and $\ell \ell \nu \nu$ searches due to differences in the jet kinematics and composition.

Uncertainties on the lepton and jet energy scales are propagated into the uncertainty on $E_{\text {T}}^{\text {miss}}$. A contribution to $E_{\text {T}}^{\text {miss}}$ also comes from energy deposits that are not associated with any identified physics object; uncertainties on the energy calibration (8 %) and resolution (3 %) of the sum of these deposits are also propagated to the uncertainty on $E_{\text {T}}^{\text {miss}}$ [91].

Uncertainties in the efficiency for tagging $b$-jets and in the rejection factor for light jets are determined from $t\bar{t}$ and dijet control samples  [81–83]. Additional uncertainties account for differences in $b$-tagging efficiency between simulated samples generated with sherpa and pythia and for differences observed between standard $b$-tagging and truth tagging (defined at the end of Sect. 4) for close-by jets [30].

The efficiencies for the lepton triggers in events with reconstructed leptons are nearly 100 %, and hence the related uncertainties are negligible. For the selection used in the $\nu \nu qq$ search, the efficiency for the $E_{\text {T}}^{\text {miss}}$ trigger is also close to 100 % with negligible associated uncertainties.

The merged-jet channel of the $$\ell \ell qq $$ search relies on measuring single-jet masses. To estimate the uncertainty in this measurement, jets reconstructed as described in Sect. 4 are compared with jets constructed using the same clustering algorithm but using as input charged-particle tracks rather than calorimeter energy deposits. The uncertainty is found using a procedure similar to that described in Ref. [92] by studying the double ratio of masses of jets found by both the calorimeter- and track-based algorithms: $$R^m_{\mathrm {trackcalo}} = r^{m,\mathrm {data}}_{\mathrm {trackcalo}} / r^{m,\mathrm {MC}}_{\mathrm {trackcalo}}$$, where $$r^{m,X}_{\mathrm {trackcalo}} = m^X_{\mathrm {calo}} / m^X_{\mathrm {track}}$$, $$X = $$ data or MC simulation, and $$m$$ is the jet mass. The uncertainty is taken as the deviation of this quantity from unity. Studies performed on dijet samples yield a constant value of $$10~\%$$ for this uncertainty. Applying the jet mass calibration derived from single jets in generic multijet samples to merged jets originating from boosted $$Z$$ bosons results in a residual topology-dependent miscalibration. This effect can be bounded by an additional uncertainty of $$10~\%$$. Adding these two effects in quadrature gives a total uncertainty on the jet mass scale of $$14~\%$$. The uncertainty on the jet mass resolution has a negligible effect on the final result.

### Signal acceptance uncertainty

The uncertainty in the experimental acceptance for the Higgs boson signal due to the modelling of Higgs boson production is estimated by varying parameters in the generator and re-applying the signal selection at generator level. The renormalization and factorization scales are varied up and down both independently and coherently by a factor of two; the amounts of initial- and final-state radiation (ISR/FSR) are increased and decreased separately; and the PDF set used is changed from the nominal CT10 to either MSTW2008 or NNPDF23.

### $ZZ^{(*)}$ background uncertainties

Uncertainties on the $ZZ^{(*)}$ background are treated as correlated between the $\ell \ell \ell \ell$ and $\ell \ell \nu \nu$ searches.

Uncertainties in the PDF and in $$\alpha _{\text {S}} $$ are taken from Ref. [93] and are derived separately for the $$q\bar{q}\rightarrow ZZ^{(*)} $$ and $$gg\rightarrow ZZ^{(*)} $$ backgrounds, using the envelope of the CT10, MSTW, and NNPDF error sets following the PDF4LHC prescription given in Refs. [94, 95], giving an uncertainty parameterized in $$m_{ZZ} $$. These uncertainties amount to 3 % for the $$q\bar{q}\rightarrow ZZ^{(*)} $$ process and 8 % for the $$gg\rightarrow ZZ^{(*)} $$ process and are found to be anti-correlated between the two processes; this is taken into account in the fit. The QCD scale uncertainty for the $$q\bar{q}\rightarrow ZZ^{(*)} $$ process is also taken from Ref. [93] and is based on varying the factorization and renormalization scales up and down by a factor of two, giving an uncertainty parameterized in $$m_{ZZ}$$ amounting to 4 % on average.

The deviation of the NLO electroweak $K$-factor from unity is varied up and down by 100 % in events with high QCD activity or with an off-shell $Z$ boson, as described in Ref. [96]; this leads to an additional overall uncertainty of 1–3 % for the $q\bar{q}\rightarrow ZZ^{(*)}$ process.

Full NLO and NNLO QCD calculations exist for the $gg\rightarrow h^{*} \rightarrow ZZ^{(*)}$ process, but not for the $gg\rightarrow ZZ^{(*)}$ continuum process. However, Ref. [97] showed that higher-order corrections affect $gg\rightarrow WW$ and $gg\rightarrow h^{*} \rightarrow WW$ similarly, within a 30 % uncertainty on the interference term. This yields about a 60 % uncertainty on the $gg\rightarrow WW$ process. Furthermore, Ref. [97] states that this conclusion also applies to the $ZZ^{(*)}$ final state, so the $gg$-induced part of the off-shell light Higgs boson $K$-factor from Ref. [38] is applied to the $gg\rightarrow ZZ^{(*)}$ background. The uncertainty on this $K$-factor depends on $m_{ZZ}$ and is about 30 %. An additional uncertainty of 100 % is assigned to this procedure; this covers the 60 % mentioned above. This uncertainty corresponds to the range considered for the $gg\rightarrow ZZ^{(*)}$ background $K$-factor in the ATLAS off-shell Higgs boson signal-strength measurement described in Ref. [96].

Acceptance uncertainties for the ggF and VBF (and *VH* for $\ell \ell \ell \ell$) channels due to the uncertainty on the $\le$1-jet and 2-jet cross-sections are estimated for the $q\bar{q}\rightarrow ZZ^{(*)}$ background by comparing the acceptance upon varying the factorization and renormalization scales and changing the PDF set. For $\ell \ell \ell \ell$ this leads to uncertainties of 4, 8, and 3 % on the ggF, VBF, and *VH* channels, respectively, where the uncertainty is fully anti-correlated between the ggF channel and the VBF and *VH* channels. For the $gg\rightarrow ZZ^{(*)}$ process where only LO generators are available, the VBF jets are simulated only in the parton shower, and so the acceptance uncertainty is estimated by taking the difference between the acceptances predicted by MCFM+Pythia8 and Sherpa, which have different parton shower simulations; this amounts to 90 % for the *VH* channel.

## Combination and statistical interpretation

The statistical treatment of the data is similar to that described in Refs. [98–102], and uses a simultaneous profile-likelihood-ratio fit to the data from all of the searches. The parameter of interest is the cross-section times branching ratio for heavy Higgs boson production, assumed to be correlated between all of the searches. It is assumed that an additional Higgs boson would be produced predominantly via the ggF and VBF processes but that the ratio of the two production mechanisms is unknown in the absence of a specific model. For this reason, fits for the ggF and VBF production processes are done separately, and in each case the other process is allowed to float in the fit as an additional nuisance parameter. The *VH* production mechanism is included in the fit for the $\ell \ell \ell \ell$ search and is assumed to scale with the VBF signal since both the *VH* and VBF production mechanisms depend on the coupling of the Higgs boson to vector bosons.

The simultaneous fit proceeds as follows. For each channel of each search, there is a distribution of the data with respect to some discriminating variable; these distributions are fitted to a sum of signal and backgrounds. The particular variables used are summarized in Table 3. The distributions for the $\ell \ell \ell \ell$ search are unbinned, since the resolution of $m_{\ell \ell \ell \ell }$ is very good, while other searches have binned distributions. For the VBF channels of the $\ell \ell \nu \nu$ search, only the overall event counts are used, rather than distributions, as the sample sizes are very small. The $\ell \ell qq$ and $\nu \nu qq$ searches include additional distributions in control regions in order to constrain the background, using either distributions of the mass variable or of the MV1c $b$-tagging category. The details of the specific variables used and the definitions of the signal and control regions are discussed in Sects. 5 to 8.

**Table 3:** Summary of the distributions entering the likelihood fit for each channel of each search, both in the signal region (SR) and the various control regions (CR) used to constrain the background. Each entry represents one distribution; some channels have several distributions for different lepton flavours. MV1c cat. refers to the MV1c $b$-tagging event category. The distributions are unbinned for the $\ell \ell \ell \ell$ search and binned elsewhere. The VBF channels of the $\ell \ell \nu \nu$ search use only the overall event counts. See the text for the definitions of the specific variables used as well as for the definitions of the signal and control regions

Search	Channel		SR	$Z$ CR	$W$ CR	Top CR
$\ell \ell \ell \ell$	ggF		$m_{eeee}$, $m_{\mu \mu \mu \mu }$, $m_{ee\mu \mu }$, $m_{\mu \mu ee}$			
	VBF		$m_{\ell \ell \ell \ell }$			
	*VH*		$m_{\ell \ell \ell \ell }$			
$\ell \ell \nu \nu$	ggF		$m_{\text {T}} ^{ee}$, $m_{\text {T}} ^{\mu \mu }$			
	VBF		$N_{\mathrm {evt}}^{ee}$, $N_{\mathrm {evt}}^{\mu \mu }$			
$\ell \ell qq$	ggF	Untagged	$m_{\ell \ell jj}$	MV1c cat.		
		Tagged	$m_{\ell \ell jj}$	MV1c cat.		$m_{\ell \ell jj}$
		Merged-jet	$m_{\ell \ell j}$	$m_{\ell \ell j}$		
	VBF		$m_{\ell \ell jj}$	$m_{\ell \ell jj}$		
$\nu \nu qq$	ggF	Untagged	$m_{\text {T}}$		MV1c cat. (0 $b$-tags)	
		Tagged	$m_{\text {T}}$		MV1c cat. (1 $b$-tag)	

As discussed in Sect. 9, the signal acceptance uncertainties, and many of the background theoretical and experimental uncertainties, are treated as fully correlated between the searches. A given correlated uncertainty is modelled in the fit by using a nuisance parameter common to all of the searches. The mass hypothesis for the heavy Higgs boson strongly affects which sources of systematic uncertainty have the greatest effect on the result. At lower masses, the $ZZ^{(*)}$ background theory uncertainties, the $Z+\mathrm {jets}$ modelling uncertainties, and the uncertainties on the jet energy scale dominate. At higher masses, uncertainties in the $\ell \ell \nu \nu$ non-$ZZ$ background, the jet mass scale, and the $Z+\mathrm {jets}$ background in the merged-jet regime dominate. The contribution to the uncertainty on the best-fit signal cross-section from the dominant systematic uncertainties is shown in Table 4.

**Table 4:** The effect of the leading systematic uncertainties on the best-fit signal cross-section uncertainty, expressed as a percentage of the total (systematic and statistical) uncertainty, for the ggF (left) and VBF (right) modes at $m_{H} =200$, 400, and $900~\mathrm{GeV}$. The uncertainties are listed in decreasing order of their effect on the total uncertainty; additional uncertainties with smaller effects are not shown

ggF mode		VBF mode	
Systematic source	Effect [%]	Systematic source	Effect [%]
$$m_{H} =200~\mathrm{GeV}$$			
$gg\rightarrow ZZ$ $K$-factor uncertainty	26.5	$gg\rightarrow ZZ$ acceptance	13.4
$Z~+$ hf $\Delta \phi$ reweighting	5.3	Jet vertex fraction ($\ell \ell qq$/$\nu \nu qq$)	13.4
Luminosity	5.2	$gg\rightarrow ZZ$ $K$-factor uncertainty	12.9
Jet energy resolution ($\ell \ell qq$/$\nu \nu qq$)	3.9	$Z+\mathrm {jets}$ $\Delta \phi$ reweighting	7.9
QCD scale $gg\rightarrow ZZ$	3.7	Jet energy scale $\eta$ modelling ($\ell \ell qq$/$\nu \nu qq$)	5.3
$$m_{H} =400~\mathrm{GeV}$$			
$qq\rightarrow ZZ$ PDF	20.8	$Z+\mathrm {jets}$ estimate ($\ell \ell \nu \nu$)	33.8
QCD scale $qq\rightarrow ZZ$	13.2	Jet energy resolution ($\ell \ell \ell \ell$/$\ell \ell \nu \nu$)	6.5
$Z+\mathrm {jets}$ estimate ($\ell \ell \nu \nu$)	12.6	VBF $Z+\mathrm {jets}$ $m_{\ell \ell jj}$	5.5
Signal acceptance ISR/FSR ($\ell \ell \ell \ell$/$\ell \ell \nu \nu$)	7.8	Jet flavour composition ($\ell \ell \ell \ell$/$\ell \ell \nu \nu$)	5.3
$Z+b\bar{b}$, $Z+c\bar{c}$, $p_{\text {T}}^{\ell \ell }$	5.6	Jet vertex fraction ($\ell \ell qq$/$\nu \nu qq$)	4.8
$$m_{H} =900~\mathrm{GeV}$$			
Jet mass scale ($\ell \ell qq$)	7	$Z+\mathrm {jets}$ estimate ($\ell \ell \nu \nu$)	19.2
$Z+jj$ $p_{\text {T}} ^Z$ shape ($\nu \nu qq$)	5.6	Jet mass scale ($\ell \ell qq$)	8.7
$qq\rightarrow ZZ$ PDF	4.3	$Z+jj$ $p_{\text {T}}^{\ell \ell }$ shape	7.3
QCD scale $qq\rightarrow ZZ$	3.5	Jet energy resolution ($\ell \ell \ell \ell$/$\ell \ell \nu \nu$)	4.4
Luminosity	2.6	Jet flavour composition ($VV$/Signal)	2.6

As no significant excess is observed, exclusion limits are calculated with a modified frequentist method [103], also known as $CL_s$, using the $\tilde{q}_{\mu }$ test statistic in the asymptotic approximation [104, 105]. The observed limits can be compared with expectations by generating ‘Asimov’ data sets, which are representative event samples that provide both the median expectation for an experimental result and its expected statistical variation in the asymptotic approximation, as described in Refs. [104, 105]. When producing the Asimov data set for the expected limits, the background-only hypothesis is assumed and the cross-sections for both ggF and VBF production of the heavy Higgs boson are set to zero. The remaining nuisance parameters are set to the value that maximizes the likelihood function for the observed data (profiled). When using the asymptotic procedure to calculate limits it is necessary to generate an Asimov data set both for the background-only hypothesis and for the signal hypothesis. When setting the observed limits, the cross-section for the other production mode not under consideration is profiled to data before generating the background-only Asimov data set.

## Results

**Fig. 12:**
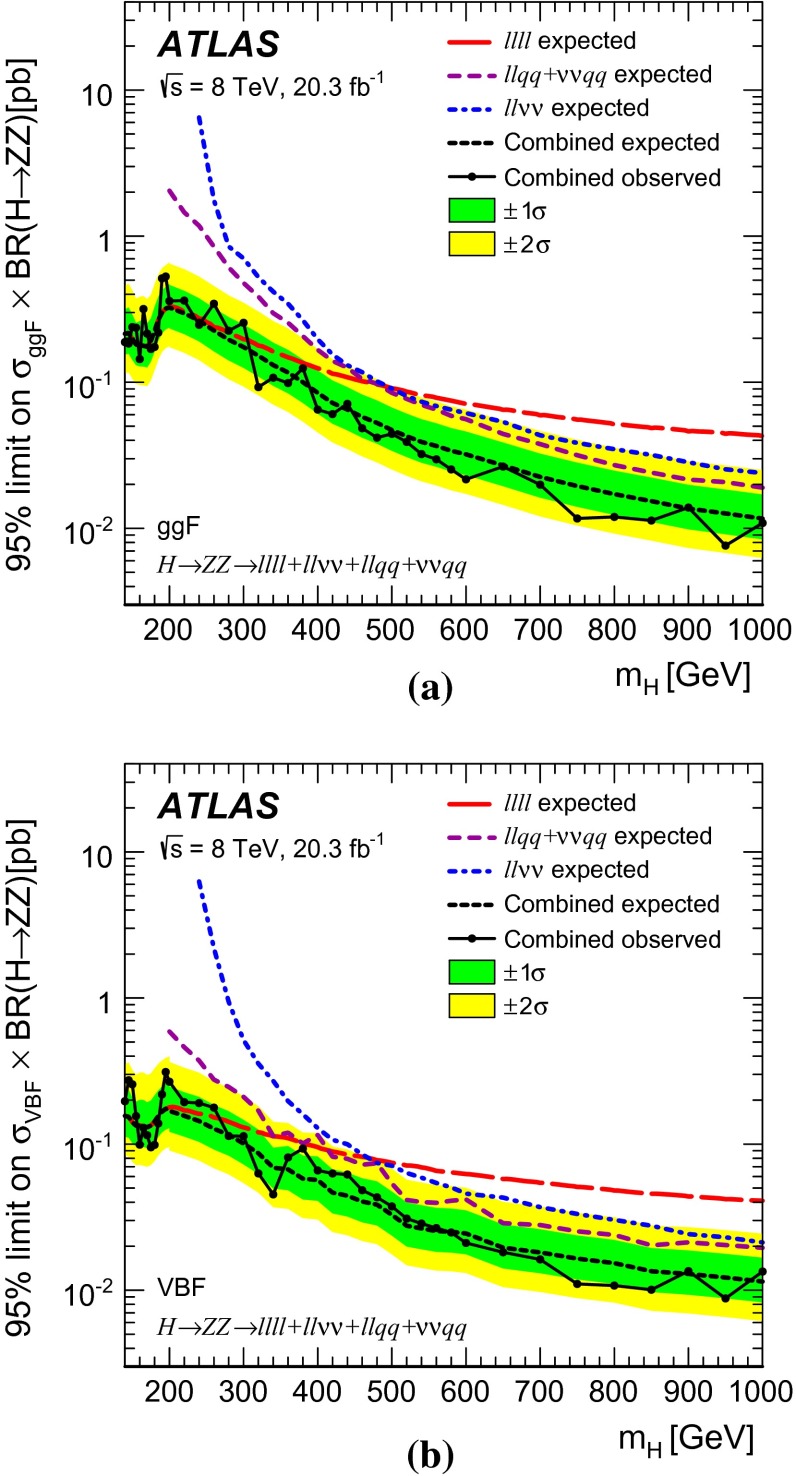
95 % CL upper limits on $\sigma \times {\mathrm {BR}}(H \rightarrow ZZ)$ as a function of $m_{H}$, resulting from the combination of all of the searches in the **a** ggF and **b** VBF channels. *The solid black line* and *points* indicate the observed limit. *The dashed black line* indicates the expected limit and the bands the 1-$\sigma$ and 2-$\sigma$ uncertainty ranges about the expected limit. *The dashed coloured lines* indicate the expected limits obtained from the individual searches; for the $\ell \ell qq$ and $\nu \nu qq$ searches, only the combination of the two is shown as they share control regions

Limits on the cross-section times branching ratio from the combination of all of the searches are shown in Fig. 12. Also shown are expected limits from the $\ell \ell \ell \ell$, $\ell \ell \nu \nu$ and the combined $\ell \ell qq$ +$\nu \nu qq$ searches (the latter two searches are only shown in combination as they share control regions). At low mass the $\ell \ell \ell \ell$ search has the best sensitivity while at high mass the sensitivity of the combined $\ell \ell qq$ +$\nu \nu qq$ search is greatest, with the sensitivity of the $\ell \ell \nu \nu$ channel only slightly inferior. In the mass range considered for this search the 95 % confidence level (CL) upper limits on the cross-section times branching ratio for heavy Higgs boson production vary between 0.53 pb at $m_{H} =195$ GeV and 0.008 pb at $m_{H} =950$ GeV in the ggF channel and between 0.31 pb at $m_{H} =195$ GeV and 0.009 pb at $m_{H} =950$ GeV in the VBF channel. The excursions into the $2\sigma$ band around the expected limit originate from local deviations in the input distributions. For example, the excess occurring around $200~\mathrm{GeV}$ and the deficit occurring around $300~\mathrm{GeV}$ arise from the $\ell \ell \ell \ell$ (see Fig. 1) search. Deficits at higher mass are driven by fluctuations in the $\ell \ell qq$ search (see Figs. 3 and 6).

**Fig. 13:**
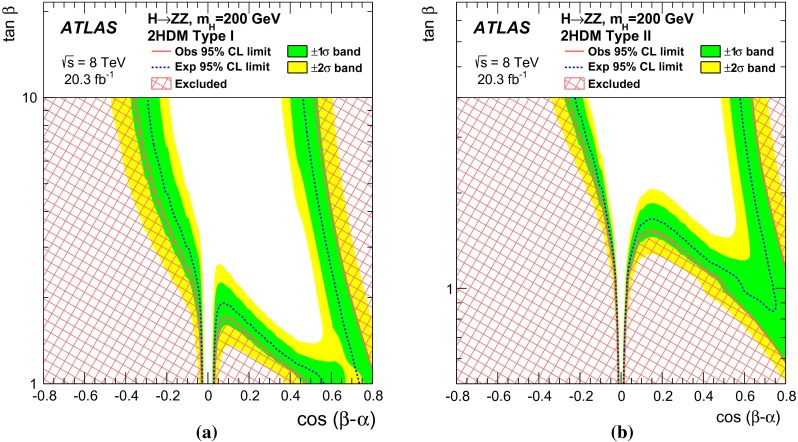
95 % CL exclusion contours in the 2HDM **a** Type-I and **b** Type-II models for $m_{H} =200~\mathrm{GeV}$, shown as a function of the parameters $\cos (\beta -\alpha )$ and $\tan {\beta }$. *The red hashed area* shows the observed exclusion, with *the solid red line* denoting the edge of the excluded region. *The dashed blue line* represents the expected exclusion contour and the shaded bands the 1-$\sigma$ and 2-$\sigma$ uncertainties on the expectation. *The vertical axis* range is set such that regions where the light Higgs couplings are enhanced by more than a factor of three from their SM values are avoided

**Fig. 14:**
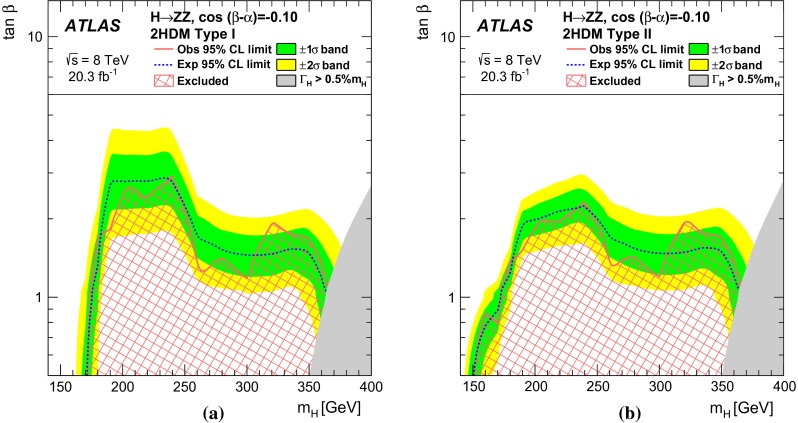
95 % CL exclusion contours in the 2HDM **a** Type-I and **b** Type-II models for $$\cos (\beta -\alpha )=-0.1$$, shown as a function of the heavy Higgs boson mass $$m_{H} $$ and the parameter $$\tan {\beta }$$. *The shaded area* shows the observed exclusion, with *the black line* denoting the edge of the excluded region. *The blue line* represents the expected exclusion contour and *the shaded bands* the 1-$$\sigma $$ and 2-$$\sigma $$ uncertainties on the expectation. *The grey area* masks regions where the width of the boson is greater than $$0.5~\%$$ of $$m_{H}$$. For the choice of $$\cos (\beta -\alpha )=-0.1$$ the light Higgs couplings are not altered from their SM values by more than a factor of two

Figure 13 shows exclusion limits in the $$\cos (\beta - \alpha )$$ versus $$\tan \beta $$ plane for Type-I and Type-II 2HDMs, for a heavy Higgs boson with mass $$m_{H} =200$$ GeV. This $$m_{H}$$ value is chosen so the assumption of a narrow-width Higgs boson is valid over most of the parameter space, and the experimental sensitivity is at a maximum. As explained in Sect. 3.2, the range of $$\cos (\beta - \alpha )$$ and $$\tan \beta $$ explored is limited to the region where the assumption of a heavy narrow-width Higgs boson with negligible interference is valid. When calculating the limits at a given choice of $$\cos (\beta -\alpha )$$ and $$\tan {\beta }$$, the relative rate of ggF and VBF production in the fit is set according to the prediction of the 2HDM for that parameter choice. Figure 14 shows exclusion limits as a function of the heavy Higgs boson mass $$m_{H} $$ and the parameter $$\tan {\beta }$$ for $$\cos (\beta -\alpha )=-0.1$$. The white regions in the exclusion plots indicate regions of parameter space not excluded by the present analysis; in these regions the cross-section predicted by the 2HDM is below the experimental sensitivity. Compared with recent studies of indirect limits [106], the exclusion presented here is considerably more stringent for Type-I with $$\cos (\beta -\alpha )<0$$ and $$1<\tan \beta <2$$, and for Type-II with $$0.5<\tan \beta <2$$.

The previously published ATLAS results using data collected at $\sqrt{s}=7~\mathrm{TeV}$ [5–7] assumed a SM Higgs boson with the relative rate of ggF and VBF production fixed to the SM prediction. Thus, they are not directly comparable with the current results, which assume that the heavy Higgs boson has a narrow width but also allow the rates of ggF and VBF production to vary independently. These results are also not directly comparable with the recent results published by the CMS Collaboration [8] for similar reasons.

## Summary

A search is presented for a high-mass Higgs boson in the $$H\rightarrow ZZ\rightarrow \ell ^+\ell ^-\ell ^+\ell ^-$$, $$H\rightarrow ZZ\rightarrow \ell ^+\ell ^-\nu \bar{\nu }$$, $$H\rightarrow ZZ\rightarrow \ell ^+\ell ^- q \bar{q}$$, and $$H\rightarrow ZZ\rightarrow \nu \bar{\nu } q \bar{q}$$ decay modes using the ATLAS detector at the CERN Large Hadron Collider. The search uses proton–proton collision data at a centre-of-mass energy of $$8~\mathrm{TeV}$$ corresponding to an integrated luminosity of 20.3 fb$$^{-1}$$. The results of the search are interpreted in the scenario of a heavy Higgs boson with a width that is small compared with the experimental mass resolution. The Higgs boson mass range considered extends up to $$1~\mathrm{TeV}$$ for all four decay modes and down to as low as $$140~\mathrm{GeV}$$, depending on the decay mode. No significant excess of events over the Standard Model prediction is found. Limits on production and decay of a heavy Higgs boson to two $$Z$$ bosons are set separately for gluon-fusion and vector-boson-fusion production modes. For the combination of all decay modes, 95 % CL upper limits range from 0.53 pb at $$m_{H} =195$$ GeV to 0.008 pb at $$m_{H} =950$$ GeV for the gluon-fusion production mode and from 0.31 pb at $$m_{H} =195$$ GeV to 0.009 pb at $$m_{H} =950$$ GeV for the vector-boson-fusion production mode. The results are also interpreted in the context of Type-I and Type-II two-Higgs-doublet models, with exclusion contours given in the $$\cos (\beta -\alpha )$$ versus  $$\tan \beta $$ and $$m_{H} $$ versus  $$\tan \beta $$ planes for $$m_{H} =200~\mathrm{GeV}$$. This $$m_{H}$$ value is chosen so that the assumption of a narrow-width Higgs boson is valid over most of the parameter space, and so that the experimental sensitivity is at a maximum. Compared with recent studies of indirect limits, the two-Higgs-doublet model exclusion presented here is considerably more stringent for Type-I with $$\cos (\beta -\alpha )<0$$ and $$1<\tan \beta <2$$, and for Type-II with $$0.5<\tan \beta <2$$.

## Figures and Tables

**Fig. 15:**
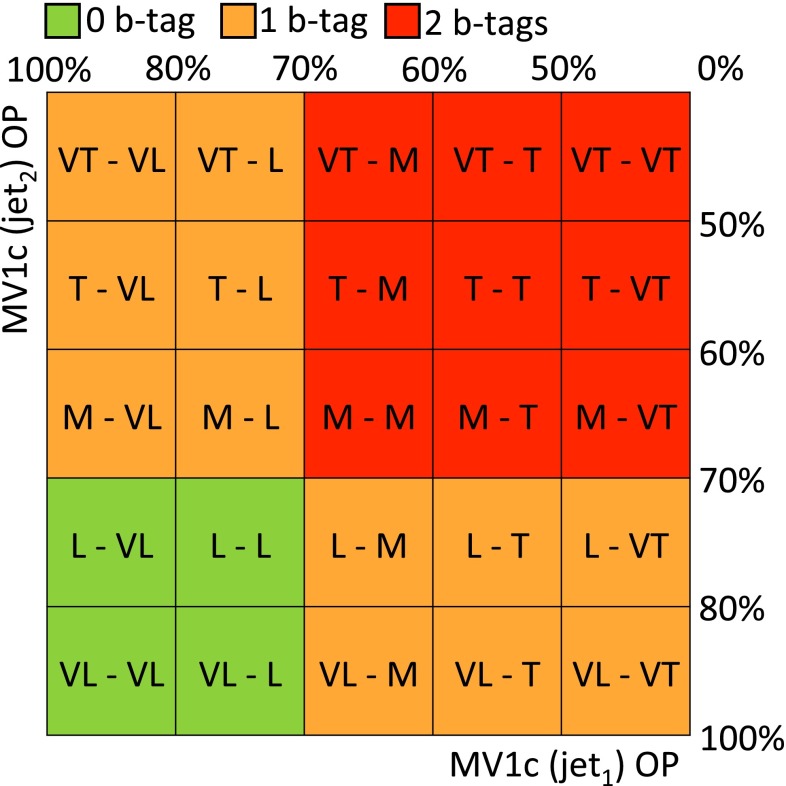
Event categorization as a function of the output of the MV1c $b$-tagging algorithm for the two signal jets. The bin boundaries correspond to the operating points (MV1c(jet) OP) giving $b$-tagging efficiencies of 100, 80, 70, and 50 %; i.e., the $b$-jet purity increases from *left* (*bottom*) to *right* (*top*). The event categories are labelled VL, L, M, T, and VT according to the definitions in the text, and the *different colours* correspond to events with 0, 1, and 2 identified $b$-jets

**Fig. 16:**
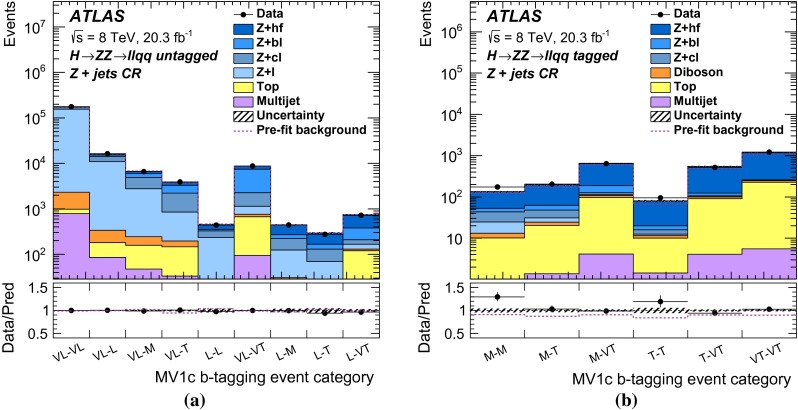
The distribution of the MV1c $b$-tagging event categories, based on the two signal jets, in the $Z+\mathrm {jets}$ control region in the **a** untagged ggF and **b** tagged ggF channels of the $H\rightarrow ZZ\rightarrow \ell ^+\ell ^- q \bar{q}$ search. The $b$-jet purity generally increases from *left* to *right*. *The dashed line* shows the total background used as input to the fit. The contribution labelled as ‘Top’ includes both the $t\bar{t}$ and single-top processes. *The bottom panes* show the ratio of the observed data to the predicted background

**Fig. 17:**
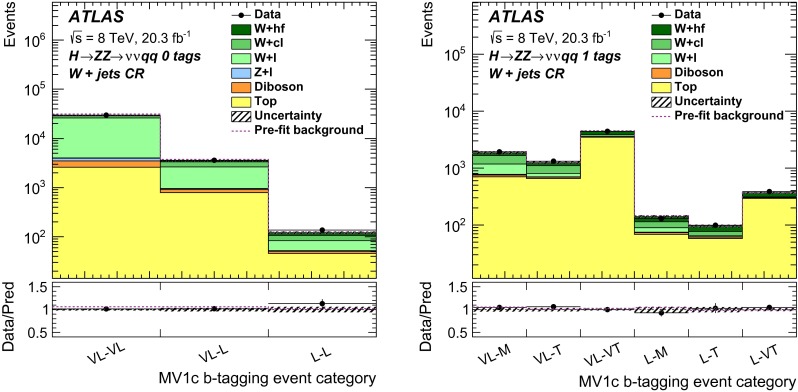
The distribution of the MV1c $b$-tagging event categories, based on the two signal jets, in the $W+\mathrm {jets}$ **a** 0-$b$-tag and **b** 1-$b$-tag control regions of the $H\rightarrow ZZ\rightarrow \nu \bar{\nu } q \bar{q}$ search. The $b$-jet purity generally increases from *left* to *right*. The dashed line shows the total background used as input to the fit. The contribution labelled as ‘Top’ includes both the $t\bar{t}$ and single-top processes. *The bottom panes* show the ratio of the observed data to the predicted background

## Appendix A: Flavour tagging in the $\ell \ell qq$ and $\nu \nu qq$ searches

In order to constrain the normalizations of the various flavour components of the $Z$+jets ($Z+jj$, $Z+cj$, $Z+bj$, and $Z+$hf) and $W+\mathrm {jets}$ ($W+jj$ and $W+cj$) backgrounds in the $\ell \ell qq$ and $\nu \nu qq$ searches, it is necessary to distinguish the different combinations of jet flavour. This is achieved by combining the information from the MV1c $b$-tagging discriminant of the two signal jets in order to disentangle the different light- and heavy-flavour components.

Besides the MV1c selection criterion described in Sect. 4, which had an average efficiency of 70 % for jets with $p_{\text {T}} >20~\mathrm{GeV}$ containing $b$-hadrons ($b$-jets), additional criteria, or operating points, are defined with average efficiencies of 80, 60, and 50 %. The efficiencies for accepting $c$-jets or light-quark jets for the 50 % (80 %) operating point are 1/29 (1/3) and 1/1400 (1/30), respectively. Based on these operating points, five bins in MV1c are defined:

**Table:**

Bin	$b$-Tagging efficiency (%)
Very loose (VL)	${>}80$
Loose (L)	$80{-}70$
Medium (M)	$70{-}60$
Tight (T)	$60{-}50$
Very tight (VT)	${<}50$

In this analysis, jets selected by the M, T, or VT operating points (i.e. ${>}70~\%$ efficiency for $b$-jets) are considered as $b$-tagged. Events are then categorized based on the combination of the binned MV1c operating points for the two signal jets, as shown in Fig. 15, in order to obtain optimal separation of the flavour components.

Distributions of the resulting MV1c event categories are shown in Figs. 16 and 17 for the $\ell \ell qq$ $Z+\mathrm {jets}$ and $\nu \nu qq$ $W+\mathrm {jets}$ control regions, respectively. These distributions are provided as input to the simultaneous profile-likelihood-ratio fit described in Sect. 10 in order to determine the normalization of the background flavour components defined above. Following the fit, the data are well-described by the MC simulation.

## Appendix B: Corrections to MC simulation for the $\ell \ell qq$ search

In order to improve the description of the data in the resolved ggF channel, corrections are applied to the Sherpa $Z+\mathrm {jets}$ simulation (prior to the likelihood fit) as a function of the azimuthal angle between the two signal jets, $\Delta \phi _{jj}$, and the transverse momentum of the leptonic $Z$ boson, $p_{\text {T}}^{\ell \ell }$, following Ref. [30]. The simulation does not model well the observed $\Delta \phi _{jj}$ distribution in the untagged control regions for $p_{\text {T}}^{\ell \ell } < 120~\mathrm{GeV}$; this is not seen at higher $p_{\text {T}}^{\ell \ell }$ or in the tagged control region. In order to improve the modelling, the $Z+jj$ component of the background with $p_{\text {T}}^{\ell \ell } <120~\mathrm{GeV}$ is scaled by a linear function derived from the control region with no $b$-tagged jets at low $p_{\text {T}}^{\ell \ell }$ with non-$Z$ boson backgrounds subtracted. Half the value of the correction is taken as a systematic uncertainty where it is applied. In the $Z+$hf sample with $p_{\text {T}}^{\ell \ell } <120~\mathrm{GeV}$, the full value of the correction is taken as an uncertainty. For $p_{\text {T}}^{\ell \ell } >120~\mathrm{GeV}$, no correction is applied for any sample. In this region, a linear fit is performed to the data/MC ratio of $\Delta \phi _{jj}$ in the untagged subchannel after subtracting the small non-$Z$ background, and the uncertainty on the fitted slope taken as an uncertainty for all $Z+\mathrm {jets}$ samples. Following this correction, the description of the $p_{\text {T}}^{\ell \ell }$ distribution in the control region with no $b$-tagged jets also improves, but there is still some residual discrepancy seen in the control regions that have $b$-tagged jets. Thus, the $Z+$hf background component is scaled by a function logarithmic in $p_{\text {T}}^{\ell \ell }$, determined from the combination of the control regions with one or more $b$-tagged jets (after subtracting the $Z+jj$ and non-$Z+\mathrm {jets}$ background components). An uncertainty of half this correction is applied for all $Z+\mathrm {jets}$ channels. (All these uncertainties are taken to be uncorrelated between the $Z+\text {light-jet}$ and $Z+$hf samples.) Following these corrections, the simulation models both the $\Delta \phi _{jj}$ and $p_{\text {T}}^{\ell \ell }$ distributions well in all $Z+\mathrm {jets}$ control regions.

For the VBF channel, no significant differences are seen in the $\Delta \phi _{jj}$ and $p_{\text {T}}^{\ell \ell }$ distributions, but there is a small difference in the $m_{\ell \ell jj}$ distribution in the control region. The simulated $Z+\mathrm {jets}$ background is corrected for this bin-by-bin and the full value of this correction is taken as an uncertainty, again uncorrelated between light- and heavy-flavour samples. No corrections are needed for the merged-jet ggF channel given the small sample size available.

It has been observed in an unfolded measurement of the $p_{\text {T}}$ distribution of $t\bar{t}$ quark pairs that the simulation does not accurately describe the $p_{\text {T}}^{t\bar{t}}$ distribution [107]. To correct for this, $t\bar{t}$ MC events are weighed by a function of $p_{\text {T}}^{t\bar{t}}$ taken from $7~\mathrm{TeV}$ data from Ref. [107] in order to make the simulation match the data. The correction is validated for $8~\mathrm{TeV}$ data using the $e\mu$ top-quark control region, and the uncertainty in this correction is estimated by varying it from 50 to 150 % of its nominal value.

## Footnotes

1 ATLAS uses a right-handed coordinate system with its origin at the nominal interaction point (IP) in the centre of the detector and the $z$-axis coinciding with the axis of the beam pipe. The $x$-axis points from the IP towards the centre of the LHC ring, and the $y$-axis points upward. Cylindrical coordinates ($r$,$\phi$) are used in the transverse plane, $\phi$ being the azimuthal angle around the $z$-axis. The pseudorapidity is defined in terms of the polar angle $\theta$ as $\eta = - \ln \tan (\theta /2)$. The distance in ($\eta$,$\phi$) coordinates, $\Delta R = \sqrt{(\Delta \phi )^2 + (\Delta \eta )^2}$, is also used to define cone sizes. Transverse momentum and energy are defined as $p_{\text {T}} = p \sin \theta$ and $E_{\text {T}} = E \sin \theta$, respectively.

2 The VBF channel is inclusive in quark flavour and hence dominated by the $Z$ + light-quark jet background.

3 The background samples that use the parameterized fast simulation are: Sherpa $W/Z+\mathrm {jets}$ production with $p_{\text {T}} ^{W/Z}<280~\mathrm{GeV}$ (for higher $p_{\text {T}} ^{W/Z}$ the full simulation is used since it improves the description of the jet mass in the merged $\ell \ell qq$ search described in Sect. 7.1.2); Powheg-Box $t\bar{t}$, single top, and diboson production; and SM Pythia $q\bar{q} \rightarrow Zh$ and Powheg-Box $gg \rightarrow Zh$ production with $h \rightarrow bb$. The remaining background samples and the signal samples, with the exception of those used for the $\nu \nu qq$ search, use the full Geant  4 simulation.
